# Pattern recognition receptor-associated immuno-thrombotic transcript changes in platelets and leukocytes with COVID19

**DOI:** 10.1371/journal.ppat.1013413

**Published:** 2025-08-18

**Authors:** Heather Learnard, Jason Core, Heather Corkrey, Anthony Sciaudone, Jeffrey Rade, Hardy Kornfeld, Jennifer P. Wang, Jane E. Freedman, Kahraman Tanriverdi, Milka Koupenova

**Affiliations:** 1 Department of Medicine, Divisions of Cardiovascular Medicine, UMass Chan Medical School, Worcester, Massachusetts, United States of America; 2 Department of Medicine, Internal Medicine Program, UMass Chan Medical School, Worcester, Massachusetts, United States of America; 3 Department of Medicine, Division of Pulmonary, Allergy, and Critical Care, UMass Chan Medical School, Worcester, Massachusetts, United States of America; 4 Department of Medicine, Division of Infectious Diseases and Immunology and Diabetes Center of Excellence, UMass Chan Medical School, Worcester, Massachusetts, United States of America; 5 Department of Medicine, Division of Cardiovascular Medicine, Vanderbilt Medical Center, Nashville, Tennessee, United States of America; Cleveland Clinic Foundation: Cleveland Clinic, UNITED STATES OF AMERICA

## Abstract

Respiratory infections are characterized by an increased risk of thrombosis, likely involving platelet-leukocyte crosstalk via pattern recognition receptors (PRRs). Here we characterized COVID19-mediated changes in PRR levels and their associations with thrombotic/coagulation-related transcriptional programs across platelets and leukocytes and assessed their correlation with COVID19 outcomes. Amplicon RNAseq of platelets and leukocytes from COVID19 patients (n = 10) and non-infected donors (n = 15) showed distinct patterns of PRR-expression levels based on cell type. Platelets from non-infected donors expressed TLR9 > RIG-I> CGAS at the highest level while leukocytes expressed TLR4 > TLR8 > RIG-I. COVID19 resulted in increased levels of TLR9, RIG-I, CGAS, and TLR1 in platelets and decreased levels of TLR6 and TLR8 in leukocytes, while the levels of the highest expressed PRRs remained almost unchanged. In platelets from COVID19 patients, MDA5, RIG-I, and LGP2 showed the highest associations with thrombotic-, coagulation-, and thrombolysis-associated transcripts, while in non-infected donors, TLR9 showed the highest associations with those transcripts. In leukocytes, RIG-I and MDA5 also correlated with coagulation-related transcripts when derived from the non-infected donors, but those associations were almost lost with COVID19. Platelet-leukocyte aggregates increased with COVID19 as did extracellular vesicles detected by imaging cytometry, immunofluorescence, or electron microscopy. Platelet-TLR3 and leukocyte-TLR5 positively correlated with severity and survival of the COVID19 patients, while leukocyte-TLR7 showed an inverse correlation. Coagulopathy, measured by INR, was associated with platelet-TLR4 and leukocyte-TLR10. Liver inflammation, assessed by ALT levels, correlated with platelet- and leukocyte-LGP2, in addition to leukocyte-TLR3, -TLR6, -TLR7, and -RIG-I. Analysis of publicly available whole-blood-RNAseq, showed that COVID19 and tuberculosis were more similar than COVID19 and influenza with respect to associations between PRRs and thrombotic/coagulation-related transcripts. Overall, platelets and leukocytes exhibit distinct patterns of PRR expression and correlations with thrombotic/coagulation-related transcripts that change with COVID19, and there are distinct PRRs in each cell population that associate with COVID19 severity, coagulopathy, and liver damage.

## Author summary

Respiratory infections like COVID19 increase the risk of blood clots. Platelets and white blood cells (leukocytes) can be major contributors through their pathogen-activated receptors, called pattern recognition receptors (PRRs). We studied how PRRs and clotting-related genes associate and change in COVID19 as well as how PRRs from each cell type correlate to COVID19 severity, survival, and ability to form blood clots. Finally, we compared associations between PRRs and clotting-related genes in COVID19 to changes related to influenza and tuberculosis (TB). We used platelets and leukocytes taken from the same individuals and found that they have different patterns of pathogen receptor levels. COVID19 altered the PRR expression and its links to clotting genes. Interestingly, genes related to clotting had higher levels in platelets, while pathogen receptors and anticoagulation genes were higher in leukocytes. COVID19 altered PRR and clotting gene interactions in blood, showing more similarity to TB than to influenza. Regarding COVID19 outcomes, platelet-TLR4 and leukocyte-TLR5 levels correlated with more severe COVID19 outcomes, while leukoctye-TLR7 levels increased and were associated with better outcomes. Additionally, increased coagulopathy was associated with platelet-TLR4 and leukocyte-TLR10. Overall, our study suggests that pathogen receptor expressions change differently in platelets and leukocytes and should be analyzed as a group rather than as individual receptors.

## Introduction

Respiratory infections caused by viruses such as influenza and SARS-CoV-2 (the viral pathogen responsible for COVID19) are strongly associated with increased inflammation, vessel damage, and consequent thrombotic outcomes usually manifesting in acute myocardial infarction, venous thromboembolism (VTE), and stroke [1,2]. Increased risk for thrombotic outcomes is not specific to viral pathogens and has also been observed with bacteria such as *Mycobacterium tuberculosis* (TB) [3]. Platelets are the blood components that act as the central mediator of hemostasis in response to vascular injury and endothelial disruption [4]. However, this highly specialized blood component also serves roles in immune surveillance and initiation of the inflammatory response [5,6].

Although the mechanisms underlying pulmonary infection-mediated thrombosis are not well understood, platelets in combination with surrounding leukocytes and plasma factors are suspected to be key drivers of immune-mediated thrombotic outcomes, or immunothrombosis. Here, immunothrombosis refers to the contribution of immune receptors and the initiated immune response to thrombosis, inflammation, and clot formation in the vasculature. The host uses various systems to protect itself from harmful and invasive pathogens. Pathogen-associated molecular pattern (PAMP) receptors, also known as pattern recognition receptors (PRRs), comprise a rapid response defense system against pathogenic invaders. PRRs include the Toll-like receptors (TLRs) and retinoic acid-inducible gene I (RIG-I)-like receptors (RLRs) [7]. Humans have a total of ten TLRs (TLR1–10) and their major role in the body is to mediate a pathogen response by increasing cytokine production, inducing antigen presentation, and ultimately activating the adaptive immune system [7]. RLRs located in the cytoplasm, such as LGP2, RIG-I, and MDA5, can also sense pathogen-derived RNA-molecular patterns, whereas cytoplasmic CGAS senses foreign and mitochondrial-DNA [8]. With respect to SARS-CoV-2, viral RNA (vRNA) can be sensed by TLR7, TLR8, and during replication, by MDA5 and TLR3 [9–11]. RIG-I is another vRNA sensing receptor that has been mostly associated with influenza vRNA [12,13]. Collectively, the ultimate function of these cell PRRs is to protect the host from infection and limit pathogen dissemination and propagation with as little consequence to the host as possible. Although the process of defense, involving inflammation, cytokine release and cell death, is important for limiting viral replication, it may also underlie and contribute to thrombotic and microthrombotic events.

Platelets contain the transcripts for all known TLRs [4], suggesting that their involvement in the innate immune response may extend to recognizing a broad range of infectious pathogens. Stimulation of TLRs induces platelet activation, aggregation, and granule secretion, suggesting involvement of PRRs in platelet-driven prothrombotic and inflammatory signaling during periods of infection [14]. Internalization of SARS-CoV-2 virions by platelets has been shown to activate the caspase system and induce pathways that mediate necroptosis [15], a variant of programmed cell death, which can trigger a prothrombotic response during sepsis [16]. Influenza has also been shown to be internalized by platelets and its vRNA is recognized by TLR7 leading to NETosis [17] which is essential to initiate an immune response, but dysregulation of the process can increase the risk for thrombotic outcomes [17]. TB is another pulmonary infection associated with thrombosis and increased prevalence of VTE and pulmonary embolism in patients [18]. Overall, pathogens that infect the lungs and cause thrombosis indicate a broad platelet response during infection.

In this study, we examine changes in PRRs and thrombotic- or coagulation-related programs in platelets and leukocytes isolated from the same donors and how the relationship between these programs changes with SARS-CoV-2 infection. The major goals of our study are to uncover unknown changes in platelets and leukocytes that may influence the coagulation-mediated response during infection and to identify mechanistic targets for potential therapeutic intervention. We assessed changes in the expression programs of genes related to thrombosis, coagulation, and platelet-leukocyte interactions as a function of PRR expression and infection in healthy and COVID19 patients. We then repeated this analysis with publicly available whole blood RNAseq profiles of patients infected with COVID19, TB, or influenza for comparison. This study is the first to provide evidence of expression programs in leukocytes potentially mediating and/or contributing to a dysregulated coagulation response as well as providing evidence for a connection to platelet-leukocyte crosstalk. Our study also reports that correlations of PRRs and thrombotic programs in the blood profile of COVID19 patients are more closely related to the blood profile of acute TB rather than influenza patients. Our study highlights the importance of addressing PRR pathways during infection as possible contributors to thrombotic complications that may be best targeted for prevention or mitigation by novel therapies that differ from classical antiplatelet drugs.

## Results

### Levels of PRR expression differ between platelets and leukocytes

**Fig 1:**
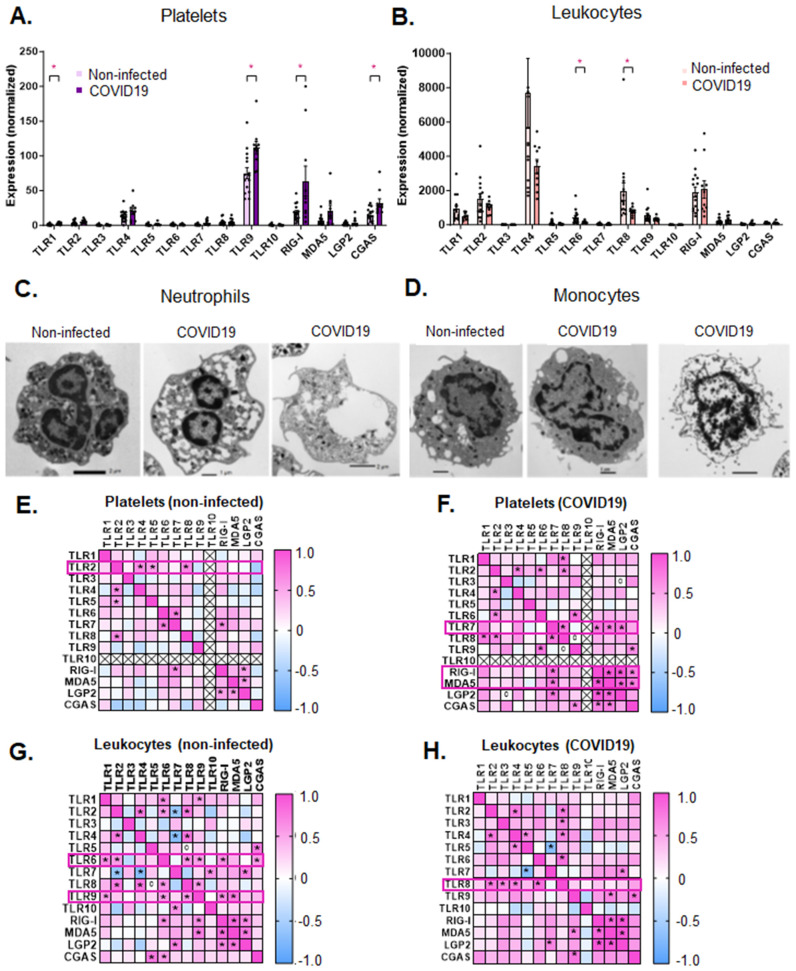
PRR expression in platelets and leukocytes and their changes with SARS-CoV-2 infection. Platelets and leukocytes were isolated from patients as described in the Methods. PRR gene expression levels assessed by RNAseq and normalized to three housekeeping genes in **A.** platelets and **B.** leukocytes from non-infected donors and COVID19 patients. Transmission electron microscopy (TEM) of **C.** neutrophils and **D.** monocytes in the leukocyte fraction of non-infected donor and COVID19 patient samples showing loss of granularity and lack of nuclei in neutrophils in addition to damage of the surface membrane or lytic cell death in monocytes. Heat maps of co-expression of PRRs in: **E.** platelets of non-infected donors versus **F.** platelets from COVID19 patients and in **G.** leukocytes from non-infected donors and **H.** leukocytes from COVID19 patients. Data are represented as mean ± SEM; statistical significance in A. and B. was determined by a Mann-Whitney test for each gene with *p < 0.05. Heat maps of Spearman correlation coefficients and their significance indicated by * (p < 0.05) in each cross-correlation were generated in GraphPad. Receptors with the most cross-correlations with other PRRs are noted in the magenta boxes.

Human platelets are predominantly studied for their function related to hemostasis and thrombosis and the thrombotic receptors and proteins that mediated these outcomes; however, platelets also express PRRs such as TLRs [4] and RLRs [5,19] similar to professional immune cells in blood. It is not known whether the expression level of platelet-PRRs is comparable to circulating blood-leukocyte-PRRs. To address this question, we isolated platelets and leukocytes from the same non-infected donors and subjected them to RNAseq. In order to compare the PRR transcript levels between platelets and leukocytes, we normalized each PRR-transcript of interest to three housekeeping genes for the same donor (see Methods [20]). Our analysis shows that in non-infected donors, platelet-PRRs are expressed at much lower levels than blood leukocyte-PRRs (Fig 1A and 1B), as the fold difference for each PRR varies between the two blood populations, with a six-fold difference for TLR9 and close to a thousand-fold difference for TLR1 (average fold difference is ~ 200, Fig 1A and 1B). With COVID19 infection the fold-difference in expression between the two blood components decreases, but overall leukocytes continue to express, on average, approximately three-fold higher levels for all PRRs. Interestingly, patterns of PRRs with the highest expression also differed between the two populations. PRRs with the highest expression in platelets are (in order from highest to lowest) TLR9 > RIG-I> CGAS> TLR4 > MDA5 (Fig 1A), while in leukocytes the pattern is TLR4 > TLR8 > RIG-I > TLR2 > TLR1 (Fig 1B). Our data suggest that there is a vast difference in transcript levels of PRRs between platelets and leukocytes in non-infected donors in addition to the fact that the PRRs with the highest expression in platelets do not mirror those of leukocytes when both populations are isolated from the same donors.

### Levels of the highest expressed PRR change in both platelets and leukocytes with COVID19 infection, but with different patterns

Levels of specific PRRs change according to the pathogen, infection, infection stage, or immune response. COVID19 is caused by a positive-sense, single-stranded RNA virus, SARS-CoV-2 [1]. Different components of SARS-CoV-2 can be recognized by different PRRs depending on the level of initial exposure, cell compartment, and stage of infection of the host’s cell. Upon endocytosis of the virions, SARS-CoV-2 RNA can be recognized by TLR8 and some propose also TLR7 [1]. Escape from the endosomal-lysosomal compartment into the cytoplasm can lead to viral replication and recognition of double-stranded (ds)-SARS-RNA by MDA5 [11]. Evidence exists that surface components of SARS-CoV-2 can be recognized by TLR4 [21]. Whether platelets and leukocytes change their PRR-expression profile, in similar or distinct fashion, with SARS-CoV-2 infection is largely unknown. When compared to non-infected donors, platelets from COVID19 patients had significantly increased levels of TLR9, RIG-I, CGAS, and TLR1 (Fig 1A); while leukocytes did not show an increase in any of the PRRs (Fig 1B). In fact, blood leukocytes from COVID19 patients showed a decreased level of expression of TLR6 and TLR8 when compared to non-infected donors (Fig 1B). On the other hand, the highest expressed PRRs in both platelets and leukocytes did not change with COVID19 infection, however; the patterns of PRRs changed slightly. In platelets, MDA5 showed higher expression than TLR4 with the pattern: TLR9 > RIG-I> CGAS> MDA5 > TLR4 (Fig 1A); in leukocytes, RIG-I became the second most highly expressed transcript while TLR8 became the fourth most highly expressed and the pattern became TLR4 > RIG-I > TLR2 > TLR8 > TLR1 (Fig 1B).

Changes in platelets result from their precursors the megakaryocytes, which rapidly respond to replace lost platelets during infection; leukocytes, on the other hand, can change their own transcriptional profile [22]. To assess whether changes in gene expression were associated with the morphological changes in leukocytes, we examined the leukocyte fraction by transmission electron microscopy (TEM). This showed morphological changes indicative of cell death in monocytes and degranulation or enucleation of neutrophils from COVID19 patients when compared to the same cell populations in non-infected individuals (Fig 1C and 1D). These data suggest that reduced transcript levels in leukocytes may be due to cell death rather than a regulated reduction in expression. Of note, cell death pathways are also activated in platelets [15,23] but megakaryocytes may compensate by releasing new platelets. Our data provide evidence that platelets and leukocytes express PRRs in distinct patterns, and the net levels of those patterns change in the setting of SARS-CoV-2 infection.

### Co-expression of PRRs changes with SARS-CoV-2 infection in COVID19 patients

Previous studies have shown that certain surface and endosomal TLRs are co-expressed in human platelets [4]. However, it is not known whether co-expression patterns change with infection in either platelets or leukocytes from the same individuals. Correlation analysis of PRRs in platelets from non-infected individuals showed that TLR2 expression was positively associated with the most PRRs (TLR4, TLR5, and TLR8) (Fig 1E and S3 Table). Interestingly, in platelets from COVID19 patients, TLR2 continued to associate with TLR4 and TLR8, but associated with TLR6 rather than TLR5 (Fig 1E and 1F and S3 and S4 Tables). Additionally, in platelets, transcripts coding for vRNA-sensing PRRs, TLR7, RIG-I, MDA5, and LGP2 became co-expressed only with SARS-CoV-2 infection (Fig 1E and 1F and S3 and S4 Tables).

Blood leukocytes, on the other hand, showed different co-expression patterns than platelets both at baseline and with SARS-CoV-2 infection. In leukocytes from non-infected donors, TLR6 co-expressed with the most PRRs (TLR1, TLR2, TLR8, TLR9, RIG-I, and CGAS) followed by TLR9 (TLR1, TLR6, TLR8, RIG-I, and MDA5) (Fig 1G and S5 Table). In contrast to platelets, leukocytes from COVID19 patients showed reduced correlations among PRRs: TLR6 correlated only with TLR8; and TLR9 became associated with MDA5 and CGAS (Fig 1H and S6 Table). Similar to platelets, there were changed patterns in the correlations among PRRs in leukocytes from COVID19 patients as the PRR with the most associations became TLR8 (associated with TLR2, TLR3, TLR4, and TLR6) (Fig 1H). These data indicate that PRR expression changes, driven by SARS-CoV-2 infection, differs between platelets and leukocytes.

### PRR expression, in both platelets and leukocytes, correlates with thrombosis-, coagulation-, and platelet-immune cell-interaction-related transcripts and these correlations change with COVID19

**Fig 2:**
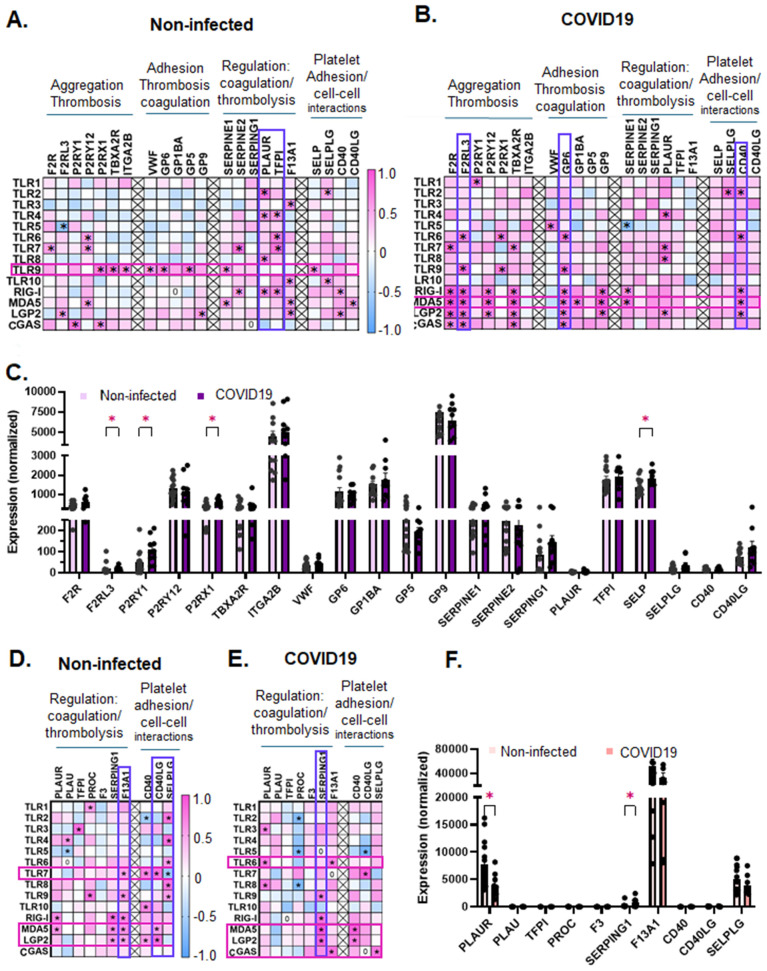
Infection-mediated changes in transcripts related to thrombosis, coagulation and platelet-leukocyte interactions in platelets and leukocytes. Platelets and leukocytes were isolated from the same non-infected donors or COVID19 patients and subjected to RNAseq as described in the Methods. Heat map of Spearman Correlations between PRRs and thrombotic, coagulation, and platelet-leukocyte interaction transcripts in **A.** platelets from non-infected donors and **B.** platelets from COVID19 patients. Significance is indicated by *(p < 0.05) in each cross-correlation. **C.** Platelet transcript changes between non-infected donors and COVID19 patients. Heat maps of Spearman correlation coefficients between PRRs and transcripts related to coagulation and platelet-leukocyte interactions in **D.** leukocytes from non-infected donors and **E.** leukocytes from COVID19 patients. **F.** Leukocyte gene expression levels in non-infected donors and COVID19 patients. Data in **C.**, and **E.**, are represented as mean ± SEM and statistical significance was determined by Mann-Whitney test for each gene with *p < 0.05. Spearman correlation coefficients were considered significant and are indicated by *, when p < 0.05.

An increased risk for thrombotic outcomes is associated with many respiratory infections [24,25], but it is unclear if the immune sensing-PRRs associate with changes in transcripts related to classical hemostasis and thrombosis, coagulation and thrombolysis, or with changes in cell-cell selectins or ligands. To investigate whether changes in PRR expression in platelets may be associated with changes in transcripts related to thrombosis, coagulation, and cell-cell interactions, we examined correlations between PRRs and the select gene transcripts representing each group as listed in Fig 2A and 2B (the function of each transcript-encoded-protein can be found in S2 Table). In platelets from non-infected individuals, TLR9 is associated with the most thrombotic, coagulation-related, and platelet-immune cell interaction-related transcripts (Fig 2A and S7 Table). Additionally, in platelets from non-infected individuals, tissue factor pathway inhibitor (TFPI) and urokinase plasminogen activator receptor (PLAUR) were positively associated with the most PRRs. TFPI was associated with TLR4, TLR5, TLR6, TLR7, and RIG-I, while PLAUR was associated with TLR2, TLR4, TLR8, and RIG-I (Fig 2A and S7 Table). In the platelets from COVID19 patients, MDA5 showed the highest association with thrombotic-, coagulation-, and platelet-leukocyte-interaction-related transcripts (Fig 2B and S8 Table); MDA5 codes for the cytoplasmic receptor that senses SARS-CoV-2 vRNA [26]. With COVID19 infection, F2RL3 (coding for thrombin receptor PAR4), and GP6 [27] (coding for a receptor for collagen and critical for collagen-mediated platelet aggregation) were associated with the most PRRs. Interestingly, for transcripts related to platelet-leukocyte mediated interactions, platelet-CD40, but not -SELP, -CD40LG or -SELPLG, were associated with the most PRRs in the COVID19-patients’ platelets (TLR2, TLR6, RIG-I, MDA5, and LGP2). We then assessed whether the levels of any of the selected transcripts changed with infection in platelets with SARS-CoV-2 infection. Our data show that platelets of COVID19 patients, compared to non-infected donors, had significantly greater expression of the thrombosis-related receptors F2RL3, P2RY1, P2RX1, and of the platelet-leukocyte interaction mediating SELP (Fig 2C).

In leukocytes, the patterns of associations between PRRs and coagulation- or leukocyte-platelet-interaction-related transcripts were reversed. In leukocytes of non-infected individuals, MDA5, LGP2, and TLR7 were associated with the most thrombosis-, coagulation-, and immune interaction-related transcripts, and all three were associated with F13A1 and CD40LG (Fig 2D and S10 Table). SELPLG (the receptor for P-selectin/SELP) was associated with the most PRRs; being positively associated with TLR2, TLR4, TLR6, TLR8, TLR9, and negatively associated with TLR7 (Fig 2D and S9 Table). In the leukocytes of COVID19 patients, no PRR associated with more than two of the selected transcripts, as TLR7, MDA5, and LGP2 reduced the number of correlations with selected transcripts (Fig 2E and S10 Table). Leukocyte-SERPING1 correlated with the most leukocyte-PRRs, including TLR9, RIG-I, MDA5, and LGP2 with SARS-CoV-2 infection (Fig 2E and S10 Table). Interestingly, PROC, coding for serine protease that regulates blood coagulation, correlated negatively with multiple PRRs including TLR2, TLR5, and TLR8 (Fig 2E and S10 Table). When we assessed changes in expression of the selected transcripts related to coagulation and cell-cell interactions, similar to PRRs in leukocytes, PLAUR levels were reduced with infection; however, contrary to PRRs, SERPING1 levels increased in the leukocytes from COVID19 patients when compared to non-infected donors (Fig 2F). Our results indicate that platelets and leukocytes have distinct patterns of co-expression between PRRs and prothrombotic, coagulation-, or platelet-immune cell-interaction-related transcripts that not only differ between platelets and leukocytes but also change with infection in COVID19 patients; however, in both cell populations, MDA5 and LPG2 (both involved in SARS-CoV-2-vRNA sensing) had the highest correlations with transcripts related to thrombosis, coagulation and thrombolysis, and platelet-immune cell interactions.

### Comparative analysis of expression of thrombotic and coagulation related transcripts in platelets versus leukocytes

**Fig 3:**
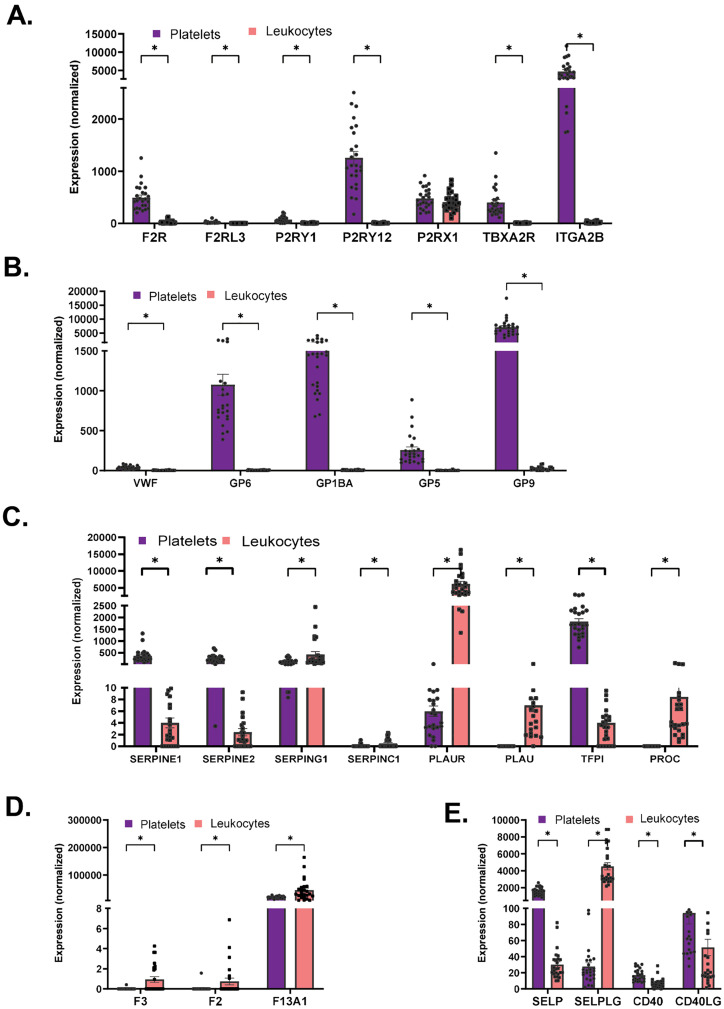
Comparative expression between platelets and leukocytes in levels of transcripts related to thrombosis, coagulation and platelet-leukocyte interactions. Platelets and leukocytes were isolated from the same non-infected donors or COVID19 patients and subjected to RNAseq as described in the Methods. COVID19-mediated changes between the two blood components of transcripts related to **A.** platelet thrombotic function, **B.** platelet adhesion function, **C.** coagulation and thrombolysis, **D.** platelet-leukocyte interactions, and **E.** cell-cell interaction gene related transcripts. Data are represented as mean ± SEM; statistical significance between non-infected donors and COVID19 patients was determined by Mann-Whitney test for each gene with *(p < 0.05).

Platelets are the blood component primarily responsible for thrombotic outcomes. Leukocytes can also increase transcription of tissue factors and other gene products that contribute to overall coagulation risk. Similarly, to our investigation of PRR expression across these two blood cell populations (Fig 1), we sought to compare overall expression of prothrombotic- and coagulation-related transcripts between platelets and leukocytes using normalized data. As expected, the platelet-specific transcripts related to platelet aggregation and adhesion, ITGA2B, SELP, VWF, P2RY12, GP1BA, GP6, GP5, and GP9, were highly expressed in platelets but not in leukocytes (Fig 3A and 3B). Expression levels of transcripts related to thrombosis, coagulation and thrombolysis could be separated into three subgroups. Group one included transcripts whose expression was highly increased only in platelets: SERPINE1, SERPINE2, and TFPI (Fig 3C). Group two included transcripts highly enriched in leukocytes but not expressed in platelets: PROC, PLAU, F3, F2, and SERPINC1 (Fig 3C and 3D). Group three is comprised of transcripts expressed in both populations but at higher levels in leukocytes: SERPING1, F13A1, and PLAUR (Fig 3C and 3D). With regard to transcripts coding for cell-cell interactions, platelets expressed higher levels of SELP, CD40, and CD40LG when compared to leukocytes, while leukocytes expressed higher levels of SELPLG when compared to platelets (Fig 3E). Overall, our data indicate that in addition to platelets, leukocytes contribute to the profile of coagulation-related transcripts.

**Fig 4:**
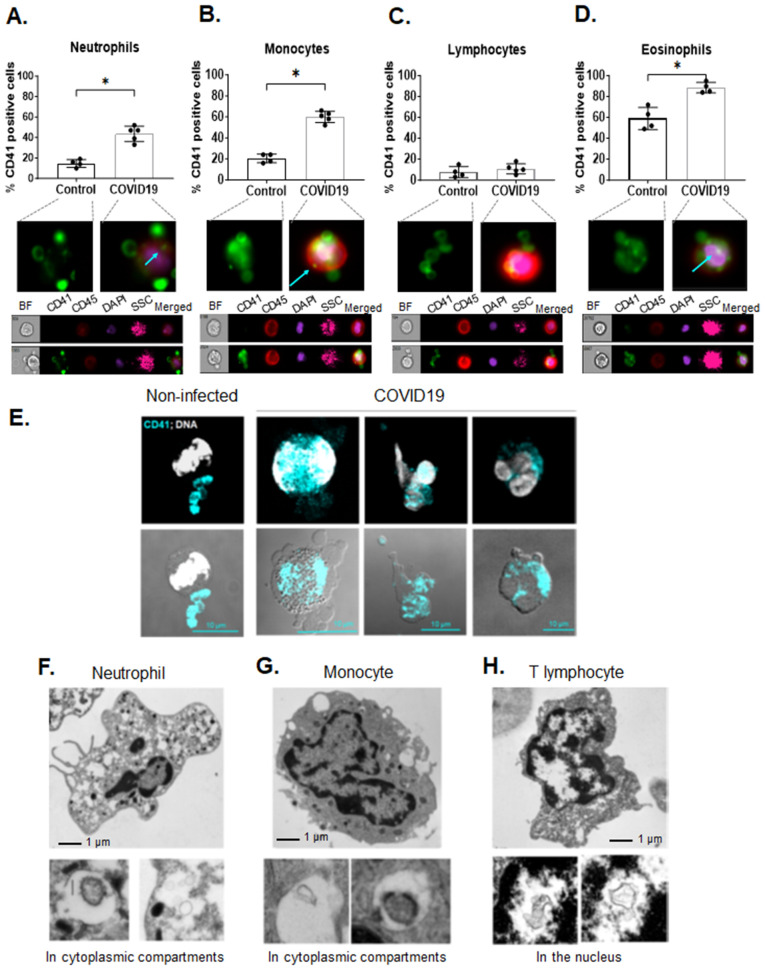
Low levels of platelet-specific transcripts in leukocytes can be due to platelet-leukocyte interactions. Whole blood isolated from age- and sex-matched, non-infected donors and COVID19 patients was fixed, stained, and subjected to AMNIS flow cytometry. Different blood populations were separated based on CD45 expression and side scatter intensity and CD41-platelets were analyzed in each separate population. Heterotypic aggregates of platelets with **A.** neutrophils, **B.** monocytes, **C.** pan lymphocytes, and **D.** eosinophils and the respective respresentative AMNIS-generated images. Data are represented as mean ± SEM; statistical significance between non-infected donors and COVID19 patients was determined by Mann-Whitney test with *p < 0.05. Confocal immunofluorescence images of whole blood indicating **E.** Platelet-derived CD41-microparticles inside leukocytes (representative of four different COVID19 patients and non-infected controls are shown). Transmission electron microscopy of the leukocyte fraction patients showing extracellular vesicles in **F.** neutrophils, **G.** monocytes, and **H.** lymphocytes. Images are representative of n=4 COVID19 patietns; images of non-infected control cells are included in Fig 1E.

It is important to acknowledge that a few of the highly expressed platelet transcripts were also detected in the leukocyte population. This is not surprising as heterotypic aggregates (HAGs) and microparticle exchange increases with age and infection. The age and comorbidities of the non-infected participants in our study matched the infected population (S1 Table). Blood from COVID19 patients contained platelet-leukocyte HAGs, as previously reported [28–30] (Fig 4A-4D), and leukocytes contained platelet-derived CD41-microparticles (Fig 4A-4E). Interestingly, extracellular vesicles in leukocytes were found not only in cytoplasmic vesicles (Fig 4F and 4G) but in the nucleus of a T cell (Fig 4H). TEM of the leukocyte population also showed platelets and leukocytes interacting together, explaining the inability to remove certain platelet transcripts from the leukocyte fraction (S1 Fig). Our data indicate that platelets form HAGs with all blood leukocyte populations, and platelet microparticles with CD41 are present in blood leukocytes, proposing that the observed platelet-specific RNA transcripts come from platelets.

### Cross-correlation analysis of PRRs from one blood cell population with immunothrombotic transcripts from the other cell population

**Fig 5:**
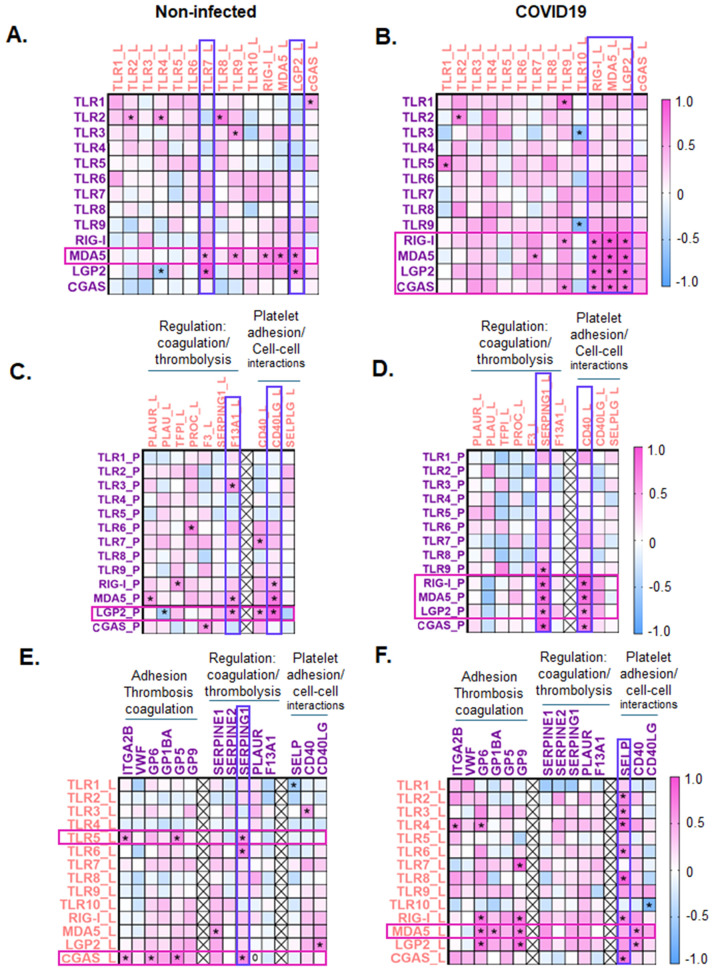
Cross-cell PRR-correlations with thrombotic- and coagulation-related transcripts between platelets and leukocytes. Platelets and leukocytes were isolated from the same non-infected donors or SARS-CoV-2-infected patients and subjected to RNAseq as described in the Methods. Correlations of platelet-PRRs (purple) with leukocyte-PRRs (orange) in **A.** non-infected donors and in **B.** COVID19 patients. Correlations of platelet-PRRs (purple) with leukocyte-thrombotic-coagulation-related transcripts in (orange) in **C.** non-infected donors and in **D.** COVID19 patients. Correlations of leukocyte-PRRs (orange) with platelet-thrombotic-coagulation related transcripts (purple) in **E.** non-infected donors and in **F.** COVID19 patients. Spearman correlation coefficients were considered significant and are indicated with * when p < 0.05.

Infections involve complex cross-communication and cross-regulation among various blood cell components including platelets and circulating leukocytes. Whether platelet-PRRs are associated with leukocyte transcripts related to coagulation and thrombolysis, or cell-cell interactions, is unknown; similarly, it is not known whether leukocyte-PRRs associate with changes in the selected platelet transcripts. To assess the correlation between platelet- and leukocyte-PRRs and their cross-contribution to expression of thrombotic and coagulation transcripts in the other cell population, we conducted cross-correlation analyses of the two cell types’ specific immune receptors with infection. Our data show that in non-infected donors, platelet-MDA5 has the most positive correlations with leukocyte-PRRs, including TLR7, TLR9, RIG-I, MDA5, and LGP2 (Fig 5A and S11 Table). With SARS-CoV-2 infection, platelet-MDA5 continued to positively associate with leukocyte-TLR7, -RIG-I, -MDA5, and -LGP2. However, in addition to platelet-MDA5, -RIG-I, -LGP2, and -CGAS also became associated with leukocyte-RIG-I, -MDA5, and -LGP2 (Fig 5B and S12 Table). As MDA5, RIG-I, and LGP2 are interferon-stimulated genes, it is not unexpected that they change in a correlative manner with infection. We then wanted to address the correlations between platelet-PRRs and leukocyte transcripts related to coagulation and thrombolysis and to platelet-immune cell interactions. In non-infected donors, platelet-LGP2 associated with the greatest number of selected transcripts, including negatively associating with leukocyte-PLAU, and positively associating with leukocyte-F13A1, -CD40, and -CD40LG (Fig 5C and S13 Table). Similarly, in non-infected donors, the leukocyte transcripts that associated with the most platelet-PRRs were leukocyte-F13A1 (platelet-TLR3, -MDA5 and -LGP2) and leukocyte-CD40LG (platelet-RIG-I, -MDA5, and -LGP2). With SARS-CoV-2 infection, platelet RIG-I, -MDA5, -LGP2, and -CGAS became correlated with leukocyte-SERPING1 and -CD40, with leukocyte-SERPING1 associating with the highest number of platelet-PRRs (Fig 5D and S14 Table).

Next, we aimed to assess the correlation of leukocyte-PRRs with platelet transcripts related to thrombosis, coagulation and platelet-leukocyte interactions. In non-infected donors, leukocyte-CGAS showed the most correlations with the selected platelet transcripts, including platelet-SERPING1, -GP6, -GP5, and -ITGA2B, with platelet-SERPING1 associating with the most leukocyte-PRRs: TLR5, TLR6, and CGAS (Fig 5E and S15 Table). With SARS-CoV-2 infection,leukocyte-RIG-I, -MDA5, and -LGP2 became correlated with platelet-GP6 and -GP9 (Fig 5F and S16 Table). In the COVID19 patients, the PRRs that correlated with the most thrombosis-coagulation related transcripts were MDA5, followed by RIG-I, and LGP2 (Fig 5F and S16 Table). Additionally, in COVID19 patients, platelet-SELP positively correlated with the highest number of leukocyte PRRs, including leukocyte-TLR2, -TLR3, -TLR4, -TLR6, -TLR8, -RIG-I and -CGAS (Fig 5F and S16 Table). These data suggest that PRR transcriptional changes in platelets cross-correlate with specific thrombotic-coagulation-thrombolysis-related transcripts in leukocytes, and those cross-correlation patterns change with infection.

### COVID19 clinical outcomes are associated with distinct PRRs in platelets versus leukocytes

Activation of certain PRRs, particularly in platelets, is associated with survival and broadly associated with immunothrombosis. Whether PRR expression in either platelets or in leukocytes can be correlated with COVID19 outcomes, coagulopathy, or other clinically relevant outcomes is unknown. Here, we aimed to determine whether changes in platelet- or leukocyte-PRRs are associated with clinical outcomes of COVID19 patients. To address this question, we correlated platelet- and leukocyte-PRRs with clinical characteristics assessed in COVID19 patients at the time of blood collection. Severity of infection measured by the 8-category ordinal scale (OS), where 1 is not hospitalized and 8 is death [31], positively correlated with platelet-TLR3 (Table 1) and leukocyte-TLR5 (Table 2), while leukocyte-TLR7 (Table 2) showed an inverse correlation. This indicates that platelet-TLR3 (senses replicating double-stranded vRNA) and leukocyte-TLR5 (senses bacterial flagellin) can be associated with greater severity of COVID19 whereas increased expression of leukocyte-TLR7 correlates with low severity. With respect to coagulability, we correlated PRRs with D-dimer and INR (international normalized ratio). INR measures clotting tendency, where high INR-indicates propensity for bleeding and low INR-for clotting [32]. D-dimer did not associate with any of the PRRs in either cell population, while INR associated inversely with platelet-TLR4 and -TLR10, and leukocyte-TLR10. This proposes that increased levels of platelet-TLR4 and leukocyte-TLR10 are associated with higher propensity for clotting (Tables 1 and 2). Elevated levels of the liver enzymes alanine transaminase (ALT) and aspartate aminotransferase (AST), indicating liver inflammation and/or damage [33], were also correlated with PRRs in platelets and leukocytes. Expression of platelet- and leukocyte-LGP2 in addition to leukocyte-TLR3, -TLR6, -TLR7 and -RIG-I associated with ALT (Tables 1 and 2). On the other hand, platelet-TLR6, -TLR9, -CGAS, and leukocyte-RIG-I, -MDA5, and -LGP2 positively correlated with AST (Tables 1 and 2). These data propose that liver inflammation is closely associated with increased levels of PRR transcripts in both platelets and leukocytes, where the PRRs apart from LGP2 are distinct for each blood component. Interestingly, correlations between PRRs and platelet, leukocyte, or erythrocyte counts in blood were not observed (Tables 1 and 2). Overall, our data indicate that in COVID19 patients, severity of infection, coagulability potential, and liver inflammation associate mostly with distinct PPRs in platelets versus leukocytes proposing that patterns of PRR changes during infection may be more informative than changes in the levels of one receptor.

**Table 1:** Platelet-PRR expression and clinical outcomes in COVID19 patients.

	Ordinal Scale	Days of Symptoms	INR	D-dimer	Troponin	ALT	AST	Platelet Count	WBC Count	RBC Count
n-number of pairs	10	8	8	7	10	9	9	10	9	10
**TLR1**	0.27	-0.55	-0.18	0.64	-0.36	0.18	0.02	-0.37	0.30	0.56
	0.46	0.16	0.67	0.14	0.31	0.64	0.98	0.30	0.44	0.10
**TLR2**	0.06	0.11	-0.25	0.14	0.24	0.07	0.27	-0.12	0.33	-0.37
	0.88	0.81	0.54	0.78	0.51	0.88	0.49	0.76	0.39	0.30
**TLR3**	**0.82**	-0.21	0.54	0.31	0.39	-0.24	0.49	0.16	-0.02	0.06
	**0.01**	0.62	0.17	0.50	0.26	0.52	0.18	0.65	0.98	0.88
**TLR4**	-0.47	0.25	**-0.81**	-0.07	-0.09	0.33	0.08	-0.16	0.37	-0.08
	0.17	0.55	**0.02**	0.91	0.81	0.39	0.84	0.66	0.34	0.84
**TLR5**	-0.30	-0.48	-0.06	0.21	0.43	0.29	0.08	0.10	0.14	-0.40
	0.39	0.23	0.90	0.66	0.22	0.44	0.83	0.79	0.72	0.25
**TLR6**	-0.09	-0.20	0.17	-0.02	0.41	0.31	**0.70**	0.16	-0.17	-0.41
	0.81	0.65	0.68	0.99	0.24	0.43	**0.05**	0.65	0.67	0.23
**TLR7**	-0.03	-0.18	-0.58	0.11	-0.36	0.28	0.28	-0.42	0.05	0.52
	0.94	0.67	0.14	0.84	0.31	0.46	0.46	0.23	0.91	0.13
**TLR8**	0.33	0.06	-0.41	0.31	-0.11	0.02	0.13	-0.24	0.53	0.35
	0.35	0.90	0.31	0.50	0.76	0.97	0.73	0.51	0.15	0.32
**TLR9**	0.30	-0.55	0.48	-0.07	0.51	0.37	**0.85**	0.08	-0.43	-0.33
	0.39	0.16	0.23	0.91	0.14	0.34	**0.01**	0.84	0.25	0.35
**TLR10**	-0.43	0.49	**-0.77**	-0.40	-0.45	-0.02	-0.25	-0.44	0.23	-0.07
	0.23	0.22	**0.05**	0.43	0.21	0.98	0.52	0.21	0.56	0.87
**RIG-I**	-0.13	-0.60	-0.23	0.07	-0.24	0.57	0.60	-0.29	-0.20	0.39
	0.72	0.12	0.58	0.91	0.51	0.12	0.10	0.43	0.61	0.26
**MDA5**	-0.12	-0.41	-0.21	-0.04	-0.17	0.47	0.65	-0.25	-0.38	0.31
	0.74	0.32	0.62	0.96	0.65	0.21	0.07	0.49	0.31	0.39
**LGP2**	-0.19	-0.36	-0.46	0.36	-0.28	**0.75**	0.57	-0.15	-0.03	0.51
	0.60	0.38	0.26	0.44	0.42	**0.03**	0.12	0.67	0.96	0.14
**CGAS**	0.30	-0.66	0.28	0.00	0.37	0.27	**0.77**	-0.07	-0.37	0.07
	0.39	0.08	0.50	1.00	0.29	0.49	**0.02**	0.87	0.34	0.87

*Correlations were assessed by Spearman R (top value) and statistical significance (p < 0.05, bottom value) are indicated in blue. Abbreviations are as follows: TLR: Toll-like receptor, RIG-I: DDX58-RNA sensor RIG-I, MDA5: Melanoma differentiation-associated protein 5, LGP2: DHX58-DExH-box helicase 58, CGAS: Cyclic GMP-AMP synthase. INR: International Normalized Ratio used to measure blood coagulation, ALT: Alanine Transaminase, AST: Aminotransferase, WBC: White Blood Cells, RBC: Red Blood Cells. Ordinary score here measures severity of infection, as 1-indicates no hospitalization and 8 indicates death.

**Table 2:** Leukocyte-PRR expression and clinical outcomes in COVID19 patients.

	Ordinal Scale	Days of Symptoms	INR	D-dimer	Troponin	ALT	AST	Platelet Count	WBC Count	RBC Count
n-number of pairs	10	8	8	7	10	9	9	10	9	10
**TLR1**	-0.35	-0.72	0.27	0.04	0.09	0.32	-0.33	-0.14	-0.28	-0.20
	0.32	0.05	0.52	0.96	0.84	0.41	0.95	0.71	0.16	0.58
**TLR2**	0.20	0.06	-0.37	0.71	0.07	0.40	0.22	0.02	0.52	0.14
	0.57	0.90	0.36	0.09	0.86	0.29	0.58	0.97	0.16	0.71
**TLR3**	0.21	-0.56	-0.03	0.54	0.42	**0.78**	0.68	0.39	0.00	0.26
	0.56	0.15	0.97	0.24	0.23	**0.02**	0.05	0.26	1.00	0.47
**TLR4**	0.51	0.08	0.08	0.43	0.50	0.23	0.68	0.24	0.30	0.07
	0.13	0.85	0.85	0.35	0.15	0.55	0.05	0.51	0.44	0.87
**TLR5**	**0.85**	0.23	0.19	0.32	0.24	-0.50	0.23	0.09	0.38	0.26
	**0.01**	0.59	0.65	0.50	0.51	0.91	0.55	0.81	0.31	0.47
**TLR6**	-0.19	-0.29	-0.24	0.71	0.13	**0.73**	0.30	0.55	0.48	0.27
	0.61	0.49	0.56	0.09	0.73	**0.03**	0.44	0.10	0.19	0.45
**TLR7**	**-0.74**	-0.47	-0.41	-0.39	-0.10	**0.73**	0.43	-0.20	-0.52	-0.14
	**0.02**	0.25	0.31	0.40	0.78	**0.03**	0.25	0.58	0.16	0.71
**TLR8**	0.13	-0.12	-0.16	0.64	0.55	0.53	0.42	0.59	0.60	0.03
	0.72	0.78	0.71	0.14	0.11	0.15	0.27	0.08	0.10	0.95
**TLR9**	0.43	-0.61	0.35	0.32	-0.03	0.12	0.48	-0.25	-0.08	0.46
	0.21	0.12	0.39	0.50	0.94	0.78	0.19	0.49	0.84	0.19
**TLR10**	-0.62	0.31	**-0.82**	0.20	-0.25	0.29	-0.42	0.15	0.53	0.19
	^0.06^	0.44	**0.02**	0.67	0.48	0.44	0.25	0.69	0.15	0.60
**RIG-I**	-0.11	-0.46	-0.12	-0.14	0.17	**0.73**	**0.85**	0.06	-0.25	0.29
	0.77	0.26	0.78	0.78	0.65	**0.03**	**0.01**	0.89	0.52	0.43
**MDA5**	-0.02	-0.72	0.06	-0.04	0.01	0.53	**0.75**	-0.19	-0.45	0.25
	0.96	0.05	0.89	0.96	1.00	0.15	**0.03**	0.16	0.23	0.49
**LGP2**	-0.19	-0.58	-0.01	-0.11	0.09	**0.75**	**0.83**	-0.02	-0.35	0.21
	0.59	0.14	0.83	0.84	0.81	**0.03**	**0.01**	0.97	0.36	0.56
**CGAS**	0.09	-0.62	0.48	0.54	0.17	0.38	0.48	0.15	-0.15	0.31
	0.81	0.10	0.23	0.24	0.65	0.31	0.19	0.68	0.71	0.39

*Correlations were assessed by Spearman R (top value) and statistical significance (p < 0.05, bottom value) are indicated in blue. Abbreviations are as follows: TLR: Toll-like receptor, RIG-I: DDX58-RNA sensor RIG-I, MDA5: Melanoma differentiation-associated protein 5, LGP2: DHX58-DExH-box helicase 58, CGAS: Cyclic GMP-AMP synthase. INR: International Normalized Ratio used to measure blood coagulation, ALT: Alanine Transaminase, AST: Aminotransferase, WBC: White Blood Cells, RBC: Red Blood Cells. Ordinal scale here measures severity of infection, as 1-indicates no hospitalization, no impairment and 8 indicates death.

### Whole blood PRR associations with thrombosis and coagulation transcripts are more similar between COVID19 and TB than between COVID19 and influenza

**Fig 6:**
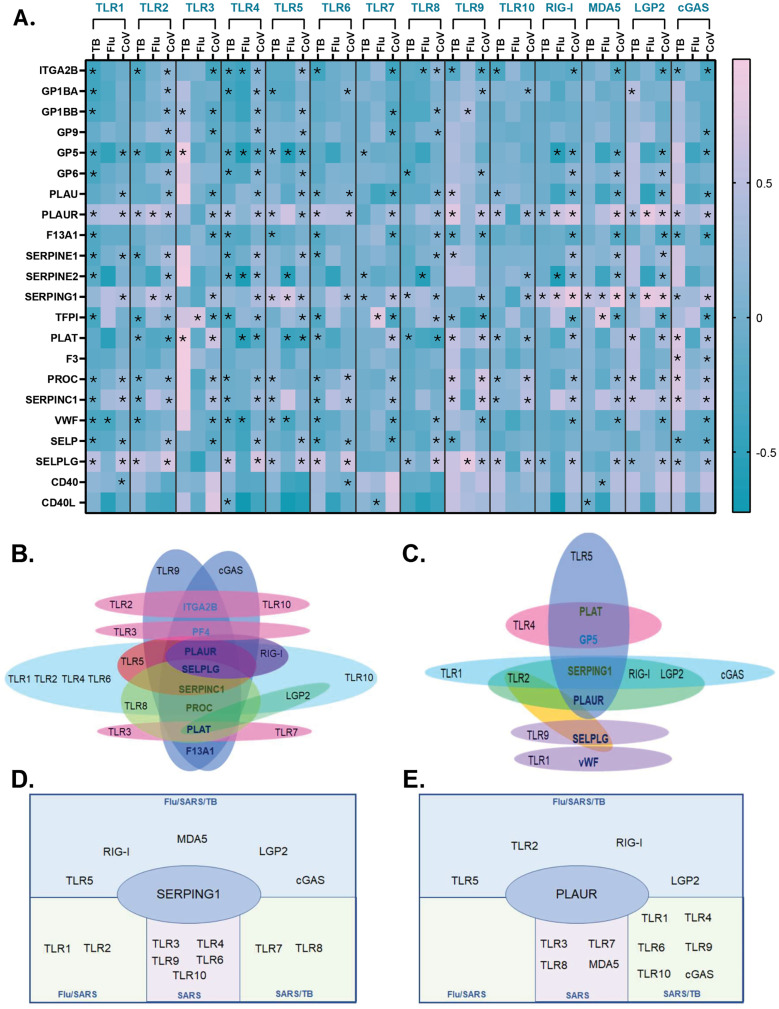
Comparative analysis of PRRs and select transcripts related to thrombosis, coagulation and platelet-leukocyte interactions across three respiratory infections, COVID19, influenza and tuberculosis (TB). Whole blood RNAseq data from publicly available databases was normalized to three housekeeping genes as described in the Methods. All data are from the acute state of infection. **A.** Heatmap of correlation coefficients between PRRs and prothrombotic/coagulation transcripts from TB, influenza, and COVID19 patient whole blood samples. For clarity, * indicates only statistically significant values of *p < 0.03. Venn-diagrams of transcript correlations between **B.** TB and COVID19 and **C.** influenza and COVID19. Correlations of **D.** SERPING1 and **E.** PLAUR. Spearman correlation coefficients were considered significant and are indicated with * when p < 0.05.

To determine whether the observed platelet and leukocyte PRR expression patterns and their association with thrombotic gene transcripts can be detected in whole blood and whether they differ across acute respiratory infections, we analyzed whole blood gene expression data from COVID19, TB, and influenza patients using three publicly available databases (see Methods). Similar to our platelet and leukocyte RNAseq analysis, we normalized each database participant to the same three housekeeping genes (see Methods). Correlations between PRRs and transcripts related to thrombosis and coagulation identified three transcripts having the highest associations with PRRs across all three infections: PLAUR, SERPING1, and SELPLG. The gene transcripts with the greatest number of shared correlations with PRRs across all three infections were PLAUR (with TLR2, RIG-I, LGP2) and SERPING1 (with TLR5, RIG-I, MDA5, and LGP2) (Fig 6A, and S17, S18, and S19 Tables). In all three infections, SELPLG was associated with TLR9 (Fig 6A and S17, S18, and S19 Tables). These associations were all positive across the three infections (Fig 6 and S17, S18, and S19 Tables).

Interestingly, when correlations in patients with TB were compared to COVID19 patients, multiple coagulation-related gene transcripts, PLAUR, SERPINC1, and PROC, demonstrated similar widespread association with numerous PRRs (Fig 6B). SELPLG, coding for the receptor for P-selectin, also showed similar broad correlation with PRRs in whole blood of infected patients (Fig 6B). Overall, associations in COVID19 and TB samples were generally positive and consistent in directionality (Fig 6A and S17, S18, and S19 Tables). The correlations between influenza and COVID19 patients had less similar associations between thrombotic- or coagulation-related transcripts with PRRs (Fig 6D). Finally, a few gene transcripts exhibited differential correlations with PRRs among all three respiratory infections. GP5 correlated negatively with RIG-I in COVID19 and influenza. Similarly, SERPINE2 correlated positively with TLR4 and RIG-I in COVID19 but negatively in influenza. TFPI correlated negatively with TLR3 and TLR7 in COVID19 but positively in influenza (Fig 6A and S17, S18, and S19 Tables). These data indicate that during the acute stage of respiratory infections, COVID19 has more overlapping associations of PRRs and thrombotic- or coagulation-related transcripts with TB than with influenza.

## Discussion

Platelets are the blood component primarily responsible for acute thrombotic responses and increased risk of thrombotic events during respiratory infections [1,7,17,34]. In addition to their well characterized function in hemostasis and thrombosis, platelets also mediate immunothrombotic interactions with the surrounding leukocytes through PRRs. This is reflected in heterotypic aggregation of various proportions which can also change with infection [6,35]. Respiratory viruses such as SARS-CoV-2 and influenza can cross over into the circulation and induce a response in platelets and in leukocytes [15,36]. These viruses do not generally cause viremia perhaps due to intricate interactions between the host’s PRR response inside of the cell, and the complement and coagulation responses outside of the cell [7]. Platelets have the ability to quickly internalize and neutralize these viruses [15,35,36], preventing them from reaching nucleated cells where they could potentially reproduce, but still alerting the immune system to danger. The consequence of this benefit to the host response comes with a price generally reflected as an increased risk of immunothrombotic outcomes characterized by dysregulated coagulation that often cannot be controlled by classical antiplatelet therapies [1]. In this study, we show that platelets and leukocytes express transcripts related to thrombosis, coagulation, and pathogen sensing at different levels when compared to each other. Not surprisingly, platelets contain overwhelmingly high levels of transcripts related to classical platelet-aggregation, adhesion, thrombosis, and coagulation while leukocytes have much higher levels of transcripts related to control of coagulation and thrombolysis. Leukocytes do express higher levels of PRRs, about 200-fold more on average; however, this difference reduces to 3-fold when the same analysis is performed in blood components isolated from COVID19 patients. Interestingly, the pattern of expression of PRRs differed between the two blood components; in leukocytes, the highest expressed PRR was TLR4, while in platelets, it was TLR9. Our findings also demonstrate that severity of infection and survival correlate with different PRRs between these two blood components; in leukocytes, TLR5 positively correlated and TLR7 negatively correlated with ordinal score, while in platelets, TLR3 was positively correlated. In this case, TLR7-response may be the compensatory outcome when the primary interferon response fails, evident by the lack of increase in interferon-stimulated genes (ISGs) such as MDA5 and RIG-I. Additionally, coagulopathy measured by INR, negatively correlated with TLR4 in platelets and TLR10 in leukocytes. Of note, INR also negatively correlated with TLR10 in platelets, but since TLR10 is not uniformly expressed in this blood component we have omitted claims about this receptor in platelets. TLR4 in platelets is known to mediate platelet-neutrophil interactions and decrease the time to NETosis [37], in addition to the fact, that it could participate in the thrombotic response in the presence of SARS-CoV-2 [21]. TLR10, contrary to the rest of the PRRs inhibits the proinflammatory Myd88 response, and thus is unique among the PRRs leading to negative regulation of inflammatory signaling by other PRRs [38]. TLR10 is not known to have a direct sensing potential for SARS-CoV-2 or outcome-mediated function in COVID19. Interestingly TLR10 is non-functional in mice highlighting the reduced translational impact when assessing coagulation-related outcomes of SARS-CoV-2 in humans. Overall, our data suggest that mounting a balanced platelet-leukocyte inflammatory response in addition to platelet-leukocyte interactions may be important for managing coagulopathy, and simple inhibition of one PRR at steady state versus the others may not be the proper solution for managing this outcome.

In the current study, we also investigated whether transcriptional programs related to thrombosis and coagulation in platelets and leukocytes change with respect to PRRs and whether those correlations are uniform in blood across three respiratory infections, COVID19, influenza and TB. We report that platelet and leukocyte PRR expression changes with SARS-CoV-2 infection, however PRR pattern and direction of change differs between the two blood populations. At baseline, in non-infected donors, leukocytes expressed PRRs at a much higher level than platelets, with the highest expressed leukocyte-PRR being TLR4, while in platelets it was TLR9. With infection, expression of certain PRRs in platelets increased (TLR9, RIG-I, and CGAS), while in leukocytes, they decreased (TLR6, TLR8). It is important to emphasize that platelets, as anucleated cell fragments, cannot synthesize new RNA. Therefore, observed changes in transcript levels reflect steady-state RNA present at the time of collection in platelets from both non-infected donors and COVID19 patients. With regard to transcript changes related to thrombotic receptors and thrombotic properties of platelets, only mild increases in F2RL3 (coding for the thrombin receptor PAR4), P2RY1 or P2RX1 were observed with infection, in addition to SELP (coding for P-selectin) which is the selectin primarily responsible for mediating platelet-leukocyte interactions. At baseline, in platelets, the transcript encoding for TFPI and the receptor for urokinase plasminogen activator (uPA) (PLAUR) positively correlated with the highest number of PRRs. With infection, in platelets, associations changed and expression of F2RL3 and GP6 both positively correlated with TLR6, TLR9, RIG-I, MDA5, LGP2, and CGAS.

Platelet-mediated thrombotic outcomes are also impacted by a dysregulated balance of procoagulant versus anticoagulant, or thrombolysis proteins in blood. Transcripts for those proteins are found to be present in both platelets and leukocytes [20]. No significant changes were observed for any of the procoagulant or anticoagulant transcripts in platelets with infection. In leukocytes, however, the anticoagulant SERPING1 (C1INH) increased while PLAUR (discussed below) decreased with COVID19. Correlations in expression programs with PRRs showed in platelets the thrombolysis-inhibiting SERPINE1 [39] (encoding for plasminogen activator inhibitor, PAI-1) positively correlated with TLR9 and MDA5 without infection, and during SARS-CoV-2 infection SERPINE1 correlated positively with MDA5 and RIG-I and negatively with TLR5. Anticoagulant SERPINE2 [40] (coding for Nexin) lost its positive correlations with TLR7 and RIG-I with COVID19. In leukocytes, expression of F13A1 [41] (forming coagulation FXIII) correlated with TLR7, TLR9, RIG-I, MDA5, and LGP2, and with infection the correlations changed to TLR6 and CGAS. The anticoagulant PROC [42] associated positively with TLR1 and TLR9, but, with infection, became inversely correlated with TLR2, TLR5, and TLR8. Our data indicate that the overall programs of transcript changes related to PRRs and control of coagulation differ between non-infected and COVID19 patients proposing that, during infection, PRRs should be looked at as a whole and not as one receptor that contributes to coagulopathy.

The final stage of a thrombotic event is clot resolution. Urokinase plasminogen activator (uPA coded by PLAU) and its receptor uPAR (PLAUR) are present in circulating blood cells. The uPA/uPAR system is responsible for plasmin generation [43] which, with respect to a thrombus, mediates thrombolysis and clot resolution [44]. On the other hand, neutrophil-uPAR can also be activated by FXII to regulate wound healing [45,46], a crucial process in recovery post pathogen-mediated inflammation and tissue damage. Platelets contain the transcript only for PLAUR, while leukocytes express both PLAUR and PLAU [20]. Here we report that infection does not change levels of PLAUR in platelets, while in leukocytes PLAU increased and PLAUR decreased. In platelets, PLAUR was positively associated with TLR2, TLR4, TLR8, and RIG-I, and with infection these associations changed to TLR7 and LGP2 but continued to correlate with TLR4 and TLR8. In leukocytes, PLAUR positively correlated with RIG-I and MDA5 which changed to TLR3, TLR6, and TLR8 with infection; PLAU, on the other hand, lost its correlation with TLR4 and TLR5 with COVID19. These observations suggest that, with infection, programs of PRR expression change concomitantly with programs related to thrombosis and coagulation proposing the potential of PRRs to influence thrombotic balance not only from a platelet perspective but also from leukocytes.

PRRs are also responsible for interactions between platelets and leukocytes during infection or with activation [7,17,47]. We have shown that TLR7 increases platelet-neutrophil heterotypic aggregates through P-selectin (SELP) on platelets and PSGL1 (SELPLG) on leukocytes similarly to CD40LG on platelets and CD40 on leukocytes [35]. In this small cohort, we only detected positive correlations between platelet-TLR9 and SELP, which was lost with infection. SELPLG in leukocytes also lost most of its PRR correlations with infection. The same was observed for platelet-CD40LG and leukocyte-CD40. A possible explanation for this loss is the HAGs between platelets and leukocytes that we report here, and others have reported before, with neutrophils and monocytes, in addition to platelet-eosinophil HAGs that have not been reported before. Additionally, there is a possibility that these platelets and leukocytes are exhibiting some form of cell death, as previously reported and as we show here by EM, and thus the transcripts for these proteins become undetectable. Finally, it is also possible that these particular populations with infection, in the form of microaggregates, become stuck in the microvasculature of lungs and contribute to the pathology of initial COVID19, TB, and in certain cases, influenza [48–51].

This is the first study to examine expression profiles of PRRs and transcripts related to thrombosis and coagulation in platelets and leukocytes isolated from the same donors and compared with and without infection. Our study does, however, have limitations. RNA-sequencing was performed on only a relatively small number of patients and control donors. The ability to control for leukocytes and platelets in the same individuals, however, is greatly beneficial to assess the overall comparison of expression profile between these two blood components. The objective here was to compare platelets versus all blood-leukocytes. Additionally, in-depth studies are necessary to assess the changes in correlations and transcriptional profiles in the different leukocyte populations. Overall, when it comes to broader answers for transcriptional programs related to thrombotic outcomes and the possible contribution of platelets versus leukocytes to the thrombotic process, our study provides useful data directing the basis for further focused investigations. One limitation to our isolation methods is that, although platelets isolated in this case are without detectable leukocytes (as we have shown with lncRNA analysis [15]), we cannot claim that the leukocyte population is free from platelets. In fact, here we provide evidence that platelets are present with, and platelet-derived microparticles are present in, leukocytes from infected individuals. Thus, certain transcripts such as GP1BA (not expressed in leukocytes) [52] are detected as a result of platelet presence, either in the form of microparticles or platelet-leukocyte aggregates. Methods such as CD41-antibody depletion for a pure leukocyte fraction would further activate the already immune-challenged populations. Finally, our isolation methods also prevented further correlation analysis linking platelet-leukocyte aggregates to COVID19 outcomes, as it is impossible to separate these interactions from internalized platelet-derived microparticles within leukocytes. Regardless of the limitations, our data demonstrate the associations between PRRs in both platelets and leukocytes with thrombotic, procoagulant, anticoagulant, and thrombolysis programs. We propose that both blood components contribute to the dysregulated thrombotic outcomes during infection that are beyond the control of antiplatelet therapies. Future studies are necessary to assess the overall protein levels and the contribution of each PRR in each component during the course of infection with respect to overall thrombosis, coagulation and thrombolysis.

Our data indicate that COVID19 and TB exhibit numerous shared associations between PRRs and gene transcripts related to thrombosis and coagulation. Surprisingly, fewer shared correlations were observed between influenza and COVID19 than were shared between COVID19 and TB. The basis for the greater similarity between COVID19 and TB is unclear but the shared propensity for chronic lung injury with these two infections, which is greater than typically seen with influenza, might in part be a consequence of dysregulated thrombosis and coagulation. Common associations were observed across all three infections which most frequently involved PLAUR and SERPING1, suggesting that common pathways linking acute respiratory infection to thrombosis may involve these gene transcripts. Overall, what is evident from our study is that there are distinct PRRs that correlate with specific patterns of transcripts related to thrombosis and coagulation, that depend on the blood component and on infection status. This is further supported by the observation that increased levels of TLR3 in platelets and TLR5 in leukocytes correlate with severity of infection and survival, while increased levels of leukocyte TLR7 are associated with reduced severity of infection. Future mechanistic studies are necessary to assess the direct impact of these PRR patterns in coagulopathy, severity of infection and survival.

## Methods

### Ethics statement

All procedures were approved by the University of Massachusetts Chan Institutional Review Board (IRB) (protocol # H00009277, 14268–10, H00017901) and participants signed informed consent as required by the IRB.

#### Blood collection and patient characteristics.

Blood from non-infected donors and COVID19 patients who tested positive for SARS-CoV-2 by RT-qPCR (α-δ strains; characteristics in S1 Table) was collected into acid citrate dextrose (ACD) tubes by phlebotomy between April and November 2020.

#### Platelet isolation.

Platelets were isolated from ACD-venous blood by the double centrifugation method as we have previously done [15]. Platelet number was measured via a blood cell analyzer (Beckman Coulter Ac.T8, CA, USA). Contamination of the platelet preparation was found to be < 1 in 50,000.

#### Leukocyte isolation.

Blood was centrifuged at 150 x g for 17 min. Platelet-rich plasma was removed and the bottom red blood cell fraction was lysed with 5 mL of RBC Lysis Buffer (Roche, cat#11814389001) as we have previously done [15]. Briefly, the samples were centrifuged at 350 x g for 5 min, low brake, and the pellet was washed with 10 mL of 1x PBS at the same conditions. The pellet was resuspended in 1 mL of 1x PBS, lysed once again with 1 mL of RBC Lysis Buffer, washed with 1x PBS, and centrifuged at 250 x g for 5 min, low brake. The pellet was saved in QIAzol (Qiagen, cat#79306) for sequencing.

#### RNA isolation and RNAseq.

Isolated platelets or leukocytes were lysed in 700 µL QIAzol and frozen at -80°C. RNA isolation was performed as we have previously done [53] using the miRNeasy Mini or Micro Kit (Qiagen, cat#217004, cat#1071023), according to the manufacturer’s instructions. DNA was eliminated using on-column DNA digestion with the RNase-Free DNase Set (Qiagen, cat#79254). Libraries from the RNA samples were created by using the AmpliSeq Transcriptome Human Gene Expression Kit (ThermoFisher, cat#A26327) according to the manufacturer’s protocol [15]. The AmpliSeq human transcriptome gene expression primer pool targets 18,574 protein-coding messenger RNAs. Sequencing was executed using an Ion Proton sequencer (ThermoFisher), with Ion PI Hi-Q sequencing chemistry. Our analysis was focused on PRRs and transcripts related to thrombosis, coagulation, thrombolysis and platelet-mediated immune interactions with leukocytes (S2 Table).

#### COVID19, influenza, and TB whole blood databases.

A secondary analysis of multiple publicly available clinical databases containing whole blood raw RNAseq data of patients acutely infected with TB, SARS-CoV-2, or influenza was performed. Briefly, the Molecular Signatures of Tuberculosis-Diabetes Interaction cohort was originally studied in a comparison of baseline and longitudinal blood gene expression among patients infected with tuberculosis from South India and Brazil [54]. Whole blood RNA was collected at baseline, as well as months 2 and 6 after initiating anti-TB treatment (raw data publicly available at https://www.ncbi.nlm.nih.gov/geo/query/acc.cgi?acc=GSE181143) [54]. The PREDICT-19 cohort was utilized to compare the gene expression profiles of patients with either confirmed SARS-CoV-2 infection or healthy controls. Whole blood RNAseq data was collected in patients from Australia (Sidney, Melbourne, Perth) and the Czech Republic (raw data publicly available at https://www.ncbi.nlm.nih.gov/geo/query/acc.cgi?acc=GSE217948) [55]. We included whole blood RNAseq data collected through the Duke University Health System from patients who presented to the Emergency Department with confirmed influenza infection (raw data publicly available at https://www.ncbi.nlm.nih.gov/geo/query/acc.cgi?acc=GSE161731) [56]. Detailed study designs have been provided in previous publications [57–62]. Blood samples from all three cohorts were collected in PAXgene blood RNA tubes.

#### Statistical analysis.

In our analysis of platelets and leukocytes of SARS-CoV-2-infected patients, mean expression levels (in BaseMean) of PRRs and the rest of the transcripts used in the study were normalized (see below) before any analysis. Similar normalization was performed for whole blood RNAseq analyses from the three-databases used. Gene expression levels for each donor were normalized using the geometric mean of the BaseMean of three housekeeping genes: beta actin (ACTB), beta-2-microglobuin (B2M), and glyceraldehyde-3-phosphate dehydrogenase (GADPH), as follows: Expression(geneX)=(Average((geneX/ACTB)+(geneX/B2M)+(geneX/GAPDH)))*10,000. This normalization allowed us to compare expression across different blood components. The three housekeeping genes were selected based on previous epidemiological studies of quantitative gene expression in peripheral blood generated by the Framingham Heart Study [20]. Of note, in platelets, levels of TLR10 were very low and only detected in 13% of the donors (S3 Table), thus we have excluded TLR10 from further analysis. Two-tailed p values <0.05 were deemed statistically significant. Correlation coefficients were calculated to compare co-expression levels between PRRs and, broadly, thrombosis-related transcripts and other genes (S2 Table). Statistical analysis was performed using R v4.3.3 statistical software (R Foundation for Statistical Computing, Vienna, Austria) or GraphPad Prism, version 10.1.2.

## Supporting information

S1 Data Sequencing Data in ReadCounts of platelets and leukocytes, from the same noninfected donors or patients, and measured outcomes in COVID19 patients.
(XLSX)

S1 Fig Pan leukocyte fraction shows platelets interacting with leukocytes. Transmission electron microscopy of isolated pan leukocytes shows different leukocyte populations, some of which are dying and are surrounded by other leukocytes and platelets. Images are representative of n = 4 COVID19 patients.
(TIF)

S1 Table Characteristics of patients used in the platelet sequencing analysis and throughout this study. (DOCX)

S2 Table Thrombosis-coagulation-thrombolysis and immunity-related transcripts that were used in this study. (DOCX)

S3 Table Correlation and significance in expression between pathogen-associated molecular pattern receptors among platelets of non-infected donors. (n = 15) *Heatmap for* Fig 1E.
(DOCX)

S4 Table Correlation and significance in expression between pathogen-associated molecular pattern receptors among platelets of COVID19 patients. (n = 10) *Heatmap for* Fig 1F.
(DOCX)

S5 Table Correlations in expression between pathogen-associated molecular pattern receptors among leukocytes of non-infected donors. (n = 15) *Heatmap for* Fig 1G.
(DOCX)

S6 Table Correlation and significance in expression between pathogen-associated molecular pattern receptors among leukocytes of COVID19 patients. (n = 10) *Heatmap for* Fig 1H.
(DOCX)

S7 Table Correlation and significance in expression between pathogen-associated molecular pattern receptors among platelets of non-infected donors. (n = 15) *Heatmap for* Fig 2A.
(DOCX)

S8 Table Correlation and significance in expression between pathogen-associated molecular pattern receptors and prothrombotic or coagulation-associated gene transcripts among platelets of COVID19 patients. (n = 10) *Heatmap for* Fig 2B.
(DOCX)

S9 Table Correlations in expression between pathogen-associated molecular pattern receptors and thrombolytic or cell-cell interaction-associated gene transcripts among leukocytes of non-infected donors. (n = 15) *Heatmap for* Fig 2D.
(DOCX)

S10 Table Correlations in expression between pathogen-associated molecular pattern receptors and thrombolytic and cell-cell interaction-associated gene transcripts among leukocytes of COVID19 patients. (n = 10) *Heatmap for* Fig 2E.
(DOCX)

S11 Table Correlation and significance in expression between pathogen-associated molecular pattern receptors in platelets (purple) and leukocytes (light orange) in platelets from non-infected donors. (n = 15) *Heatmap for* Fig 5A.
(DOCX)

S12 Table Correlation and significance in expression between pathogen-associated molecular pattern receptors in platelets (purple) and leukocytes (light orange) in platelets from COVID19 patients. (n = 10) *Heatmap for* Fig 5B.
(DOCX)

S13 Table Correlation and significance in expression between pathogen-associated molecular pattern receptors in platelets (purple) and coagulation or platelet-leukocyte interaction related transcripts in leukocytes (light orange) in platelets from non-infected donors. (n = 15) *Heatmap for* Fig 5C.
(DOCX)

S14 Table Correlation and significance in expression between pathogen-associated molecular pattern receptors in platelets (purple) and coagulation or platelet-leukocyte interaction related transcripts in leukocytes (light orange) in platelets from COVID19 patients. (n = 10) *Heatmap for* Fig 5D.
(DOCX)

S15 Table Correlation and significance in expression between pathogen-associated molecular pattern receptors in leukocytes (light orange) and thrombosis-coagulation related transcripts in platelets (purple) from non-infected donors. (n = 15) *Heatmap for* Fig 5E.
(DOCX)

S16 Table Correlation and significance in expression between pathogen-associated molecular pattern receptors in leukocytes (light orange) and thrombosis-coagulation related transcripts in platelets (purple) from COVID19 patients. (n = 10) *Heatmap for* Fig 5F.
(DOCX)

S17 Table Whole blood correlations in expression between Toll-like receptor or retinoic acid-inducible gene I receptor and prothrombotic or coagulation-related gene transcripts among COVID19 patients. (n = 334).
(DOCX)

S18 Table Whole blood correlations in expression between Toll-like receptor or retinoic acid-inducible gene I receptor and prothrombotic or coagulation-related gene transcripts among influenza patients. (n = 17).
(DOCX)

S19 Table Whole blood correlations in expression between Toll-like receptor or retinoic acid-inducible gene I receptor and prothrombotic or coagulation-related gene transcripts among tuberculosis patients. (n = 118).
(DOCX)
