# Supplementary figures and images for: Pattern recognition receptor-associated immuno-thrombotic transcript changes in platelets and leukocytes with COVID19
Source: PLoS Pathog. 2025 Aug 18;21(8):e1013413. doi: 10.1371/journal.ppat.1013413 (PMC12373281; doi:10.1371/journal.ppat.1013413)

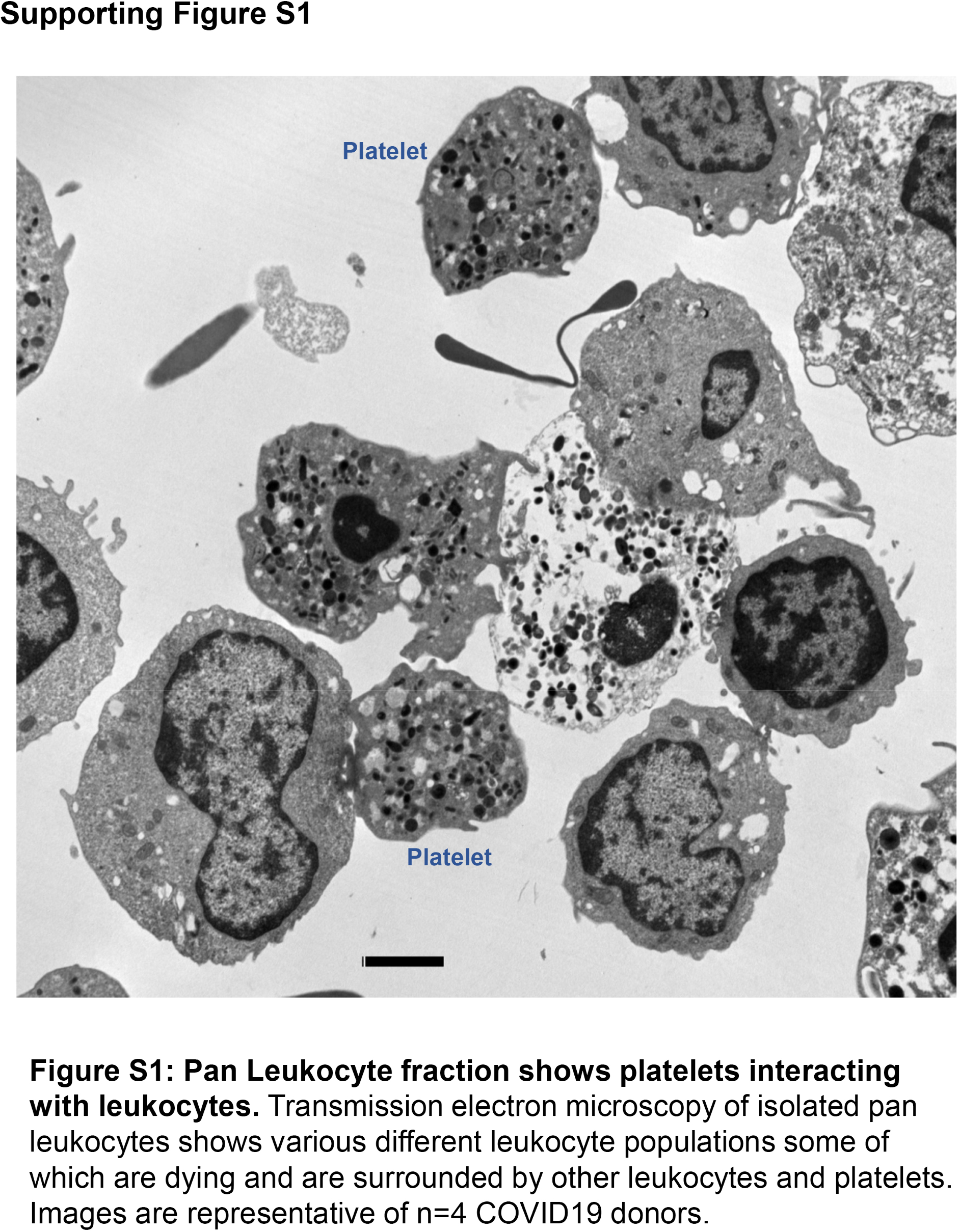

Supplement: S1 Fig — Transmission electron microscopy of isolated pan leukocytes shows different leukocyte populations, some of which are dying and are surrounded by other leukocytes and platelets. Images are representative of n = 4 COVID19 patients. (TIF) [file ppat.1013413.s002.tif]
