# Supplementary material for: Pattern recognition receptor-associated immuno-thrombotic transcript changes in platelets and leukocytes with COVID19
Source: PLoS Pathog. 2025 Aug 18;21(8):e1013413. doi: 10.1371/journal.ppat.1013413 (PMC12373281; doi:10.1371/journal.ppat.1013413)
Supplement: S1 Table — (DOCX) [file ppat.1013413.s003.docx]

**Table S1:** Characteristics of patients used in the platelet sequencing analysis and throughout this study.

| **Variable** | **Non-infected** | **COVID19** |
| --- | --- | --- |
| **n** | 15 | 10 |
| **Age** | 63.9 ± 12 | 63.2 ± 12 |
| **Sex (F%)** | 30% | 30% |
| **Race/Ethnicity** | Asian 1 | Asian 2 |
|  | Black 0 | Black 1 |
|  | Hispanic 2 | Hispanic 4 |
|  | White 12 | White 3 |
| **Time post COVID-19 diagnosis (days)** | -- | 5.5 ± 6 |
| **Aspirin (%)** | 100% | 30% |
| **ALT** | 85.6 ± 73 | 52.8 ± 35 |
| **AST** | 74.3 ± 80 | 45.7 ± 5 |
| **RBC (10^6^/µl)** | 3.6 ± 1 | 3.6 ± 1 |
| **Leukocytes (10^3^/µl)** | 8.3 ± 1 | 6.7 ± 1 |
| **Platelets (10^3^/µl)** | 221 ± 113 | 260 ± 25 |
| **Troponin** | 3.9 ± 4 (n=12) | 0.44 ± 0.4 (n=9) |
|  | >70 (n=3); | >70 (n=1); |

*Values are shown as mean ± SD; AST-; ALT-; RBC- red blood cells; There are no significant changes between any of the characteristics assessed by Mann-Whitney t-test.
