# Supplementary material for: Pattern recognition receptor-associated immuno-thrombotic transcript changes in platelets and leukocytes with COVID19
Source: PLoS Pathog. 2025 Aug 18;21(8):e1013413. doi: 10.1371/journal.ppat.1013413 (PMC12373281; doi:10.1371/journal.ppat.1013413)
Supplement: S3 Table — (n = 15) Heatmap for Fig 1E. (DOCX) [file ppat.1013413.s005.docx]

**Table S2**: Thrombosis-coagulation-thrombolysis and immunity-related transcripts that were used in this study.

| **Transcript** | **Protein** | **Process** | **Platelets vs Leukocyte expression** |
| --- | --- | --- | --- |
| **CD40** | Receptor for CD40LG/CD154 | platelet-leukocyte interaction | Both (Leuko-predominant) |
| **CD40LG** | Ligand for Cd40 expressed on plts | platelet-leukocyte interaction | Both |
| **F13A1** | A1 subunit of Coagulation factor XIII | coagulation | Both |
| **F2R** | PAR1 receptor for thrombin (FIIa) | thrombosis and coagulation | Both (Plt-predominant) |
| **F2RL3** | PAR4 receptor for thrombin (FIIa) | thrombosis and coagulation | Platelets |
| **F3** | Tissue factor (FIII) | thrombosis and coagulation | Leukocytes |
| **GP1BA** | Platelet vWF receptor | thrombosis and coagulation | Platelets |
| **GP5** | Platelet vWF receptor | thrombosis and coagulation | Platelets |
| **GP6** | Platelet collagen receptor | thrombosis and coagulation, immunity related | Platelets |
| **GP9** | Platelet vWF receptor | thrombosis and coagulation | Platelets |
| **ITGA2B** | Fibrinogen receptor | thrombosis and coagulation | Platelets |
| **P2RX1** | ATP receptor/channel | platelet shape change | Both |
| **P2RY1** | ADP receptor | thrombosis | Platelets |
| **P2RY12** | ADP receptor | thrombosis | Platelets |
| **PLAU** | Urokinase, plasminogen activator | thrombolysis | Leukocytes |
| **PLAUR** | Urokinase receptor | thrombolysis; angiogenesis | Platelets and Leukocytes |
| **PROC** | Protein C, Inactivator Of Coagulation Factors Va And VIIIa | inhibits coagulation and protective of endothelial damage | Leukocytes |
| **SELP** | Platelet-selectin | platelet-leukocyte interactions, immunity related | Platelets |
| **SELPLG** | P-selectin ligand | platelet-leukocyte interactions, immunity related | Leukocytes and Platelets |
| **SERPINC1** | Antithrombin III | inhibits coagulation | Leukoctyes |
| **SERPINE1** | Plasminogen Activator Inhibitor 1 | thrombolysis | Platelets and Leukocytes |
| **SERPINE2** | Nexin | inhibits coagulation and thrombosis | Platelets and Leukocytes |
| **SERPING1** | Compliment 1 inhibitor | inhibits coagulation and compliment cascade | Platelets and Leukocytes |
| **TBXA2R** | Thromboxane A2 receptor | platelet activation and thrombosis | Platelets |
| **TFPI** | Tissue factor pathway inhibitor | inhibits coagulation and thrombosis | Leukocytes |
| **VWF** | Von Willebrand Factor | coagulation and platelet adhesion | Platelets |
