# Supplementary material for: Pattern recognition receptor-associated immuno-thrombotic transcript changes in platelets and leukocytes with COVID19
Source: PLoS Pathog. 2025 Aug 18;21(8):e1013413. doi: 10.1371/journal.ppat.1013413 (PMC12373281; doi:10.1371/journal.ppat.1013413)
Supplement: S4 Table — (n = 10) Heatmap for Fig 1F. (DOCX) [file ppat.1013413.s006.docx]

**Table S3**: Correlation and significance in expression between pathogen-associated molecular pattern receptors among platelets of non-infected donors. (n=15) *Heatmap for Fig. 1EE*

| **TLR1** | **TLR2** | **TLR3** | **TLR4** | **TLR5** | **TLR6** | **TLR7** | **TLR8** | **TLR9** | **TLR10** | **RIG-I** | **MDA5** | **LGP2** | **cGAS** |
| --- | --- | --- | --- | --- | --- | --- | --- | --- | --- | --- | --- | --- | --- |
|  |  |  |  |  |  |  |  |  |  |  |  |  |  |

| Non-Infected  (% expressed) | 80 | 80 | 20 | 100 | 40 | 53 | 67 | 93 | 100 | 13 | 100 | 100 | 60 | 100 |
| --- | --- | --- | --- | --- | --- | --- | --- | --- | --- | --- | --- | --- | --- | --- |
| **TLR1** | 1.00 | 0.27 | 0.14 | -0.20 | 0.38 | 0.32 | 0.14 | -0.05 | 0.16 | --- | -0.18 | 0.06 | 0.09 | 0.25 |
|  | 0 | 0.33 | 0.61 | 0.47 | 0.16 | 0.24 | 0.61 | 0.87 | 0.57 |  | 0.53 | 0.84 | 0.74 | 0.37 |
| **TLR2** | 0.27 | 1.00 | 0.12 | **0.63** | **0.53** | 0.25 | 0.09 | **0.66** | -0.29 | --- | 0.33 | -0.02 | -0.07 | -0.51 |
|  | 0.33 | 0 | 0.68 | **0.01** | **0.05** | 0.37 | 0.74 | **0.01** | 0.30 |  | 0.23 | 0.94 | 0.80 | 0.06 |
| **TLR3** | 0.14 | 0.12 | 1.00 | -0.30 | -0.14 | 0.08 | -0.14 | 0.16 | 0.09 | --- | 0.22 | 0.26 | 0.22 | -0.39 |
|  | 0.61 | 0.68 | 0 | 0.24 | 0.59 | 0.78 | 0.62 | 0.57 | 0.75 |  | 0.43 | 0.35 | 0.43 | 0.15 |
| **TLR4** | -0.20 | **0.63** | -0.30 | 1.00 | 0.34 | 0.23 | 0.33 | 0.37 | -0.38 | --- | 0.41 | -0.29 | -0.15 | -0.42 |
|  | 0.47 | **0.01** | 0.27 | 0 | 0.21 | 0.42 | 0.23 | 0.17 | 0.16 |  | 0.13 | 0.29 | 0.59 | 0.12 |
| **TLR5** | 0.38 | **0.53** | -0.14 | 0.34 | 1.00 | -0.04 | 0.22 | 0.31 | -0.10 | --- | 0.07 | -0.10 | -0.21 | -0.08 |
|  | 0.16 | **0.05** | 0.59 | 0.21 | 0 | 0.90 | 0.43 | 0.25 | 0.71 |  | 0.80 | 0.71 | 0.45 | 0.78 |
| **TLR6** | 0.32 | 0.25 | 0.08 | 0.23 | -0.04 | 1.00 | **0.72** | 0.07 | -0.17 | --- | 0.46 | 0.50 | 0.30 | 0.04 |
|  | 0.24 | 0.37 | 0.78 | 0.42 | 0.90 | 0 | **4.2e-3** | 0.80 | 0.55 |  | 0.09 | 0.06 | 0.27 | 0.90 |
| **TLR7** | 0.14 | 0.09 | -0.14 | 0.33 | 0.22 | **0.72** | 1.00 | -0.10 | 0.13 | --- | **0.63** | 0.48 | 0.26 | 0.27 |
|  | 0.61 | 0.74 | 0.62 | 0.23 | 0.43 | **4.2e-3** | 0 | 0.73 | 0.64 |  | **0.01** | 0.07 | 0.34 | 0.34 |
| **TLR8** | -0.05 | **0.66** | 0.16 | 0.37 | 0.31 | 0.07 | -0.10 | 1.00 | -0.27 | --- | 0.32 | 0.02 | 0.15 | -0.38 |
|  | 0.87 | **0.01** | 0.57 | 0.17 | 0.25 | 0.80 | 0.73 | 0 | 0.33 |  | 0.25 | 0.95 | 0.58 | 0.16 |
| **TLR9** | 0.16 | -0.29 | 0.09 | -0.38 | -0.10 | -0.17 | 0.13 | -0.27 | 1.00 | --- | 0.07 | 0.33 | 0.19 | 0.43 |
|  | 0.57 | 0.30 | 0.75 | 0.16 | 0.71 | 0.55 | 0.64 | 0.33 | 0 |  | 0.81 | 0.24 | 0.50 | 0.11 |
| **TLR10** | --- | --- | --- | --- | --- | --- | --- | --- | --- | --- | --- | --- | --- | --- |
| **RIG-I** | -0.18 | 0.33 | 0.22 | 0.41 | 0.07 | 0.46 | **0.63** | 0.32 | 0.07 | --- | 1.00 | 0.44 | **0.53** | -0.28 |
|  | 0.53 | 0.23 | 0.43 | 0.13 | 0.80 | 0.09 | **0.01** | 0.25 | 0.81 |  | 0 | 0.11 | **0.04** | 0.31 |
| **MDA5** | 0.06 | -0.02 | 0.26 | -0.29 | -0.10 | 0.50 | 0.48 | 0.02 | 0.33 | --- | 0.44 | 1.00 | **0.55** | 0.13 |
|  | 0.84 | 0.94 | 0.35 | 0.29 | 0.71 | 0.06 | 0.07 | 0.95 | 0.24 |  | 0.11 | 0 | **0.04** | 0.65 |
| **LGP2** | 0.09 | -0.07 | 0.22 | -0.15 | -0.21 | 0.30 | 0.26 | 0.15 | 0.19 | --- | **0.53** | **0.55** | 1.00 | -0.14 |
|  | 0.74 | 0.80 | 0.43 | 0.59 | 0.45 | 0.27 | 0.34 | 0.58 | 0.50 |  | **0.04** | **0.04** | 0 | 0.62 |
| **cGAS** | 0.25 | -0.51 | -0.39 | -0.42 | -0.08 | 0.04 | 0.27 | -0.38 | 0.43 | --- | -0.28 | 0.13 | -0.14 | 1.00 |
|  | 0.37 | 0.06 | 0.15 | 0.12 | 0.78 | 0.90 | 0.34 | 0.16 | 0.11 |  | 0.31 | 0.65 | 0.62 | 0 |

Correlations were assessed by Spearman R (top value) and statistical significance (p<0.05, bottom value) are indicated in blue. Transcripts expressed in less than 15% of patients were excluded from analysis. Abbreviations are as follows: TLR: Toll-like receptor, RIG-I: DDX58-RNA sensor RIG-I, MDA5: Melanoma differentiation-associated protein 5, LGP2: DHX58-DExH-box helicase 58, cGAS: Cyclic GMP-AMP synthase.
