# Supplementary material for: Pattern recognition receptor-associated immuno-thrombotic transcript changes in platelets and leukocytes with COVID19
Source: PLoS Pathog. 2025 Aug 18;21(8):e1013413. doi: 10.1371/journal.ppat.1013413 (PMC12373281; doi:10.1371/journal.ppat.1013413)
Supplement: S5 Table — (n = 15) Heatmap for Fig 1G. (DOCX) [file ppat.1013413.s007.docx]

**Table S4**: Correlation and significance in expression between pathogen-associated molecular pattern receptors among platelets of COVID19 patients. (n=10) *Heatmap for Fig. 1F*

| **TLR1** | **TLR2** | **TLR3** | **TLR4** | **TLR5** | **TLR6** | **TLR7** | **TLR8** | **TLR9** | **TLR10** | **RIG-I** | **MDA5** | **LGP2** | **cGAS** |
| --- | --- | --- | --- | --- | --- | --- | --- | --- | --- | --- | --- | --- | --- |
|  |  |  |  |  |  |  |  |  |  |  |  |  |  |

| Non-Infected  (% expressed) | 90 | 100 | 60 | 100 | 80 | 60 | 90 | 80 | 100 | 30 | 100 | 100 | 80 | 100 |
| --- | --- | --- | --- | --- | --- | --- | --- | --- | --- | --- | --- | --- | --- | --- |
| **TLR1** | 1.00 | 0.27 | 0.19 | 0.19 | 0.23 | -0.09 | 0.62 | **0.74** | -0.01 | --- | 0.62 | 0.38 | 0.56 | 0.42 |
|  | 0 | 0.45 | 0.59 | 0.61 | 0.52 | 0.80 | 0.06 | **0.02** | 1.00 |  | 0.06 | 0.28 | 0.10 | 0.23 |
| **TLR2** | 0.27 | 1.00 | 0.20 | **0.67** | 0.40 | **0.66** | 0.22 | **0.64** | 0.36 | --- | 0.21 | 0.14 | 0.21 | 0.19 |
|  | 0.45 | 0 | 0.58 | **0.04** | 0.25 | **0.05** | 0.54 | **0.05** | 031 |  | 0.56 | 0.71 | 0.56 | 0.61 |
| **TLR3** | 0.19 | 0.20 | 1.00 | -0.36 | -0.27 | 0.28 | 0.05 | 0.31 | 0.61 | --- | 0.15 | 0.23 | 0 | 0.61 |
|  | 0.59 | 0.58 | 0 | 0.30 | 0.45 | 0.42 | 0.89 | 0.38 | 0.07 |  | 0.68 | 0.53 | 1.00 | 0.07 |
| **TLR4** | 0.19 | **0.67** | -0.36 | 1.00 | 0.27 | 0.34 | 0.58 | 0.59 | -0.12 | --- | 0.36 | 0.32 | 0.49 | -0.01 |
|  | 0.61 | **0.04** | 0.30 | 0 | 0.45 | 0.33 | 0.09 | 0.08 | 0.76 |  | 0.31 | 0.37 | 0.16 | 1.00 |
| **TLR5** | 0.23 | 0.40 | -0.27 | 0.27 | 1.00 | 0.24 | -0.14 | 0.08 | 0.12 | --- | 0.07 | -0.15 | 0.01 | 0.17 |
|  | 0.52 | 0.25 | 0.45 | 0.45 | 0 | 0.50 | 0.70 | 0.83 | 0.74 |  | 0.86 | 0.69 | 0.98 | 0.64 |
| **TLR6** | -0.09 | **0.66** | 0.28 | 0.34 | 0.24 | 1.00 | -0.01 | 0.11 | **0.78** | --- | 0.39 | 0.45 | 0.36 | 0.39 |
|  | 0.80 | **0.05** | 0.42 | 0.33 | 0.50 | 0 | 0.98 | 0.76 | **0.01** |  | 0.26 | 0.19 | 0.30 | 0.26 |
| **TLR7** | 0.62 | 0.22 | 0.05 | 0.58 | -0.14 | -0.01 | 1.00 | **0.75** | -0.09 | --- | **0.75** | **0.73** | **0.74** | 0.48 |
|  | 0.06 | 0.54 | 0.89 | 0.09 | 0.70 | 0.98 | 0 | **0.02** | 0.81 |  | **0.02** | **0.02** | **0.02** | 0.17 |
| **TLR8** | **0.74** | **0.64** | 0.31 | 0.59 | 0.08 | 0.11 | **0.75** | 1.00 | 0 | --- | 0.46 | 0.35 | 0.48 | 0.36 |
|  | **0.02** | **0.05** | 0.38 | 0.08 | 0.83 | 0.76 | **0.02** | 0 | 1.00 |  | 0.18 | 0.32 | 0.17 | 0.30 |
| **TLR9** | -0.01 | 0.36 | 0.61 | -0.12 | 0.12 | **0.78** | -0.09 | 0 | 1.00 | --- | 0.39 | 0.44 | 0.29 | **0.65** |
|  | 1.00 | 0.31 | 0.07 | 0.76 | 0.74 | **0.01** | 0.81 | 1.00 | 0 |  | 0.26 | 0.20 | 0.42 | **0.05** |
| **TLR10** | --- | --- | --- | --- | --- | --- | --- | --- | --- | --- | --- | --- | --- | --- |
|  |  |  |  |  |  |  |  |  |  |  |  |  |  |  |
| **RIG-I** | 0.62 | 0.21 | 0.15 | 0.36 | 0.07 | 0.39 | **0.75** | 0.46 | 0.39 | --- | 1.00 | **0.94** | **0.94** | **0.70** |
|  | 0.06 | 0.56 | 0.68 | 0.31 | 0.86 | 0.26 | **0.02** | 0.18 | 0.26 |  | 0 | **2.06e-4** | **2.28e-4** | **0.03** |
| **MDA5** | 0.38 | 0.14 | 0.23 | 0.32 | -0.15 | 0.45 | **0.73** | 0.35 | 0.44 | --- | **0.94** | 1.00 | **0.86** | **0.72** |
|  | 0.28 | 0.71 | 0.53 | 0.37 | 0.69 | 0.19 | **0.02** | 0.32 | 0.20 |  | **2.06e-4** | 0 | **2.29e-3** | **0.02** |
| **LGP2** | 0.56 | 0.21 | 0 | 0.49 | 0.01 | 0.36 | **0.74** | 0.48 | 0.29 | --- | **0.94** | **0.86** | 1.00 | 0.50 |
|  | 0.10 | 0.56 | 1.00 | 0.16 | 0.98 | 0.30 | **0.02** | 0.17 | 0.42 |  | **2.28e-4** | **2.29e-3** | 0 | 0.15 |
| **cGAS** | 0.42 | 0.19 | 0.61 | -0.01 | 0.17 | 0.39 | 0.48 | 0.36 | **0.65** | --- | **0.70** | **0.72** | 0.50 | 1.00 |
|  | 0.23 | 0.61 | 0.07 | 1.00 | 0.64 | 0.26 | 0.17 | 0.30 | **0.05** |  | **0.03** | **0.02** | 0.15 | 0 |

Correlations were assessed by Spearman R (top value) and statistical significance (p<0.05, bottom value) are indicated in blue. Transcripts expressed in less than 40% of patients were excluded from analysis. Abbreviations are as follows: TLR: Toll-like receptor, RIG-I: DDX58-RNA sensor RIG-I, MDA5: Melanoma differentiation-associated protein 5, LGP2: DHX58-DExH-box helicase 58, cGAS: Cyclic GMP-AMP synthase.
