# Supplementary material for: Pattern recognition receptor-associated immuno-thrombotic transcript changes in platelets and leukocytes with COVID19
Source: PLoS Pathog. 2025 Aug 18;21(8):e1013413. doi: 10.1371/journal.ppat.1013413 (PMC12373281; doi:10.1371/journal.ppat.1013413)
Supplement: S6 Table — (n = 10) Heatmap for Fig 1H. (DOCX) [file ppat.1013413.s008.docx]

**Table S5**: Correlations in expression between pathogen-associated molecular pattern receptors among leukocytes of non-infected donors. (n=15) *Heatmap for Fig.1G*

| **TLR1** | **TLR2** | **TLR3** | **TLR4** | **TLR5** | **TLR6** | **TLR7** | **TLR8** | **TLR9** | **TLR10** | **RIG-I** | **MDA5** | **LGP2** | **cGAS** |
| --- | --- | --- | --- | --- | --- | --- | --- | --- | --- | --- | --- | --- | --- |
|  |  |  |  |  |  |  |  |  |  |  |  |  |  |

| Non-Infected (% expressed) | 100 | 100 | 73 | 100 | 100 | 100 | 100 | 100 | 100 | 80 | 100 | 100 | 100 | 100 |
| --- | --- | --- | --- | --- | --- | --- | --- | --- | --- | --- | --- | --- | --- | --- |
| **TLR1** | 1.00 | 0.40 | -0.04 | 0.33 | 0.30 | **0.64** | 0.08 | 0.43 | **0.53** | 0.38 | 0.40 | 0.32 | -0.06 | 0.43 |
|  | 0 | 0.14 | 0.90 | 0.23 | 0.28 | **0.01** | 0.78 | 0.12 | **0.05** | 0.16 | 0.14 | 0.24 | 0.84 | 0.11 |
| **TLR2** | 0.40 | 1.00 | -0.29 | **0.75** | -0.20 | **0.56** | **-0.61** | **0.75** | 0.32 | -0.51 | 0.26 | -0.03 | -0.32 | 0.07 |
|  | 0.14 | 0 | 0.29 | **1.87e-3** | 0.47 | **0.03** | **0.02** | **1.87e-3** | 0.25 | 0.06 | 0.34 | 0.92 | 0.24 | 0.81 |
| **TLR3** | -0.04 | -0.29 | 1.00 | -0.28 | 0.14 | -0.39 | 0.39 | -0.10 | 0.02 | 0.43 | -0.05 | -0.03 | 0.20 | 0.15 |
|  | 0.90 | 0.29 | 0 | 0.30 | 0.61 | 0.15 | 0.15 | 0.72 | 0.95 | 0.11 | 0.86 | 0.90 | 0.48 | 0.60 |
| **TLR4** | 0.33 | **0.75** | -0.28 | 1.00 | -0.26 | 0.36 | **-0.70** | **0.78** | 0.34 | -0.29 | 0.08 | -0.08 | -0.49 | 0.01 |
|  | 0.23 | **1.87e-3** | 0.30 | 0 | 0.35 | 0.19 | **4.51e-3** | **9.11e-4** | 0.22 | 0.30 | 0.78 | 0.77 | 0.07 | 0.97 |
| **TLR5** | 0.30 | -0.20 | 0.14 | -0.26 | 1.00 | 0.25 | 0.32 | 0 | 0.24 | 0.14 | -0.03 | 0.13 | 0.15 | **0.75** |
|  | 0.28 | 0.47 | 0.61 | 0.35 | 0 | 0.36 | 0.24 | 1.00 | 0.39 | 0.61 | 0.91 | 0.66 | 0.58 | **1.74e-3** |
| **TLR6** | **0.64** | **0.56** | -0.39 | 0.36 | 0.25 | 1.00 | -0.09 | **0.59** | **0.55** | -0.09 | **0.61** | 0.43 | 0.04 | **0.56** |
|  | **0.01** | **0.03** | 0.15 | 0.19 | 0.36 | 0 | 0.75 | **0.02** | **0.04** | 0.74 | **0.02** | 0.11 | 0.88 | **0.03** |
| **TLR7** | 0.08 | **-0.61** | 0.39 | **-0.70** | 0.32 | -0.09 | 1.00 | -0.46 | 0.23 | **0.61** | 0.39 | 0.50 | **0.72** | 0.25 |
|  | 0.78 | **0.02** | 0.15 | **4.51e-3** | 0.24 | 0.75 | 0 | 0.09 | 0.41 | **0.02** | 0.15 | 0.06 | **3.28e-3** | 0.36 |
| **TLR8** | 0.43 | **0.75** | -0.10 | **0.78** | 0 | **0.59** | -0.46 | 1.00 | **0.56** | -0.28 | 0.33 | 0.25 | -0.15 | 0.18 |
|  | 0.12 | **1.87e-3** | 0.72 | **9.11e-4** | 1.00 | **0.02** | 0.09 | 0 | **0.03** | 0.31 | 0.23 | 0.36 | 0.58 | 0.52 |
| **TLR9** | **0.53** | 0.32 | 0.02 | 0.34 | 0.24 | **0.55** | 0.23 | **0.56** | 1.00 | 0.03 | **0.62** | **0.74** | 0.33 | 0.39 |
|  | **0.05** | 0.25 | 0.95 | 0.22 | 0.39 | **0.04** | 0.41 | **0.03** | 0 | 0.93 | **0.02** | **2.33e-3** | 0.23 | 0.15 |
| **TLR10** | 0.38 | -0.51 | 0.43 | -0.29 | 0.14 | -0.09 | **0.61** | -0.28 | 0.03 | 1.00 | 0.17 | 0.22 | 0.26 | 0.21 |
|  | 0.16 | 0.06 | 0.11 | 0.30 | 0.61 | 0.74 | **0.02** | 0.31 | 0.93 | 0 | 0.55 | 0.42 | 0.35 | 0.45 |
| **RIG-I** | 0.40 | 0.26 | -0.05 | 0.08 | -0.03 | **0.61** | 0.39 | 0.33 | **0.62** | 0.17 | 1.00 | **0.83** | **0.66** | 0.32 |
|  | 0.14 | 0.34 | 0.86 | 0.78 | 0.91 | **0.02** | 0.15 | 0.23 | **0.02** | 0.55 | 0 | **2.57e-4** | **0.01** | 0.24 |
| **MDA5** | 0.32 | -0.03 | -0.03 | -0.08 | 0.13 | 0.43 | 0.50 | 0.25 | **0.74** | 0.22 | **0.83** | 1.00 | **0.76** | 0.24 |
|  | 0.24 | 0.92 | 0.90 | 0.77 | 0.66 | 0.11 | 0.06 | 0.36 | **2.33e-3** | 0.42 | **2.57e-4** | 0 | **1.49e-3** | 0.38 |
| **LGP2** | -0.06 | -0.32 | 0.20 | -0.49 | 0.15 | 0.04 | **0.72** | -0.15 | 0.33 | 0.26 | **0.66** | **0.76** | 1.00 | 0.19 |
|  | 0.84 | 0.24 | 0.48 | 0.07 | 0.58 | 0.88 | **3.28e-3** | 0.58 | 0.23 | 0.35 | **0.01** | **1.49e-3** | 0 | 0.51 |
| **cGAS** | 0.43 | 0.07 | 0.15 | 0.01 | **0.75** | **0.56** | 0.25 | 0.18 | 0.39 | 0.21 | 0.32 | 0.24 | 0.19 | 1.00 |
|  | 0.11 | 0.81 | 0.60 | 0.97 | **1.74e-3** | **0.03** | 0.36 | 0.52 | 0.15 | 0.45 | 0.24 | 0.38 | 0.51 | 0 |

Correlations were assessed by Spearman R (top value) and statistical significance (p<0.05, bottom value) are indicated in blue. Abbreviations are as follows: TLR: Toll-like receptor, RIG-I: DDX58-RNA sensor RIG-I, MDA5: Melanoma differentiation-associated protein 5, LGP2: DHX58-DExH-box helicase 58, cGAS: Cyclic GMP-AMP synthase.
