# Supplementary material for: Pattern recognition receptor-associated immuno-thrombotic transcript changes in platelets and leukocytes with COVID19
Source: PLoS Pathog. 2025 Aug 18;21(8):e1013413. doi: 10.1371/journal.ppat.1013413 (PMC12373281; doi:10.1371/journal.ppat.1013413)
Supplement: S7 Table — (n = 15) Heatmap for Fig 2A. (DOCX) [file ppat.1013413.s009.docx]

**Table S6**: Correlation and significance in expression between pathogen-associated molecular pattern receptors among leukocytes of COVID19 patients. (n=10) *Heatmap for Fig. 1H*

| **TLR1** | **TLR2** | **TLR3** | **TLR4** | **TLR5** | **TLR6** | **TLR7** | **TLR8** | **TLR9** | **TLR10** | **RIG-I** | **MDA5** | **LGP2** | **cGAS** |
| --- | --- | --- | --- | --- | --- | --- | --- | --- | --- | --- | --- | --- | --- |
|  |  |  |  |  |  |  |  |  |  |  |  |  |  |

| Non-Infected  (% expressed) | 90 | 100 | 60 | 100 | 80 | 60 | 90 | 80 | 100 | 30 | 100 | 100 | 80 | 100 |
| --- | --- | --- | --- | --- | --- | --- | --- | --- | --- | --- | --- | --- | --- | --- |
| **TLR1** | 1.00 | 0.15 | 0.22 | -0.12 | -0.21 | 0.28 | 0.26 | 0.27 | 0.26 | 0.02 | 0.07 | 0.18 | 0.14 | 0.60 |
|  | 0 | 0.68 | 0.54 | 0.76 | 0.56 | 0.43 | 0.47 | 0.45 | 0.47 | 0.97 | 0.87 | 0.63 | 0.71 | 0.07 |
| **TLR2** | 0.15 | 1.00 | 0.27 | **0.75** | 0.53 | 0.55 | -0.22 | **0.73** | 0.47 | -0.01 | 0.22 | 0.12 | 0.18 | 0.41 |
|  | 0.68 | 0 | 0.45 | **0.02** | 0.12 | 0.10 | 0.54 | **0.02** | 0.18 | 0.98 | 0.54 | 0.76 | 0.63 | 0.25 |
| **TLR3** | 0.22 | 0.27 | 1.00 | 0.44 | 0.33 | 0.61 | 0.25 | **0.66** | 0.36 | -0.04 | 0.59 | 0.50 | 0.56 | 0.49 |
|  | 0.54 | 0.45 | 0 | 0.20 | 0.35 | 0.07 | 0.49 | **0.04** | 0.31 | 0.93 | 0.08 | 0.14 | 0.10 | 0.15 |
| **TLR4** | -0.12 | **0.75** | 0.44 | 1.00 | **0.65** | 0.36 | -0.25 | **0.70** | 0.59 | -0.38 | 0.52 | 0.33 | 0.39 | 0.48 |
|  | 0.76 | **0.02** | 0.20 | 0 | **0.05** | 0.31 | 0.49 | **0.03** | 0.08 | 0.28 | 0.13 | 0.35 | 0.26 | 0.17 |
| **TLR5** | -0.21 | 0.53 | 0.33 | **0.65** | 1.00 | 0.01 | **-0.70** | 0.38 | 0.38 | -0.35 | -0.04 | -0.16 | -0.21 | 0.13 |
|  | 0.56 | 0.12 | 0.35 | **0.05** | 0 | 1.00 | **0.03** | 0.28 | 0.28 | 0.32 | 0.92 | 0.66 | 0.56 | 0.73 |
| **TLR6** | 0.28 | 0.55 | 0.61 | 0.36 | 0.01 | 1.00 | 0.21 | **0.81** | 0.18 | 0.21 | 0.39 | 0.30 | 0.44 | 0.54 |
|  | 0.43 | 0.10 | 0.07 | 0.31 | 1.00 | 0 | 0.56 | **0.01** | 0.63 | 0.55 | 0.26 | 0.41 | 0.20 | 0.11 |
| **TLR7** | 0.26 | -0.22 | 0.25 | -0.25 | **-0.70** | 0.21 | 1.00 | -0.09 | -0.08 | 0.26 | 0.58 | 0.59 | **0.70** | 0.09 |
|  | 0.47 | 0.54 | 0.49 | 0.49 | **0.03** | 0.56 | 0 | 0.81 | 0.84 | 0.46 | 0.09 | 0.08 | **0.03** | 0.81 |
| **TLR8** | 0.27 | **0.73** | **0.66** | **0.70** | 0.38 | **0.81** | -0.09 | 1.00 | 0.28 | 0.09 | 0.33 | 0.15 | 0.28 | 0.53 |
|  | 0.45 | **0.02** | **0.04** | **0.03** | 0.28 | **0.01** | 0.81 | 0 | 0.43 | 0.82 | 0.35 | 0.68 | 0.43 | 0.12 |
| **TLR9** | 0.26 | 0.47 | 0.36 | 0.59 | 0.38 | 0.18 | -0.08 | 0.28 | 1.00 | -0.41 | 0.56 | **0.68** | 0.54 | **0.77** |
|  | 0.47 | 0.18 | 0.31 | 0.08 | 0.28 | 0.63 | 0.84 | 0.43 | 0 | 0.23 | 0.10 | **0.03** | 0.11 | **0.01** |
| **TLR10** | 0.02 | -0.01 | -0.04 | -0.38 | -0.35 | 0.21 | 0.26 | 0.09 | -0.41 | 1.00 | -0.09 | -0.30 | -0.13 | -0.29 |
|  | 0.97 | 0.98 | 0.93 | 0.28 | 0.32 | 0.55 | 0.46 | 0.82 | 0.23 | 0 | 0.80 | 0.40 | 0.72 | 0.42 |
| **RIG-I** | 0.07 | 0.22 | 0.59 | 0.52 | -0.04 | 0.39 | 0.58 | 0.33 | 0.56 | -0.09 | 1.00 | **0.84** | **0.95** | 0.60 |
|  | 0.87 | 0.54 | 0.08 | 0.13 | 0.92 | 0.26 | 0.09 | 0.35 | 0.10 | 0.80 | 0 | **3.71e-3** | **1.08e-4** | 0.07 |
| **MDA5** | 0.18 | 0.12 | 0.50 | 0.33 | -0.16 | 0.30 | 0.59 | 0.15 | **0.68** | -0.30 | **0.84** | 1.00 | **0.94** | 0.59 |
|  | 0.63 | 0.76 | 0.14 | 0.35 | 0.66 | 0.41 | 0.08 | 0.68 | **0.03** | 0.40 | **3.71e-3** | 0 | **2.06e-4** | 0.08 |
| **LGP2** | 0.14 | 0.18 | 0.56 | 0.39 | -0.21 | 0.44 | **0.70** | 0.28 | 0.54 | -0.13 | **0.95** | **0.94** | 1.00 | 0.58 |
|  | 0.71 | 0.63 | 0.10 | 0.26 | 0.56 | 0.20 | **0.03** | 0.43 | 0.11 | 0.72 | **1.08e-4** | **2.06e-4** | 0 | 0.09 |
| **cGAS** | 0.60 | 0.41 | 0.49 | 0.48 | 0.13 | 0.54 | 0.09 | 0.53 | **0.77** | -0.29 | 0.60 | 0.59 | 0.58 | 1.00 |
|  | 0.07 | 0.25 | 0.15 | 0.17 | 0.73 | 0.11 | 0.81 | 0.12 | **0.01** | 0.42 | 0.07 | 0.08 | 0.09 | 0 |

Correlations were assessed by Spearman R (top value) and statistical significance (p<0.05, bottom value) are indicated in blue. Abbreviations are as follows: TLR: Toll-like receptor, RIG-I: DDX58-RNA sensor RIG-I, MDA5: Melanoma differentiation-associated protein 5, LGP2: DHX58-DExH-box helicase 58, cGAS: Cyclic GMP-AMP synthase.
