# Supplementary material for: Pattern recognition receptor-associated immuno-thrombotic transcript changes in platelets and leukocytes with COVID19
Source: PLoS Pathog. 2025 Aug 18;21(8):e1013413. doi: 10.1371/journal.ppat.1013413 (PMC12373281; doi:10.1371/journal.ppat.1013413)
Supplement: S8 Table — (n = 10) Heatmap for Fig 2B. (DOCX) [file ppat.1013413.s010.docx]

**Table S7**: Correlation and significance in expression between pathogen-associated molecular pattern receptors among platelets of non-infected donors. (n=15) *Heatmap for Fig. 2A*

|  | **F2R** | **F2RL3** | **P2RY1** | **P2RY12** | **P2RX1** | **TBXAR2** | **ITGA2B** | **vWF** | **GP6** | **GPB1A** | **GP5** | **GP9** | **SERPINE1** | **SERPINE2** | **SERPING1** | **PLAUR** | **TFPI** | **F13A1** | **SELP** | **SELPLG** | **CD40** | **CD40LG** |
| --- | --- | --- | --- | --- | --- | --- | --- | --- | --- | --- | --- | --- | --- | --- | --- | --- | --- | --- | --- | --- | --- | --- |
| Non-Infected  (% expressed) | 100 | 100 | 100 | 100 | 100 | 100 | 100 | 100 | 100 | 100 | 100 | 100 | 100 | 100 | 100 | 93 | 100 | 100 | 100 | 100 | 100 | 100 |
| **TLR1** | 0.19 | -0.11 | 0.35 | -0.30 | 0.30 | 0.24 | 0.48 | 0.05 | 0.18 | -0.01 | 0.25 | -0.08 | -0.03 | -0.07 | 0.43 | 0.11 | 0.11 | 0.18 | -0.13 | 0.39 | -0.01 | -0.30 |
|  | 0.50 | 0.70 | 0.19 | 0.27 | 0.27 | 0.39 | 0.07 | 0.87 | 0.53 | 0.96 | 0.36 | 0.77 | 0.92 | 0.80 | 0.11 | 0.70 | 0.69 | 0.51 | 0.66 | 0.15 | 0.96 | 0.27 |
| **TLR2** | 0.05 | -0.16 | -0.24 | -0.01 | -0.15 | -0.33 | -0.04 | -0.20 | -0.17 | -0.39 | -0.29 | -0.11 | -0.19 | -0.01 | 0.15 | **0.81** | 0.38 | 0.08 | -0.13 | **0.51** | -0.16 | -0.14 |
|  | 0.86 | 0.57 | 0.38 | 0.98 | 0.59 | 0.23 | 0.88 | 0.47 | 0.54 | 0.15 | 0.29 | 0.69 | 0.49 | 0.96 | 0.58 | **4.57e-4** | 0.16 | 0.79 | 0.65 | **0.05** | 0.57 | 0.61 |
| **TLR3** | -0.43 | -0.16 | -0.44 | -0.21 | 0.08 | -0.21 | 0.01 | -0.03 | -0.02 | -0.33 | 0.02 | -0.32 | -0.15 | -0.39 | 0.21 | 0.15 | 2.55e-3 | **0.58** | 0.20 | -0.05 | 0.05 | -0.05 |
|  | 0.11 | 0.59 | 0.10 | 0.46 | 0.79 | 0.45 | 0.97 | 0.92 | 0.95 | 0.24 | 0.96 | 0.25 | 0.61 | 0.15 | 0.46 | 0.60 | 1.00 | **0.02** | 0.49 | 0.86 | 0.88 | 0.85 |
| **TLR4** | 0.24 | -0.10 | -0.19 | 0.34 | -0.37 | -0.18 | -0.26 | -0.20 | -0.08 | 0.13 | -0.36 | 0.18 | -0.09 | 0.43 | -0.12 | **0.58** | **0.57** | -0.26 | -0.06 | 0.49 | -0.09 | -0.01 |
|  | 0.39 | 0.71 | 0.50 | 0.22 | 0.18 | 0.52 | 0.34 | 0.47 | 0.79 | 0.64 | 0.18 | 0.53 | 0.74 | 0.11 | 0.67 | **0.03** | **0.03** | 0.34 | 0.82 | 0.07 | 0.75 | 0.96 |
| **TLR5** | 0.46 | **-0.60** | 0.38 | -0.17 | 0.12 | -0.07 | -0.10 | -0.37 | -0.31 | 0.02 | -0.08 | -0.26 | 0.13 | 0.30 | 0.41 | 0.27 | **0.25** | -0.20 | 0.06 | 0.17 | -0.24 | 0.03 |
|  | 0.09 | **0.02** | 0.16 | 0.56 | 0.66 | 0.80 | 0.73 | 0.17 | 0.25 | 0.94 | 0.77 | 0.35 | 0.65 | 0.28 | 0.13 | 0.33 | **0.37** | 0.47 | 0.85 | 0.54 | 0.38 | 0.91 |
| **TLR6** | 0.13 | 0.28 | -0.13 | **0.54** | -0.12 | -0.34 | -0.29 | -0.32 | -0.09 | -0.07 | -0.35 | 0.02 | 0.11 | 0.25 | -0.26 | 0.30 | **0.69** | 0.45 | 0.02 | 0.51 | 0.11 | 0.15 |
|  | 0.65 | 0.31 | 0.64 | **0.04** | 0.67 | 0.22 | 0.29 | 0.24 | 0.76 | 0.81 | 0.20 | 0.95 | 0.70 | 0.36 | 0.36 | 0.28 | **0.01** | 0.09 | 0.95 | 0.06 | 0.71 | 0.60 |
| **TLR7** | **0.52** | 0.22 | 0.15 | **0.55** | 0.03 | 3.64e-3 | -0.24 | -0.24 | 0.01 | 0.33 | -0.19 | 0.26 | 0.27 | **0.79** | -0.02 | 0.29 | **0.78** | 0.22 | 0.27 | 0.48 | 0.43 | 0.17 |
|  | **0.05** | 0.43 | 0.60 | **0.04** | 0.92 | 0.99 | 0.39 | 0.39 | 0.98 | 0.23 | 0.49 | 0.34 | 0.33 | **8.53e-4** | 0.94 | 0.29 | **1.01e-3** | 0.42 | 0.33 | 0.07 | 0.11 | 0.55 |
| **TLR8** | -0.14 | -0.25 | -0.30 | 0.17 | -0.20 | -0.35 | -0.05 | -0.10 | -0.33 | -0.40 | -0.20 | -0.17 | -0.18 | -0.15 | 0.23 | **0.61** | 0.30 | 0.03 | -0.01 | 0.34 | -0.38 | 0.17 |
|  | 0.62 | 0.37 | 0.28 | 0.55 | 0.47 | 0.20 | 0.86 | 0.72 | 0.23 | 0.14 | 0.47 | 0.55 | 0.52 | 0.60 | 0.42 | **0.02** | 0.28 | 0.93 | 0.96 | 0.22 | 0.16 | 0.54 |
| **TLR9** | 0.47 | 0.08 | 0.47 | 0.02 | **0.71** | **0.72** | **0.61** | **0.55** | **0.65** | 0.48 | **0.70** | 0.49 | **0.55** | 0.29 | 0.28 | -0.10 | -0.04 | -0.14 | **0.55** | -0.24 | 0.50 | 0.18 |
|  | 0.08 | 0.77 | 0.08 | 0.95 | **4.24e-3** | **3.50e-3** | **0.02** | **0.04** | **0.01** | 0.07 | **4.79e-3** | 0.07 | **0.04** | 0.30 | 0.31 | 0.72 | 0.88 | 0.62 | **0.03** | 0.38 | 0.06 | 0.52 |
| **TLR10** | -0.02 | 0.21 | -0.15 | 0.42 | -0.36 | 0.04 | -0.06 | -0.21 | 0.13 | 0.13 | 0.08 | 0.13 | 0.07 | 0.28 | 0.32 | -0.02 | 0.50 | **0.54** | -0.15 | **0.55** | 0.34 | 0.10 |
|  | 0.94 | 0.47 | 0.61 | 0.12 | 0.21 | 0.89 | 0.84 | 0.49 | 0.66 | 0.66 | 0.78 | 0.66 | 0.80 | 0.33 | 0.27 | 0.94 | 0.07 | **0.04** | 0.61 | **0.03** | 0.24 | 0.73 |
| **RIG-I** | 0.04 | 0.19 | -0.36 | 0.44 | -0.09 | -0.14 | -0.22 | 0.07 | -0.03 | 0 | -0.30 | 0.26 | -0.03 | **0.54** | -0.11 | **0.66** | **0.65** | 0.31 | 0.45 | 0.43 | **0.52** | 0.19 |
|  | 0.88 | 0.50 | 0.19 | 0.11 | 0.76 | 0.62 | 0.43 | 0.81 | 0.92 | 1.00 | 0.28 | 0.34 | 0.92 | **0.04** | 0.70 | **0.01** | **0.01** | 0.26 | 0.10 | 0.11 | **0.05** | 0.51 |
| **MDA5** | 0.12 | 0.36 | 0.01 | **0.54** | 0.06 | -0.04 | -0.13 | -0.02 | 0.16 | -0.10 | 0.15 | 0.28 | **0.53** | 0.15 | 0.04 | 0.05 | 0.32 | **0.53** | 0.23 | 0.09 | 0.48 | **0.53** |
|  | 0.67 | 0.19 | 0.97 | **0.04** | 0.84 | 0.89 | 0.66 | 0.95 | 0.57 | 0.71 | 0.59 | 0.32 | **0.05** | 0.58 | 0.88 | 0.85 | 0.24 | **0.05** | 0.42 | 0.76 | 0.07 | **0.04** |
| **LGP2** | -0.27 | **0.53** | -0.29 | 0.43 | -0.25 | 0.31 | 0.27 | 0.24 | 0.41 | 0.14 | 0.35 | **0.56** | 0.16 | 0.15 | 0.31 | 0.27 | 0.23 | **0.60** | -0.07 | 0.44 | **0.57** | 0.31 |
|  | 0.32 | **0.05** | 0.29 | 0.11 | 0.37 | 0.26 | 0.32 | 0.38 | 0.13 | 0.62 | 0.20 | **0.03** | 0.56 | 0.58 | 0.26 | 0.32 | 0.40 | **0.02** | 0.81 | 0.11 | **0.03** | 0.26 |
| **cGAS** | 0.50 | 0.08 | **0.76** | -0.08 | **0.58** | 0.36 | 0.25 | 0.29 | 0.09 | 0.30 | 0.25 | 0.13 | 0.20 | 0.18 | 0 | -0.48 | -0.06 | -0.28 | 0.27 | -0.20 | 0.04 | -0.06 |
|  | 0.06 | 0.77 | **1.49e-3** | 0.78 | **0.03** | 0.19 | 0.36 | 0.30 | 0.75 | 0.28 | 0.36 | 0.66 | 0.47 | 0.52 | 1.00 | 0.07 | 0.82 | 0.31 | 0.33 | 0.48 | 0.88 | 0.82 |

Correlations were assessed by Spearman R (top value) and statistical significance (p<0.05, bottom value) are indicated in blue. Abbreviations are as follows: Abbreviations are as follows: TLR: Toll-like receptor, RIG-I: DDX58-RNA sensor RIG-I, MDA5: Melanoma differentiation-associated protein 5, LGP2: DHX58-DExH-box helicase 58, cGAS: Cyclic GMP-AMP synthase, F2R: coagulation factor II (thrombin), F2RL3: coagulation factor II (thrombin) receptor-like 3, P2RY1: Purinergic Receptor P2Y1, P2RY12: Purinergic Receptor P2Y12, P2RX1: Purinergic Receptor P2X1, TBXA2R: Thromboxane A2 receptor, ITGA2B: Integrin alphaIIb/beta3 (αIIbβ3) receptor complex, vWF: Von Willebrand factor, GP6: Glycoprotein VI, GP1BA: Glycoprotein 1b subunit alpha, GP5: Glycoprotein V, GP9: Glycoprotein IX, SERPINE1: Serpin family E member 1, SERPINE2: Serpin family E member 2, SERPING1: Serpin family G member 1, PLAUR: Plasminogen activator urokinase receptor, TFPI: Tissue Factor Pathway inhibitor, F13A1: Coagulation Factor XIII A chain, SELP: P-selectin, SELPLG: P-selectin ligand, CD40, CD40LG: CD40 ligand
