# Supplementary material for: Pattern recognition receptor-associated immuno-thrombotic transcript changes in platelets and leukocytes with COVID19
Source: PLoS Pathog. 2025 Aug 18;21(8):e1013413. doi: 10.1371/journal.ppat.1013413 (PMC12373281; doi:10.1371/journal.ppat.1013413)
Supplement: S9 Table — (n = 15) Heatmap for Fig 2D. (DOCX) [file ppat.1013413.s011.docx]

**Table S8**: Correlation and significance in expression between pathogen-associated molecular pattern receptors and prothrombotic or coagulation-associated gene transcripts among platelets of COVID19 patients. (n=10) *Heatmap for Fig. 2B*

|  | **F2R** | **F2RL3** | **P2RY1** | **P2RY12** | **P2RX1** | **TBXAR2** | **ITGA2B** | **vWF** | **GP6** | **GPB1A** | **GP5** | **GP9** | **SERPINE1** | **SERPINE2** | **SERPING1** | **PLAUR** | **TFPI** | **F13A1** | **SELP** | **SELPLG** | **CD40** | **CD40LG** |
| --- | --- | --- | --- | --- | --- | --- | --- | --- | --- | --- | --- | --- | --- | --- | --- | --- | --- | --- | --- | --- | --- | --- |
| Non-Infected  (% expressed) | 100 | 100 | 100 | 100 | 100 | 100 | 100 | 100 | 100 | 100 | 100 | 100 | 100 | 100 | 100 | 93 | 100 | 100 | 100 | 100 | 100 | 100 |
| **TLR1** | 0.32 | 0.19 | **0.72** | 0.07 | -0.01 | 0.27 | 0.13 | -0.07 | 0.16 | 0.28 | 0.01 | 0.05 | 0.22 | -0.07 | 0.32 | 0.59 | -0.31 | -0.53 | 0.22 | 0.52 | 0.36 | 0.31 |
|  | 0.37 | 0.61 | **0.02** | 0.87 | 1.00 | 0.45 | 0.73 | 0.87 | 0.66 | 0.43 | 1.00 | 0.89 | 0.54 | 0.87 | 0.37 | 0.08 | 0.39 | 0.12 | 0.54 | 0.13 | 0.31 | 0.39 |
| **TLR2** | 0.20 | 0.21 | -0.05 | -0.05 | 0.44 | 0.05 | 0.61 | 0.35 | 0.41 | -0.14 | -0.35 | 0.03 | -0.01 | 0.44 | 0.16 | 0.61 | 0.08 | 0.30 | 0.37 | **0.81** | **0.71** | 0.13 |
|  | 0.58 | 0.56 | 0.89 | 0.89 | 0.20 | 0.89 | 0.07 | 0.33 | 0.25 | 0.71 | 0.33 | 0.95 | 1.00 | 0.20 | 0.66 | 0.07 | 0.84 | 0.41 | 0.30 | **0.01** | **0.03** | 0.7 |
| **TLR3** | 0.08 | 0.28 | 0.16 | -0.02 | 0.27 | 0.44 | 0.18 | -0.39 | 0.46 | 0.48 | 0.14 | -0.05 | 0.45 | 0.02 | 0.63 | -0.12 | 0.01 | -0.10 | 0.16 | 0.24 | 0.37 | 0.63 |
|  | 0.82 | 0.43 | 0.65 | 0.97 | 0.45 | 0.20 | 0.63 | 0.27 | 0.18 | 0.16 | 0.69 | 0.89 | 0.19 | 0.97 | 0.06 | 0.74 | 0.98 | 0.78 | 0.65 | 0.50 | 0.29 | 0.06 |
| **TLR4** | 0.53 | 0.18 | -0.16 | 0.32 | -0.15 | 0.19 | 0.18 | 0.27 | 0.28 | -0.22 | -0.19 | 0.43 | 0.03 | 0.58 | 0.08 | **0.81** | 0.44 | 0.48 | 0.25 | 0.47 | 0.54 | -0.27 |
|  | 0.12 | 0.63 | 0.66 | 0.37 | 0.68 | 0.61 | 0.63 | 0.45 | 0.43 | 0.54 | 0.61 | 0.22 | 0.95 | 0.09 | 0.84 | **0.01** | 0.20 | 0.17 | 0.49 | 0.18 | 0.11 | 0.45 |
| **TLR5** | -0.04 | 0.05 | 0.34 | -0.38 | 0.47 | -0.27 | 0.60 | **0.70** | 0.14 | -0.55 | 0.08 | -0.13 | **-0.65** | -0.29 | -0.32 | 0.36 | -0.20 | -0.12 | 0.52 | 0.36 | 0.11 | -0.44 |
|  | 0.93 | 0.90 | 0.33 | 0.28 | 0.17 | 0.45 | 0.07 | **0.03** | 0.70 | 0.10 | 0.83 | 0.71 | **0.05** | 0.41 | 0.36 | 0.31 | 0.58 | 0.74 | 0.13 | 0.31 | 0.77 | 0.20 |
| **TLR6** | 0.42 | **0.69** | -0.28 | 0.27 | **0.72** | 0.29 | 0.46 | 0.15 | **0.76** | 0.18 | -0.14 | 0.39 | 0.23 | 0.29 | 0.33 | 0.31 | 0.28 | 0.52 | 0.53 | 0.59 | **0.81** | 0.29 |
|  | 0.23 | **0.03** | 0.44 | 0.45 | **0.02** | 0.41 | 0.18 | 0.68 | **0.02** | 0.63 | 0.70 | 0.27 | 0.52 | 0.41 | 0.36 | 0.37 | 0.43 | 0.13 | 0.12 | 0.08 | **0.01** | 0.42 |
| **TLR7** | **0.77** | 0.38 | 0.39 | 0.58 | -0.43 | **0.64** | -0.15 | -0.20 | 0.31 | 0.39 | 0.27 | 0.59 | 0.55 | 0.30 | 0.43 | **0.76** | 0.28 | -0.02 | 0.10 | 0.18 | 0.50 | 0.25 |
|  | **0.01** | 0.28 | 0.26 | 0.09 | 0.22 | **0.05** | 0.68 | 0.58 | 0.39 | 0.26 | 0.45 | 0.08 | 0.10 | 0.41 | 0.22 | **0.01** | 0.43 | 0.97 | 0.79 | 0.63 | 0.14 | 0.49 |
| **TLR8** | 0.41 | 0.11 | 0.32 | 0.09 | -0.15 | 0.34 | 0.27 | 0.01 | 0.29 | 0.10 | -0.04 | 0.12 | 0.26 | 0.39 | 0.44 | **0.77** | 0.06 | -0.08 | 0.27 | 0.62 | 0.56 | 0.22 |
|  | 0.24 | 0.77 | 0.37 | 0.80 | 0.69 | 0.33 | 0.45 | 0.99 | 0.41 | 0.78 | 0.91 | 0.75 | 0.46 | 0.27 | 0.21 | **0.01** | 0.87 | 0.83 | 0.45 | 0.06 | 0.10 | 0.54 |
| **TLR9** | 0.31 | **0.78** | -0.01 | 0.18 | **0.71** | 0.48 | 0.36 | -0.10 | **0.49** | 0.44 | 0.15 | 0.33 | 0.36 | 0.19 | 0.33 | -0.07 | 0.09 | 0.36 | 0.49 | 0.36 | 0.56 | 0.45 |
|  | 0.39 | **0.01** | 1.00 | 0.63 | **0.03** | 0.17 | 0.31 | 0.79 | **0.01** | 0.20 | 0.68 | 0.35 | 0.31 | 0.61 | 0.35 | 0.87 | 0.81 | 0.31 | 0.15 | 0.31 | 0.10 | 0.19 |
| **TLR10** | 0.35 | -0.07 | -0.17 | 0.45 | -0.45 | 0.17 | -0.26 | -0.10 | -0.08 | 0.02 | -0.40 | 0.35 | 0.28 | 0.54 | 0.05 | 0.51 | 0.44 | -0.50 | -0.37 | 0.14 | 0.32 | -0.07 |
|  | 0.33 | 0.87 | 0.64 | 0.19 | 0.19 | 0.64 | 0.47 | 0.80 | 0.84 | 0.96 | 0.26 | 0.33 | 0.45 | 0.11 | 0.91 | 0.14 | 0.21 | 0.16 | 0.31 | 0.71 | 0.37 | 0.87 |
| **RIG-I** | **0.89** | **0.82** | 0.43 | **0.71** | 0.07 | **0.77** | -0.09 | -0.27 | **0.67** | 0.62 | 0.20 | **0.77** | **0.64** | 0.13 | 0.55 | 0.62 | 0.26 | 0.03 | 0.35 | 0.36 | **0.72** | 0.39 |
|  | **1.15e-3** | **0.01** | 0.22 | **0.03** | 0.87 | **0.01** | 0.81 | 0.45 | **0.04** | 0.06 | 0.58 | **0.01** | **0.05** | 0.73 | 0.10 | 0.06 | 0.47 | 0.95 | 0.33 | 0.31 | **0.02** | 0.26 |
| **MDA5** | **0.94** | **0.87** | 0.27 | **0.83** | 0.01 | **0.85** | -0.18 | -0.36 | **0.70** | **0.71** | 0.30 | **0.87** | **0.77** | 0.18 | 0.60 | 0.53 | 0.42 | 0.18 | 0.25 | 0.18 | **0.73** | 0.48 |
|  | **2.06e-4** | **2.17e-3** | 0.45 | **4.71e-3** | 1.00 | **2.86e-3** | 0.63 | 0.31 | **0.03** | **0.03** | 0.41 | **2.17e-3** | **0.01** | 0.63 | 0.07 | 0.12 | 0.23 | 0.63 | 0.49 | 0.63 | **0.02** | 0.17 |
| **LGP2** | **0.88** | **0.74** | 0.18 | **0.67** | -0.04 | **0.70** | -0.10 | -0.24 | **0.67** | 0.48 | 0.12 | **0.77** | 0.55 | 0.35 | 0.52 | **0.64** | 0.33 | 0.21 | 0.44 | 0.43 | **0.66** | 0.19 |
|  | **1.72e-3** | **0.02** | 0.61 | **0.04** | 0.93 | **0.03** | 0.78 | 0.50 | **0.04** | 0.16 | 0.75 | **0.01** | 0.10 | 0.32 | 0.13 | **0.05** | 0.34 | 0.55 | 0.20 | 0.22 | **0.04** | 0.59 |
| **cGAS** | **0.64** | **0.73** | 0.55 | 0.35 | 0.25 | **0.73** | 0.20 | -0.14 | **0.71** | 0.50 | 0.59 | 0.49 | 0.45 | -0.13 | 0.53 | 0.35 | 0.16 | -0.08 | 0.44 | 0.15 | 0.58 | 0.45 |
|  | **0.05** | **0.02** | 0.10 | 0.33 | 0.49 | **0.02** | 0.58 | 0.71 | **0.03** | 0.14 | 0.08 | 0.15 | 0.19 | 0.73 | 0.12 | 0.33 | 0.66 | 0.84 | 0.20 | 0.68 | 0.09 | 0.19 |

Correlations were assessed by Spearman R (top value) and statistical significance (p<0.05, bottom value) are indicated in blue. Abbreviations are as follows: Abbreviations are as follows: TLR: Toll-like receptor, RIG-I: DDX58-RNA sensor RIG-I, MDA5: Melanoma differentiation-associated protein 5, LGP2: DHX58-DExH-box helicase 58, cGAS: Cyclic GMP-AMP synthase, F2R: coagulation factor II (thrombin), F2RL3: coagulation factor II (thrombin) receptor-like 3, P2RY1: Purinergic Receptor P2Y1, P2RY12: Purinergic Receptor P2Y12, P2RX1: Purinergic Receptor P2X1, TBXA2R: Thromboxane A2 receptor, ITGA2B: Integrin alphaIIb/beta3 (αIIbβ3) receptor complex, vWF: Von Willebrand factor, GP6: Glycoprotein VI, GP1BA: Glycoprotein 1b subunit alpha, GP5: Glycoprotein V, GP9: Glycoprotein IX, SERPINE1: Serpin family E member 1, SERPINE2: Serpin family E member 2, SERPING1: Serpin family G member 1, PLAUR: Plasminogen activator urokinase receptor, TFPI: Tissue Factor Pathway inhibitor, F13A1: Coagulation Factor XIII A chain, SELP: P-selectin, SELPLG: P-selectin ligand, CD40, CD40LG: CD40 ligand
