# Supplementary material for: Pattern recognition receptor-associated immuno-thrombotic transcript changes in platelets and leukocytes with COVID19
Source: PLoS Pathog. 2025 Aug 18;21(8):e1013413. doi: 10.1371/journal.ppat.1013413 (PMC12373281; doi:10.1371/journal.ppat.1013413)
Supplement: S10 Table — (n = 10) Heatmap for Fig 2E. (DOCX) [file ppat.1013413.s012.docx]

**Table S9**: Correlations in expression between pathogen-associated molecular pattern receptors and thrombolytic or cell-cell interaction-associated gene transcripts among leukocytes of non-infected donors. (n=15) *Heatmap for Fig. 2D*

| **PLAUR** | **PLAU** | **TFPI** | **PROC** | **F3** | **SERPING1** | **F13A1** | **CD40** | **CD40LG** | **SELPLG** |
| --- | --- | --- | --- | --- | --- | --- | --- | --- | --- |

| **TLR1** | -0.02 | -0.12 | -0.19 | **0.57** | -0.41 | 0.18 | 0.50 | 0.02 | -0.04 | 0.28 |
| --- | --- | --- | --- | --- | --- | --- | --- | --- | --- | --- |
|  | 0.95 | 0.68 | 0.50 | **0.03** | 0.13 | 0.52 | 0.06 | 0.95 | 0.88 | 0.31 |
| **TLR2** | 0.34 | 0.16 | -0.32 | 0.21 | -0.30 | 0.19 | 0.24 | **-0.53** | -0.40 | **0.78** |
|  | 0.22 | 0.58 | 0.24 | 0.44 | 0.28 | 0.49 | 0.40 | **0.04** | 0.14 | **9.11e-4** |
| **TLR3** | -0.23 | -0.26 | **0.82** | -0.10 | -0.06 | -0.14 | 0.22 | 0.35 | 0.43 | -0.37 |
|  | 0.40 | 0.34 | **4.01e-4** | 0.71 | 0.83 | 0.61 | 0.43 | 0.20 | 0.11 | 0.18 |
| **TLR4** | 0.43 | **0.57** | -0.31 | 0.41 | -0.20 | 0.07 | -0.10 | -0.39 | -0.48 | **0.82** |
|  | 0.12 | **0.03** | 0.25 | 0.13 | 0.49 | 0.81 | 0.73 | 0.15 | 0.07 | **3.54e-4** |
| **TLR5** | -0.39 | **-0.55** | 0.20 | -0.01 | -0.14 | 0.11 | 0.10 | -0.04 | -0.01 | -0.24 |
|  | 0.15 | **0.04** | 0.47 | 0.97 | 0.62 | 0.70 | 0.73 | 0.90 | 0.98 | 0.40 |
| **TLR6** | 0.23 | 0 | -0.30 | 0.38 | -0.04 | 0.42 | 0.37 | -0.16 | -0.20 | **0.56** |
|  | 0.41 | 1.0 | 0.28 | 0.17 | 0.89 | 0.12 | 0.18 | 0.57 | 0.47 | **0.03** |
| **TLR7** | -0.09 | -0.37 | 0.29 | 0.08 | 0.22 | 0.23 | **0.54** | **0.70** | **0.79** | **-0.59** |
|  | 0.76 | 0.18 | 0.29 | 0.77 | 0.43 | 0.42 | **0.04** | **4.85e-3** | **7.65e-4** | **0.02** |
| **TLR8** | 0.42 | 0.37 | -0.04 | 0.40 | -0.40 | 0.20 | 0.23 | -0.47 | -0.17 | **0.78** |
|  | 0.12 | 0.17 | 0.88 | 0.14 | 0.14 | 0.47 | 0.41 | 0.08 | 0.55 | **9.11e-4** |
| **TLR9** | 0.50 | 0.14 | -0.03 | **0.78** | -0.18 | 0.35 | **0.61** | 0.02 | 0.19 | **0.55** |
|  | 0.06 | 0.62 | 0.92 | **1.08e-3** | 0.53 | 0.20 | **0.02** | 0.94 | 0.49 | **0.04** |
| **TLR10** | -0.27 | -0.02 | 0.14 | 0.13 | 0.08 | 0.04 | 0.20 | **0.69** | 0.46 | -0.44 |
|  | 0.32 | 0.94 | 0.61 | 0.65 | 0.79 | 0.88 | 0.48 | **0.01** | 0.09 | 0.10 |
| **RIG-I** | **0.65** | -0.06 | -0.04 | 0.42 | 0.06 | **0.80** | **0.68** | 0.43 | 0.43 | 0.33 |
|  | **0.01** | 0.83 | 0.88 | 0.12 | 0.84 | **5.28e-4** | **0.01** | 0.11 | 0.12 | 0.24 |
| **MDA5** | **0.60** | -3.57e-3 | -0.01 | 0.50 | -0.10 | **0.64** | **0.63** | 0.31 | **0.53** | 0.24 |
|  | **0.02** | 0.99 | 0.96 | 0.06 | 0.74 | **0.01** | **0.01** | 0.26 | **0.04** | 0.38 |
| **LGP2** | 0.35 | -0.39 | 0.16 | 0.03 | 0.16 | **0.56** | **0.52** | **0.55** | **0.81** | -0.28 |
|  | 0.20 | 0.16 | 0.57 | 0.93 | 0.58 | **0.03** | **0.05** | **0.04** | **4.34e-4** | 0.31 |
| **cGAS** | -0.17 | -0.43 | 0.13 | 0.02 | 0.26 | 0.28 | 0.18 | 0.14 | -0.03 | 0.08 |
|  | 0.54 | 0.11 | 0.64 | 0.94 | 0.36 | 0.31 | 0.52 | 0.62 | 0.93 | 0.79 |

Correlations were assessed by Spearman R (top value) and statistical significance (p<0.05, bottom value) are indicated in blue. Abbreviations are as follows: TLR: Toll-like receptor, RIG-I: DDX58-RNA sensor RIG-I, MDA5: Melanoma differentiation-associated protein 5, LGP2: DHX58-DExH-box helicase 58, cGAS: Cyclic GMP-AMP synthase, PLAUR: Plasminogen activator urokinase receptor, PLAU: Plasminogen activator urokinase, TFPI: Tissue factor pathway inhibitor, PROC: Protein C, F3: Coagulation Factor III (Thromboplastin), SERPING1: Serpin family G member 1, F13A1: Coagulation factor XIII A chain, CD40, CD40LG: CD40 ligand, SELPLG: P-selectin ligand.
