# Supplementary material for: Pattern recognition receptor-associated immuno-thrombotic transcript changes in platelets and leukocytes with COVID19
Source: PLoS Pathog. 2025 Aug 18;21(8):e1013413. doi: 10.1371/journal.ppat.1013413 (PMC12373281; doi:10.1371/journal.ppat.1013413)
Supplement: S11 Table — (n = 15) Heatmap for Fig 5A. (DOCX) [file ppat.1013413.s013.docx]

**Table S10**: Correlations in expression between pathogen-associated molecular pattern receptors and thrombolytic and cell-cell interaction-associated gene transcripts among leukocytes of COVID19 patients. (n=10) *Heatmap for Fig. 2E*

| **PLAUR** | **PLAU** | **TFPI** | **PROC** | **F3** | **SERPING1** | **F13A1** | **CD40** | **CD40LG** | **SELPLG** |
| --- | --- | --- | --- | --- | --- | --- | --- | --- | --- |

| **TLR1** | 0.09 | 0.08 | -0.25 | -0.12 | 0.25 | 0.25 | 0.47 | 0.30 | 0.25 | 0.27 |
| --- | --- | --- | --- | --- | --- | --- | --- | --- | --- | --- |
|  | 0.81 | 0.84 | 0.49 | 0.76 | 0.48 | 0.49 | 0.18 | 0.41 | 0.49 | 0.45 |
| **TLR2** | 0.15 | 0.53 | -0.32 | **-0.73** | 0.05 | 0.10 | 0.42 | -0.08 | -0.43 | 0.38 |
|  | 0.68 | 0.12 | 0.37 | **0.02** | 0.91 | 0.79 | 0.23 | 0.84 | 0.22 | 0.28 |
| **TLR3** | **0.67** | -0.13 | 0.14 | -0.43 | 0.28 | 0.61 | 0.29 | 0.24 | 0.04 | 0.19 |
|  | **0.04** | 0.73 | 0.71 | 0.22 | 0.44 | 0.07 | 0.15 | 0.51 | 0.92 | 0.61 |
| **TLR4** | 0.26 | 0.44 | -0.04 | -0.62 | 0.20 | 0.39 | 0.32 | -0.04 | -0.55 | 0.48 |
|  | 0.47 | 0.20 | 0.92 | 0.06 | 0.58 | 0.26 | 0.37 | 0.92 | 0.10 | 0.17 |
| **TLR5** | -0.03 | 0.44 | -0.02 | **-0.77** | 0.30 | -0.01 | 0.20 | -0.43 | **-0.81** | 0.43 |
|  | 0.95 | 0.20 | 0.97 | **0.01** | 0.39 | 1.00 | 0.58 | 0.22 | **0.01** | 0.22 |
| **TLR6** | **0.72** | 0.02 | -0.04 | -0.53 | 0.16 | 0.24 | **0.70** | 0.16 | 0.09 | 0.37 |
|  | **0.02** | 0.97 | 0.92 | 0.12 | 0.66 | 0.51 | **0.03** | 0.66 | 0.81 | 0.30 |
| **TLR7** | 0.18 | -0.49 | 0.15 | 0.60 | -0.08 | 0.49 | 0.01 | 0.61 | **0.89** | -0.53 |
|  | 0.63 | 0.15 | 0.68 | 0.07 | 0.83 | 0.15 | 1.00 | 0.07 | **1.15e-3** | 0.12 |
| **TLR8** | **0.75** | 0.48 | -0.19 | **-0.76** | 0.06 | 0.20 | 0.56 | -0.10 | -0.37 | 0.45 |
|  | **0.02** | 0.17 | 0.61 | **0.01** | 0.88 | 0.58 | 0.10 | 0.79 | 0.30 | 0.19 |
| **TLR9** | -0.10 | -0.16 | -0.28 | -0.27 | 0.32 | **0.72** | 0.39 | 0.55 | -0.12 | 0.56 |
|  | 0.79 | 0.66 | 0.43 | 0.45 | 0.37 | **0.02** | 0.26 | 0.10 | 0.76 | 0.10 |
| **TLR10** | 0.38 | 0.08 | -0.61 | 0.21 | -0.51 | -0.42 | 0.02 | -0.21 | 0.19 | -0.39 |
|  | 0.28 | 0.83 | 0.07 | 0.56 | 0.14 | 0.23 | 0.95 | 0.56 | 0.59 | 0.27 |
| **RIG-I** | 0.32 | -0.31 | 0.01 | 0.10 | 0.29 | **0.81** | 0.42 | 0.55 | 0.32 | 0.13 |
|  | 0.37 | 0.39 | 1.00 | 0.79 | 0.41 | **0.01** | 0.23 | 0.10 | 0.37 | 0.73 |
| **MDA5** | 0.12 | -0.58 | 0.12 | 0.19 | 0.16 | **0.96** | 0.20 | **0.89** | 0.52 | 0.07 |
|  | 0.76 | 0.09 | 0.76 | 0.61 | 0.66 | **4.91e-5** | 0.58 | **1.15e-3** | 0.13 | 0.87 |
| **LGP2** | 0.30 | -0.45 | 0.10 | 0.18 | 0.19 | **0.88** | 0.32 | **0.75** | 0.52 | 0.03 |
|  | 0.41 | 0.19 | 0.79 | 0.63 | 0.61 | **1.60e-3** | 0.37 | **0.02** | 0.13 | 0.95 |
| **cGAS** | 0.26 | -0.08 | -0.20 | -0.35 | 0.56 | 0.62 | **0.79** | 0.44 | 0.01 | **0.76** |
|  | 0.47 | 0.84 | 0.58 | 0.33 | 0.10 | 0.06 | **0.01** | 0.20 | 1.00 | **0.01** |

Correlations were assessed by Spearman R (top value) and statistical significance (p<0.05, bottom value) are indicated in blue. Abbreviations are as follows: TLR: Toll-like receptor, RIG-I: DDX58-RNA sensor RIG-I, MDA5: Melanoma differentiation-associated protein 5, LGP2: DHX58-DExH-box helicase 58, cGAS: Cyclic GMP-AMP synthase, PLAUR: Plasminogen activator urokinase receptor, PLAU: Plasminogen activator urokinase, TFPI: Tissue factor pathway inhibitor, PROC: Protein C, F3: Coagulation Factor III (Thromboplastin), SERPING1: Serpin family G member 1, F13A1: Coagulation factor XIII A chain, CD40, CD40LG: CD40 ligand, SELPLG: P-selectin ligand.
