# Supplementary material for: Pattern recognition receptor-associated immuno-thrombotic transcript changes in platelets and leukocytes with COVID19
Source: PLoS Pathog. 2025 Aug 18;21(8):e1013413. doi: 10.1371/journal.ppat.1013413 (PMC12373281; doi:10.1371/journal.ppat.1013413)
Supplement: S12 Table — (n = 10) Heatmap for Fig 5B. (DOCX) [file ppat.1013413.s014.docx]

**Table S11:** Correlation and significance in expression between pathogen-associated molecular pattern receptors in platelets (purple) and leukocytes (light orange) in platelets from non-infected donors. (n=15) *Heatmap for Fig. 5A*

|  | **TLR1_L** | **TLR2_L** | **TLR3_L** | **TLR4_L** | **TLR5_L** | **TLR6_L** | **TLR7_L** | **TLR8_L** | **TLR9_L** | **TLR10_L** | **RIG-I** | **MDA5_L** | **LGP2_L** | **cGAS_L** |
| --- | --- | --- | --- | --- | --- | --- | --- | --- | --- | --- | --- | --- | --- | --- |
| **TLR1** | 0.47 | 0.20 | -0.15 | 0.16 | 0.37 | 0.36 | 0.09 | 0.07 | 0.19 | 0.01 | -0.02 | -0.16 | -0.24 | 0.51 |
|  | 0.08 | 0.47 | 0.60 | 0.57 | 0.18 | 0.19 | 0.74 | 0.82 | 0.50 | 0.96 | 0.95 | 0.57 | 0.38 | 0.05 |
| **TLR2** | 0.29 | **0.53** | 0.11 | **0.58** | 0.10 | 0.29 | -0.34 | **0.72** | 0.35 | -0.31 | 0.02 | -0.11 | -0.32 | 0.06 |
|  | 0.29 | **0.04** | 0.69 | **0.03** | 0.72 | 0.30 | 0.22 | **3.18e-3** | 0.20 | 0.26 | 0.95 | 0.69 | 0.24 | 0.82 |
| **TLR3** | 0.15 | 0.27 | -0.10 | -0.11 | 0.19 | 0.27 | 0.16 | 0.15 | **0.58** | -0.46 | 0.26 | 0.41 | 0.20 | 0.06 |
|  | 0.60 | 0.33 | 0.72 | 0.70 | 0.50 | 0.33 | 0.58 | 0.60 | **0.02** | 0.09 | 0.36 | 0.13 | 0.48 | 0.82 |
| **TLR4** | 0.07 | 0.31 | 0.14 | 0.38 | -0.18 | 0.09 | -0.31 | 0.37 | -0.03 | -0.02 | -0.03 | -0.26 | -0.35 | -0.14 |
|  | 0.80 | 0.26 | 0.63 | 0.16 | 0.52 | 0.74 | 0.26 | 0.18 | 0.91 | 0.94 | 0.93 | 0.34 | 0.20 | 0.63 |
| **TLR5** | 0.04 | 0.08 | -0.08 | 0.23 | 0.31 | 0.39 | -0.13 | 0.37 | 0.10 | -0.10 | -0.16 | -0.24 | -0.34 | 0.35 |
|  | 0.89 | 0.79 | 0.79 | 0.40 | 0.26 | 0.15 | 0.65 | 0.18 | 0.72 | 0.73 | 0.57 | 0.39 | 0.22 | 0.20 |
| **TLR6** | 0.48 | 0.06 | 0.05 | 0.11 | -0.32 | 0.15 | 0.37 | 0.04 | 0.39 | 0.38 | 0.40 | 0.28 | 0.12 | -0.15 |
|  | 0.07 | 0.82 | 0.85 | 0.69 | 0.25 | 0.59 | 0.18 | 0.89 | 0.15 | 0.16 | 0.15 | 0.31 | 0.68 | 0.59 |
| **TLR7** | 0.12 | -0.12 | 0.08 | 0.02 | -0.34 | 0.18 | 0.42 | 0.01 | 0.24 | 0.42 | 0.45 | 0.29 | 0.14 | -0.01 |
|  | 0.67 | 0.68 | 0.77 | 0.95 | 0.22 | 0.51 | 0.13 | 0.99 | 0.38 | 0.12 | 0.10 | 0.30 | 0.61 | 0.98 |
| **TLR8** | -0.06 | 0.16 | -0.15 | 0.07 | 0.11 | 0.09 | -0.11 | 0.35 | 0.05 | -0.42 | 0.03 | 0.03 | 0.08 | -0.24 |
|  | 0.83 | 0.58 | 0.60 | 0.80 | 0.70 | 0.74 | 0.70 | 0.20 | 0.85 | 0.12 | 0.92 | 0.92 | 0.78 | 0.40 |
| **TLR9** | -0.14 | -0.40 | 0.10 | -0.30 | 0.29 | 0.01 | 0.29 | -0.23 | 0.13 | 0.08 | 0.15 | 0.34 | 0.30 | 0.44 |
|  | 0.61 | 0.14 | 0.73 | 0.27 | 0.29 | 0.99 | 0.29 | 0.40 | 0.66 | 0.79 | 0.59 | 0.22 | 0.27 | 0.10 |
| **RIG-I** | -0.24 | -0.15 | 0.46 | -0.20 | -0.20 | -0.17 | 0.45 | -0.01 | 0.25 | 0.04 | 0.31 | 0.25 | 0.33 | -0.19 |
|  | 0.40 | 0.59 | 0.09 | 0.47 | 0.47 | 0.55 | 0.10 | 0.99 | 0.36 | 0.88 | 0.26 | 0.37 | 0.24 | 0.51 |
| **MDA5** | 0.08 | -0.13 | 0.10 | -0.04 | -0.13 | 0.11 | **0.55** | 0.15 | **0.63** | 0.21 | **0.61** | **0.76** | **0.70** | 0.08 |
|  | 0.77 | 0.64 | 0.71 | 0.88 | 0.66 | 0.70 | **0.04** | 0.60 | **0.01** | 0.45 | **0.02** | **1.49e-3** | **4.79e-3** | 0.79 |
| **LGP2** | 0.04 | -0.26 | 0.43 | **-0.54** | 0.27 | -0.08 | **0.83** | -0.22 | 0.22 | 0.32 | 0.47 | 0.46 | **0.79** | 0.23 |
|  | 0.90 | 0.34 | 0.11 | **0.04** | 0.33 | 0.77 | **2.87e-4** | 0.43 | 0.42 | 0.25 | 0.08 | 0.09 | **7.91e-4** | 0.40 |
| **cGAS** | -0.15 | -0.36 | -0.42 | -0.05 | -0.13 | -0.05 | 0.10 | -0.41 | -0.24 | 0.21 | 0.06 | 0.02 | 0.04 | 0.08 |
|  | 0.59 | 0.19 | 0.12 | 0.87 | 0.64 | 0.86 | 0.71 | 0.13 | 0.38 | 0.46 | 0.83 | 0.94 | 0.90 | 0.79 |

Correlations were assessed by Spearman R (top value) and statistical significance (p<0.05, bottom value) are indicated in blue. Abbreviations are as follows: TLR: Toll-like receptor, RIG-I: DDX58-RNA sensor RIG-I, MDA5: Melanoma differentiation-associated protein 5, LGP2: DHX58-DExH-box helicase 58, cGAS: Cyclic GMP-AMP synthase.
