# Supplementary material for: Pattern recognition receptor-associated immuno-thrombotic transcript changes in platelets and leukocytes with COVID19
Source: PLoS Pathog. 2025 Aug 18;21(8):e1013413. doi: 10.1371/journal.ppat.1013413 (PMC12373281; doi:10.1371/journal.ppat.1013413)
Supplement: S13 Table — (n = 15) Heatmap for Fig 5C. (DOCX) [file ppat.1013413.s015.docx]

**Table S12:** Correlation and significance in expression between pathogen-associated molecular pattern receptors in platelets (purple) and leukocytes (light orange) in platelets from COVID19 patients. (n=10) *Heatmap for Fig. 5B*

|  | **TLR1_L** | **TLR2_L** | **TLR3_L** | **TLR4_L** | **TLR5_L** | **TLR6_L** | **TLR7_L** | **TLR8_L** | **TLR9_L** | **TLR10_L** | **RIG-I** | **MDA5_L** | **LGP2_L** | **cGAS_L** |
| --- | --- | --- | --- | --- | --- | --- | --- | --- | --- | --- | --- | --- | --- | --- |
| **TLR1** | 0.19 | 0.54 | 0.22 | 0.31 | 0.29 | 0.22 | -0.09 | 0.24 | **0.78** | 0.03 | 0.25 | 0.47 | 0.30 | 0.43 |
|  | 0.61 | 0.11 | 0.54 | 0.39 | 0.43 | 0.54 | 0.81 | 0.51 | **0.01** | 0.94 | 0.49 | 0.18 | 0.41 | 0.22 |
| **TLR2** | -0.12 | **0.66** | -0.19 | 0.60 | 0.14 | 0.10 | -0.10 | 0.38 | 0.21 | -0.09 | 0.09 | 0.09 | 0.12 | 0.01 |
|  | 0.76 | **0.04** | 0.61 | 0.07 | 0.71 | 0.79 | 0.79 | 0.28 | 0.56 | 0.80 | 0.81 | 0.81 | 0.76 | 1.00 |
| **TLR3** | -0.45 | 0.05 | 0.28 | 0.49 | 0.51 | -0.10 | -0.38 | 0.13 | 0.37 | **-0.65** | 0.10 | 0.29 | 0.13 | 0.03 |
|  | 0.19 | 0.89 | 0.43 | 0.15 | 0.14 | 0.78 | 0.27 | 0.73 | 0.29 | **0.05** | 0.78 | 0.41 | 0.73 | 0.95 |
| **TLR4** | -0.14 | 0.47 | -0.16 | 0.29 | -0.19 | 0.15 | 0.33 | 0.21 | 0.01 | 0.51 | 0.33 | 0.10 | 0.29 | -0.09 |
|  | 0.71 | 0.18 | 0.66 | 0.43 | 0.61 | 0.68 | 0.35 | 0.56 | 1.00 | 0.14 | 0.35 | 0.79 | 0.43 | 0.81 |
| **TLR5** | **0.77** | 0.42 | 0.24 | 0.21 | -0.12 | 0.38 | 0.16 | 0.60 | 0.20 | 0.20 | 0.09 | 0.10 | 0.13 | 0.45 |
|  | **0.01** | 0.23 | 0.51 | 0.55 | 0.74 | 0.27 | 0.66 | 0.07 | 0.58 | 0.58 | 0.82 | 0.78 | 0.71 | 0.19 |
| **TLR6** | -0.04 | 0.33 | 0.08 | 0.54 | -0.18 | 0.34 | 0.29 | 0.36 | 0.18 | -0.41 | 0.46 | 0.47 | 0.54 | 0.28 |
|  | 0.92 | 0.35 | 0.84 | 0.11 | 0.63 | 0.34 | 0.42 | 0.30 | 0.61 | 0.23 | 0.18 | 0.17 | 0.11 | 0.43 |
| **TLR7** | -0.26 | 0.20 | 0.10 | 0.24 | 0.02 | -0.07 | 0.31 | -0.09 | 0.52 | 0.25 | 0.59 | 0.55 | 0.54 | 0.10 |
|  | 0.47 | 0.58 | 0.79 | 0.51 | 0.97 | 0.87 | 0.39 | 0.81 | 0.13 | 0.48 | 0.08 | 0.10 | 0.11 | 0.79 |
| **TLR8** | -0.32 | 0.61 | 0.04 | 0.57 | 0.41 | 0.02 | -0.21 | 0.26 | 0.54 | 0.13 | 0.26 | 0.24 | 0.20 | 0.06 |
|  | 0.37 | 0.07 | 0.93 | 0.09 | 0.23 | 0.95 | 0.55 | 0.47 | 0.11 | 0.73 | 0.47 | 0.50 | 0.59 | 0.87 |
| **TLR9** | 0.03 | 0.24 | 0.44 | 0.58 | 0.15 | 0.25 | 0.21 | 0.35 | 0.35 | **-0.72** | 0.47 | 0.60 | 0.56 | 0.33 |
|  | 0.95 | 0.51 | 0.20 | 0.09 | 0.68 | 0.49 | 0.56 | 0.33 | 0.33 | **0.02** | 0.18 | 0.07 | 0.10 | 0.35 |
| **RIG-I** | 0.07 | 0.26 | 0.38 | 0.30 | -0.19 | 0.35 | 0.59 | 0.14 | **0.65** | -0.05 | **0.79** | **0.93** | **0.89** | 0.47 |
|  | 0.87 | 0.47 | 0.28 | 0.41 | 0.61 | 0.33 | 0.08 | 0.71 | **0.05** | 0.90 | **0.01** | **3.35e-4** | **1.15e-3** | 0.18 |
| **MDA5** | -0.14 | 0.03 | 0.30 | 0.25 | -0.26 | 0.16 | **0.64** | -0.04 | 0.52 | -0.13 | **0.83** | **0.92** | **0.90** | 0.32 |
|  | 0.71 | 0.95 | 0.41 | 0.49 | 0.47 | 0.66 | **0.05** | 0.92 | 0.13 | 0.71 | **4.71e-3** | **5.33e-4** | **8.07e-4** | 0.37 |
| **LGP2** | -0.01 | 0.41 | 0.43 | 0.38 | -0.10 | 0.51 | 0.55 | 0.27 | 0.56 | 0.10 | **0.83** | **0.80** | **0.86** | 0.46 |
|  | 0.99 | 0.23 | 0.22 | 0.28 | 0.79 | 0.14 | 0.10 | 0.44 | 0.10 | 0.77 | **4.90e-3** | **0.01** | **2.29e-3** | 0.18 |
| **cGAS** | 0.04 | 0.06 | 0.55 | 0.47 | 0.09 | 0.07 | 0.31 | 0.21 | **0.66** | -0.38 | **0.69** | **0.84** | **0.73** | 0.43 |
|  | 0.92 | 0.89 | 0.10 | 0.18 | 0.81 | 0.87 | 0.39 | 0.56 | **0.04** | 0.27 | **0.03** | **3.71e-3** | **0.02** | 0.22 |

Correlations were assessed by Spearman R (top value) and statistical significance (p<0.05, bottom value) are indicated in blue. Abbreviations are as follows: TLR: Toll-like receptor, RIG-I: DDX58-RNA sensor RIG-I, MDA5: Melanoma differentiation-associated protein 5, LGP2: DHX58-DExH-box helicase 58, cGAS: Cyclic GMP-AMP synthase.
