# Supplementary material for: Pattern recognition receptor-associated immuno-thrombotic transcript changes in platelets and leukocytes with COVID19
Source: PLoS Pathog. 2025 Aug 18;21(8):e1013413. doi: 10.1371/journal.ppat.1013413 (PMC12373281; doi:10.1371/journal.ppat.1013413)
Supplement: S14 Table — (n = 10) Heatmap for Fig 5D. (DOCX) [file ppat.1013413.s016.docx]

**Table S13:** Correlation and significance in expression between pathogen-associated molecular pattern receptors in platelets (purple) and coagulation or platelet-leukocyte interaction related transcripts in leukocytes (light orange) in platelets from noninfected donors. (n=15) *Heatmap for Fig. 5C*

|  | **PLAUR_L** | **PLAU_L** | **TFPI_L** | **PROC_L** | **F3_L** | **SERPING1_L** | **F13A1_L** | **CD40_L** | **CD40LG_L** | **SELPLG_L** |
| --- | --- | --- | --- | --- | --- | --- | --- | --- | --- | --- |
| **TLR1_P** | -0.25 | -0.25 | -0.08 | 0.11 | 0.20 | -0.20 | 0.18 | -0.08 | -0.11 | -0.01 |
|  | 0.37 | 0.37 | 0.77 | 0.69 | 0.47 | 0.47 | 0.51 | 0.78 | 0.70 | 0.96 |
| **TLR2_P** | 0.13 | 0.16 | 0.28 | 0.43 | -0.39 | -0.11 | 0.08 | -0.31 | -0.14 | 0.41 |
|  | 0.66 | 0.57 | 0.31 | 0.11 | 0.16 | 0.69 | 0.79 | 0.26 | 0.63 | 0.14 |
| **TLR3_P** | 0.26 | -0.20 | 3.95e-3 | 0.34 | -0.29 | 0.13 | **0.58** | -0.35 | 0.03 | 0.28 |
|  | 0.35 | 0.48 | 0.99 | 0.21 | 0.52 | 0.64 | **0.02** | 0.20 | 0.92 | 0.31 |
| **TLR4_P** | -0.06 | 0.29 | 0.13 | 0.27 | -0.14 | -0.08 | -0.26 | 0.06 | -0.18 | 0.27 |
|  | 0.84 | 0.29 | 0.63 | 0.33 | 0.62 | 0.78 | 0.34 | 0.82 | 0.52 | 0.33 |
| **TLR5_P** | -0.27 | 0.24 | 0.10 | 0.05 | 0.22 | -0.27 | -0.20 | -0.21 | -0.22 | 0.11 |
|  | 0.34 | 0.39 | 0.73 | 0.86 | 0.44 | 0.32 | 0.47 | 0.44 | 0.43 | 0.69 |
| **TLR6_P** | 0.19 | 0.16 | -0.05 | **0.67** | 0.02 | -0.03 | 0.45 | 0.47 | 0.39 | 0.02 |
|  | 0.49 | 0.57 | 0.85 | **0.01** | 0.95 | 0.93 | 0.09 | 0.08 | 0.15 | 0.95 |
| **TLR7_P** | 0.22 | 0.40 | 0.09 | 0.39 | 0.42 | 0.10 | 0.22 | **0.58** | 0.38 | 0.07 |
|  | 0.43 | 0.14 | 0.75 | 0.15 | 0.12 | 0.72 | 0.42 | **0.03** | 0.16 | 0.81 |
| **TLR8_P** | 0.15 | -0.08 | 0.18 | 0.18 | -0.42 | 0.04 | 0.03 | -0.22 | 0.13 | -0.01 |
|  | 0.59 | 0.77 | 0.51 | 0.53 | 0.13 | 0.88 | 0.93 | 0.43 | 0.65 | 0.96 |
| **TLR9_P** | 0.05 | -0.26 | 0.34 | -0.10 | 0.41 | 0.09 | -0.14 | 0.22 | 0.17 | -0.08 |
|  | 0.86 | 0.35 | 0.22 | 0.73 | 0.13 | 0.75 | 0.62 | 0.43 | 0.54 | 0.79 |
| **RIG-I_P** | 0.28 | 0.10 | **0.56** | 0.29 | 0.11 | 0.10 | 0.31 | 0.43 | **0.52** | -0.07 |
|  | 0.32 | 0.71 | **0.03** | 0.29 | 0.71 | 0.72 | 0.26 | 0.11 | **0.05** | 0.80 |
| **MDA5_P** | **0.53** | 011 | 0.07 | 0.40 | 0.24 | 0.24 | **0.53** | 0.39 | **0.74** | 0.08 |
|  | **0.05** | 0.70 | 0.80 | 0.14 | 0.40 | 0.38 | **0.05** | 0.15 | **2.17e-3** | 0.77 |
| **LGP2_P** | 0.04 | **-0.54** | 0.34 | -0.04 | 0.12 | 0.32 | **0.60** | **0.55** | **0.86** | -0.46 |
|  | 0.89 | **0.04** | 0.21 | 0.89 | 0.68 | 0.24 | **0.02** | **0.04** | **8.64e-5** | 0.09 |
| **cGAS_P** | 0.17 | 0.16 | -0.30 | -0.13 | **0.57** | 0.19 | -0.28 | 0.36 | -0.06 | -0.15 |
|  | 0.54 | 0.58 | 0.28 | 0.64 | **0.03** | 0.51 | 0.31 | 0.18 | 0.83 | 0.59 |

Correlations were assessed by Spearman R (top value) and statistical significance (p<0.05, bottom value) are indicated in blue. Abbreviations are as follows: TLR: Toll-like receptor, RIG-I: DDX58-RNA sensor RIG-I, MDA5: Melanoma differentiation-associated protein 5, LGP2: DHX58-DExH-box helicase 58, cGAS: Cyclic GMP-AMP synthase, PLAUR: Plasminogen activator urokinase receptor, PLAU: Plasminogen activator urokinase, TFPI: Tissue factor pathway inhibitor, PROC: Protein C, F3: Coagulation Factor III (Thromboplastin), SERPING1: Serpin family G member 1, F13A1: Coagulation factor XIII A chain, CD40, CD40LG: CD40 Ligand, SELPLG: P-selectin ligand.
