# Supplementary material for: Pattern recognition receptor-associated immuno-thrombotic transcript changes in platelets and leukocytes with COVID19
Source: PLoS Pathog. 2025 Aug 18;21(8):e1013413. doi: 10.1371/journal.ppat.1013413 (PMC12373281; doi:10.1371/journal.ppat.1013413)
Supplement: S15 Table — (n = 15) Heatmap for Fig 5E. (DOCX) [file ppat.1013413.s017.docx]

**Table S14:** Correlation and significance in expression between pathogen-associated molecular pattern receptors in platelets (purple) and coagulation or platelet-leukocyte interaction related transcripts in leukocytes (light orange) in platelets from COVID19 patients. (n=10) *Heatmap for Fig. 5D*

|  | **PLAUR_L** | **PLAU_L** | **TFPI_L** | **PROC_L** | **F3_L** | **SERPING1_L** | **F13A1_L** | **CD40_L** | **CD40LG_L** | **SELPLG_L** |
| --- | --- | --- | --- | --- | --- | --- | --- | --- | --- | --- |
| **TLR1_P** | -0.10 | -0.18 | -0.54 | -0.26 | -0.14 | 0.43 | 0.18 | 0.50 | -0.03 | 0.25 |
|  | 0.79 | 0.63 | 0.11 | 0.47 | 0.71 | 0.22 | 0.63 | 0.14 | 0.95 | 0.49 |
| **TLR2_P** | -0.03 | 0.55 | -0.22 | -0.24 | -0.46 | 0.03 | -0.25 | 0.01 | -0.31 | -0.02 |
|  | 0.95 | 0.10 | 0.54 | 0.51 | 0.19 | 0.95 | 0.49 | 1.00 | 0.39 | 0.97 |
| **TLR3_P** | 0.04 | -0.10 | 0.39 | -0.30 | -0.03 | 0.39 | -0.28 | 0.15 | -0.36 | 0.19 |
|  | 0.91 | 0.78 | 0.26 | 0.40 | 0.95 | 0.26 | 0.43 | 0.68 | 0.30 | 0.59 |
| **TLR4_P** | 0.08 | 0.32 | -0.49 | 0.16 | -0.47 | -0.05 | -0.09 | 0.01 | 0.07 | -0.32 |
|  | 0.84 | 0.37 | 0.15 | 0.66 | 0.17 | 0.89 | 0.81 | 1.00 | 0.87 | 0.37 |
| **TLR5_P** | 0.39 | 0.46 | -0.46 | -0.26 | -0.23 | 0.15 | 0.24 | 0.13 | -0.03 | 0.17 |
|  | 0.27 | 0.18 | 0.19 | 0.47 | 0.52 | 0.67 | 0.51 | 0.71 | 0.94 | 0.64 |
| **TLR6_P** | 0.18 | 0.12 | 0.34 | -0.06 | -0.02 | 0.41 | 0.01 | 0.32 | 0.10 | 0.10 |
|  | 0.61 | 0.74 | 0.33 | 0.88 | 0.96 | 0.24 | 0.99 | 0.37 | 0.78 | 0.78 |
| **TLR7_P** | -0.13 | -032 | -0.45 | 0.31 | -0.24 | 0.44 | -0.08 | 0.45 | 0.21 | -0.21 |
|  | 0.73 | 0.37 | 0.19 | 0.39 | 0.51 | 0.20 | 0.84 | 0.19 | 0.56 | 0.56 |
| **TLR8_P** | -0.06 | 0.19 | -0.55 | -0.21 | -0.42 | 0.18 | -0.14 | 0.12 | -0.34 | 0.03 |
|  | 0.87 | 0.59 | 0.10 | 0.55 | 0.23 | 0.61 | 0.70 | 0.74 | 0.33 | 0.94 |
| **TLR9_P** | 0.14 | -0.05 | 0.62 | -0.22 | 0.21 | 0.67 | 0.01 | 0.41 | 0.07 | 0.12 |
|  | 0.71 | 0.89 | 0.06 | 0.54 | 0.55 | 0.04 | 1.00 | 0.25 | 0.87 | 0.76 |
| **RIG-I_P** | 0.07 | -0.55 | -0.09 | 0.20 | -0.02 | 0.82 | 0.16 | 0.85 | 0.53 | -0.04 |
|  | 0.87 | 0.10 | 0.81 | 0.58 | 0.97 | 0.01 | 0.66 | 2.86e-3 | 0.12 | 0.92 |
| **MDA5_P** | 0.02 | -0.62 | 0.09 | 0.39 | 0.01 | 0.81 | 0.02 | 0.81 | 0.56 | -0.15 |
|  | 0.97 | 0.06 | 0.81 | 0.26 | 1.00 | 0.01 | 0.97 | 0.01 | 0.10 | 0.68 |
| **LGP2_P** | 0.17 | -0.43 | -0.13 | 0.06 | 0.08 | 0.67 | 0.35 | 0.65 | 0.43 | 0.01 |
|  | 0.64 | 0.22 | 0.71 | 0.87 | 0.83 | 0.04 | 0.32 | 0.05 | 0.21 | 0.98 |
| **cGAS_P** | 0.20 | -0.33 | 0.08 | 0.08 | -0.05 | 0.90 | -0.07 | 0.68 | 0.18 | 0.04 |
|  | 0.58 | 0.35 | 0.84 | 0.84 | 0.91 | 8.07e-4 | 0.87 | 0.03 | 0.63 | 0.92 |

Correlations were assessed by Spearman R (top value) and statistical significance (p<0.05, bottom value) are indicated in blue. Abbreviations are as follows: TLR: Toll-like receptor, RIG-I: DDX58-RNA sensor RIG-I, MDA5: Melanoma differentiation-associated protein 5, LGP2: DHX58-DExH-box helicase 58, cGAS: Cyclic GMP-AMP synthase, PLAUR: Plasminogen activator urokinase receptor, PLAU: Plasminogen activator urokinase, TFPI: Tissue factor pathway inhibitor, PROC: Protein C, F3: Coagulation Factor III (Thromboplastin), SERPING1: Serpin family G member 1, F13A1: Coagulation factor XIII A chain, CD40, CD40LG: CD40 Ligand, SELPLG: P-selectin ligand.
