# Supplementary material for: Pattern recognition receptor-associated immuno-thrombotic transcript changes in platelets and leukocytes with COVID19
Source: PLoS Pathog. 2025 Aug 18;21(8):e1013413. doi: 10.1371/journal.ppat.1013413 (PMC12373281; doi:10.1371/journal.ppat.1013413)
Supplement: S16 Table — (n = 10) Heatmap for Fig 5F. (DOCX) [file ppat.1013413.s018.docx]

**Table S15:** Correlation and significance in expression between pathogen-associated molecular pattern receptors in leukocytes (light orange) and thrombosis-coagulation related transcripts in platelets (purple) from noninfected donors. (n=15) *Heatmap for Fig. 5E*

|  | **ITGA2B** | **vWF** | **GP6** | **GPB1A** | **GP5** | **GP9** | **SERPINE1** | **SERPINE2** | **SERPING1** | **PLAUR** | **F13A1** | **SELP** | **CD40** | **CD40LG** |
| --- | --- | --- | --- | --- | --- | --- | --- | --- | --- | --- | --- | --- | --- | --- |
| **TLR1_L** | 0.09 | -0.49 | 0.18 | 0.04 | 0.09 | -0.03 | 0.16 | -0.23 | 0.21 | 0.16 | -0.44 | **-0.56** | -0.18 | -0.12 |
|  | 0.76 | 0.07 | 0.53 | 0.90 | 0.74 | 0.91 | 0.56 | 0.40 | 0.46 | 0.56 | 0.10 | **0.03** | 0.52 | 0.67 |
| **TLR2_L** | -0.08 | -0.17 | -0.07 | -0.24 | -0.16 | -0.13 | -0.28 | -0.27 | 0.24 | 0.30 | -0.48 | -0.45 | -0.11 | -0.24 |
|  | 0.77 | 0.55 | 0.80 | 0.38 | 0.56 | 0.64 | 0.31 | 0.33 | 0.39 | 0.27 | 0.07 | 0.09 | 0.70 | 0.38 |
| **TLR3_L** | 0.04 | 0.14 | 0.24 | 0.07 | -0.06 | 0.37 | 0.01 | 0.24 | -0.21 | 0.40 | 0.09 | 0.01 | **0.56** | 0.02 |
|  | 0.89 | 0.63 | 0.39 | 0.81 | 0.84 | 0.18 | 0.99 | 0.38 | 0.44 | 0.14 | 0.75 | 0.98 | **0.03** | 0.94 |
| **TLR4_L** | -0.18 | -0.20 | -0.14 | -.034 | -0.26 | -0.14 | -0.11 | -0.18 | -0.08 | 0.20 | -0.21 | -0.27 | -0.21 | -0.27 |
|  | 0.52 | 0.48 | 0.61 | 0.21 | 0.35 | 0.61 | 0.70 | 0.53 | 0.77 | 0.47 | 0.44 | 0.33 | 0.46 | 0.33 |
| **TLR5_L** | **0.57** | 0.08 | 0.40 | 0.17 | **0.62** | 0.13 | 0.21 | -0.27 | **0.56** | 0.04 | -0.31 | -0.14 | -0.12 | -0.08 |
|  | **0.03** | 0.78 | 0.14 | 0.55 | **0.02** | 0.65 | 0.46 | 0.33 | **0.03** | 0.90 | 0.25 | 0.61 | 0.68 | 0.77 |
| **TLR6_L** | -0.01 | -0.44 | 0.01 | 0.26 | 0.20 | -0.07 | 0.33 | -0.03 | **0.63** | 0.12 | -0.25 | -0.27 | -0.17 | 0.14 |
|  | 0.97 | 0.11 | 0.97 | 0.34 | 0.48 | 0.80 | 0.23 | 0.91 | **0.01** | 0.67 | 0.36 | 0.33 | 0.54 | 0.61 |
| **TLR7_L** | 0.12 | 0.01 | 0.27 | 0.26 | 0.27 | 0.34 | 0.31 | 0.26 | 0.15 | 0.01 | 0.05 | 0.12 | 0.45 | 0.29 |
|  | 0.67 | 0.98 | 0.33 | 0.35 | 0.33 | 0.21 | 0.26 | 0.35 | 0.59 | 0.98 | 0.87 | 0.68 | 0.10 | 0.29 |
| **TLR8_L** | -0.16 | -0.39 | -0.10 | -0.32 | -0.11 | -0.08 | 0.05 | -0.13 | 0.29 | 0.47 | -0.27 | -0.37 | -0.09 | -0.03 |
|  | 0.58 | 0.15 | 0.71 | 0.25 | 0.70 | 0.77 | 0.86 | 0.64 | 0.29 | 0.08 | 0.33 | 0.17 | 0.76 | 0.92 |
| **TLR9_L** | -0.14 | -0.28 | 0.14 | -0.18 | 0.09 | 0.01 | 0.37 | -0.18 | 0.16 | 0.21 | -0.18 | 0.03 | 0.14 | 0.19 |
|  | 0.63 | 0.32 | 0.61 | 0.52 | 0.74 | 0.96 | 0.17 | 0.53 | 0.56 | 0.44 | 0.52 | 0.92 | 0.61 | 0.51 |
| **TLR10_L** | -0.02 | -0.29 | 0.25 | 0.37 | 0.11 | 0.34 | 0.36 | 0.34 | -0.17 | -0.13 | 0.05 | -0.25 | 0.24 | 0.10 |
|  | 0.95 | 0.29 | 0.37 | 0.18 | 0.69 | 0.21 | 0.19 | 0.21 | 0.55 | 0.66 | 0.86 | 0.37 | 0.39 | 0.71 |
| **RIG-I_L** | -0.08 | -0.12 | 0.20 | 0.28 | 0.18 | 0.38 | 0.40 | 0.11 | 0.34 | 0.20 | -0.22 | -0.10 | 0.35 | 0.38 |
|  | 0.77 | 0.67 | 0.48 | 0.31 | 0.52 | 0.16 | 0.14 | 0.69 | 0.22 | 0.47 | 0.43 | 0.71 | 0.21 | 0.16 |
| **MDA5_L** | -0.07 | -0.17 | 0.24 | 0.10 | 0.29 | 0.26 | **0.55** | -0.03 | 0.23 | 0.03 | -0.10 | 0.05 | 0.28 | 0.46 |
|  | 0.80 | 0.54 | 0.40 | 0.73 | 0.29 | 0.35 | **0.04** | 0.92 | 0.41 | 0.93 | 0.71 | 0.85 | 0.31 | 0.09 |
| **LGP2_L** | 0.05 | 0.18 | 0.30 | 0.20 | 0.39 | 0.49 | 0.48 | 0.01 | 0.22 | -0.05 | -0.05 | 0.06 | 0.42 | **0.63** |
|  | 0.86 | 0.52 | 0.28 | 0.48 | 0.15 | 0.07 | 0.07 | 0.96 | 0.43 | 0.85 | 0.85 | 0.83 | 0.12 | **0.01** |
| **cGAS_L** | **0.53** | 0.13 | **0.56** | 0.49 | **0.69** | 0.39 | 0.47 | 0.01 | **0.65** | 0 | -0.24 | -0.14 | 0.21 | 0.05 |
|  | **0.05** | 0.66 | **0.03** | 0.06 | **0.01** | 0.16 | 0.08 | 0.96 | **0.01** | 1.00 | 0.39 | 0.63 | 0.44 | 0.87 |

Correlations were assessed by Spearman R (top value) and statistical significance (p<0.05, bottom value) are indicated in blue. Abbreviations are as follows: TLR: Toll-like receptor, RIG-I: DDX58-RNA sensor RIG-I, MDA5: Melanoma differentiation-associated protein 5, LGP2: DHX58-DExH-box helicase 58, cGAS: Cyclic GMP-AMP synthase, ITGA2B: Integrin alphaIIb/beta3 (αIIbβ3) receptor complex, vWF: von Willebrand factor, GP6: Glycoprotein VI, GP1BA: Glycoprotein 1b subunit alpha, GP5: Glycoprotein V, GP9: Glycoprotein IX, SERPINE1: Serpin family E member 1, SERPINE2: Serpin family E member 2, SERPING1: Serpin family G member 1, PLAUR: Plasminogen activator urokinase receptor, F13A1: Coagulation factor XIII A chain, SELP: P-selectin, CD40, CD40LG: CD40 Ligand.
