# Supplementary material for: Pattern recognition receptor-associated immuno-thrombotic transcript changes in platelets and leukocytes with COVID19
Source: PLoS Pathog. 2025 Aug 18;21(8):e1013413. doi: 10.1371/journal.ppat.1013413 (PMC12373281; doi:10.1371/journal.ppat.1013413)
Supplement: S18 Table — (n = 17). (DOCX) [file ppat.1013413.s020.docx]

**Table S17**: Whole blood correlations in expression between Toll-like receptor or retinoic acid-inducible gene I receptor and prothrombotic or coagulation-related gene transcripts among COVID19 patients. (n=334)

|  | **TLR1** | **TLR2** | **TLR3** | **TLR4** | **TLR5** | **TLR6** | **TLR7** | **TLR8** | **TLR9** | **TLR10** | **RIG-I** | **MDA5** | **LGP2** | **cGAS** |
| --- | --- | --- | --- | --- | --- | --- | --- | --- | --- | --- | --- | --- | --- | --- |
|  |  |  |  |  |  |  |  |  |  |  |  |  |  |  |
| **Infected** |  |  |  |  |  |  |  |  |  |  |  |  |  |  |
|  |  |  |  |  |  |  |  |  |  |  |  |  |  |  |
| **ITGA2B** | -0.05 | **0.18** | **-0.43** | **0.14** | **0.32** | 0.02 | **-0.49** | **0.26** | **-0.30** | -0.11 | **-0.27** | **-0.29** | **-0.35** | **-0.37** |
|  | 0.35 | **7.9e-4** | **<1.0e-5** | **0.01** | **<1.0e-5** | 0.74 | **<1.0e-5** | **<1.0e-5** | **<1.0e-5** | 0.04 | **<1.0e-5** | **<1.0e-5** | **<1.0e-5** | **<1.0e-5** |
| **GP1BA** | 0.06 | **0.20** | 0.02 | **0.15** | 0.06 | **0.15** | -0.04 | 0.09 | **0.19** | **0.17** | 0.05 | 0.02 | 0.04 | -0.10 |
|  | 0.25 | **0.3e-4** | 0.67 | **7.5e-3** | 0.25 | **4.8e-3** | 0.47 | 0.11 | **5.2e-4** | **1.4e-3** | 0.38 | 0.77 | 0.42 | 0.05 |
| **GP1BB** | 0.03 | **0.18** | **-0.23** | **0.12** | **0.22** | 0.09 | **-0.27** | **0.20** | -0.03 | -0.02 | -0.06 | 0.07 | -0.12 | -0.10 |
|  | 0.57 | **1.3e-3** | **2.1e-5** | **0.03** | **3.9e-5** | 0.12 | **<1.0e-5** | **2.5e-4** | 0.57 | 0.71 | 0.28 | 0.23 | 0.04 | 0.06 |
| **GP9** | 0.04 | **0.27** | **-0.18** | **0.21** | **0.25** | 0.06 | **-0.24** | **0.15** | 0.02 | 0.01 | 0 | -0.04 | -0.04 | **-0.12** |
|  | 0.51 | **<1.0e-5** | **9.9e-4** | **1.1e-4** | **<1.0e-5** | 0.28 | **<1.0e-5** | **6.7e-3** | 0.78 | 0.83 | 0.97 | 0.46 | 0.50 | **0.03** |
| **GP5** | **-0.16** | **-0.13** | 0.05 | **-0.15** | **-0.14** | -0.02 | 0.03 | 0 | -0.11 | -0.07 | **-0.13** | **-0.12** | **-0.13** | **-0.21** |
|  | **4.30e-3** | **0.02** | 0.36 | **5.2e-3** | **0.01** | 0.78 | 0.62 | 0.96 | 0.05 | 0.20 | **0.02** | **0.03** | **0.02** | **1.5e-4** |
| **GP6** | 0.05 | **0.27** | 0.11 | **0.18** | **0.13** | 0.08 | 0.04 | 0.07 | **0.19** | 0.06 | **0.23** | **0.21** | **0.22** | 0.09 |
|  | 0.36 | **<1.0e-5** | 0.05 | **8.0e-4** | **0.02** | 0.13 | 0.50 | 0.19 | **6.8e-4** | 0.30 | **1.8e-5** | **1.7e-4** | **4.6e-5** | 0.10 |
| **PLAU** | **0.16** | **0.21** | **-0.31** | **0.16** | **0.31** | **0.18** | **-0.39** | **0.21** | 0.02 | 0.04 | **-0.13** | **-0.18** | **-0.26** | **-0.26** |
|  | **3.60e-3** | **7.9e-5** | **<1.0e-5** | **2.6e-3** | **<1.0e-5** | **1.1e-3** | **<1.0e-5** | **1.4e-4** | 0.68 | 0.47 | **0.02** | **7.6e-4** | **<1.0e-5** | **<1.0e-5** |
| **PLAUR** | **0.54** | **0.57** | **0.28** | **0.48** | **0.31** | **0.48** | **0.32** | **0.35** | **0.64** | **0.30** | **0.73** | **0.66** | **0.62** | **0.50** |
|  | **<1.0e-5** | **<1.0e-5** | **<1.0e-5** | **<1.0e-5** | **<1.0e-5** | **<1.0e-5** | **<1.0e-5** | **<1.0e-5** | **<1.0e-5** | **<1.0e-5** | **<1.0e-5** | **<1.0e-5** | **<1.0e-5** | **<1.0e-5** |
| **F13A1** | -0.10 | 0.03 | **-0.16** | 0.01 | 0.08 | 0.05 | **-0.19** | **0.19** | **-0.18** | -0.09 | **-0.22** | **-0.25** | **-0.30** | **-0.27** |
|  | 0.06 | 0.55 | **3.2e-3** | 0.79 | 0.13 | 0.32 | **6.6e-4** | **4.3e-4** | **1.2e-3** | 0.08 | **6.7e-5** | **<1.0e-5** | **<1.0e-5** | **<1.0e-5** |
| **SERPINE1** | **0.14** | **0.39** | 0.07 | **0.30** | **0.33** | 0.05 | -0.02 | **0.20** | 0 | 0.09 | **0.29** | **0.28** | **0.25** | 0.10 |
|  | **0.01** | **<1.0e-5** | 0.19 | **<1.0e-5** | **<1.0e-5** | 0.35 | 0.74 | **2.9e-4** | 1.00 | 0.12 | **<1.0e-5** | **<1.0e-5** | **<1.0e-5** | 0.06 |
| **SERPINE2** | 0.08 | **0.21** | 0.06 | **0.15** | 0.10 | 0.05 | -0.01 | 0.02 | 0.11 | **0.16** | **0.15** | **0.13** | **0.14** | 0 |
|  | 0.13 | **1.0e-4** | 0.28 | **4.6e-3** | 0.07 | 0.34 | 0.90 | 0.71 | 0.04 | **3.0e-3** | **7.4e-3** | **0.02** | **0.01** | 0.99 |
| **SERPING1** | **0.34** | **0.51** | **0.37** | **0.37** | **0.31** | **0.18** | **0.41** | **0.18** | **0.33** | **0.18** | **0.88** | **0.89** | **0.81** | **0.55** |
|  | **<1.0e-5** | **<1.0e-5** | **<1.0e-5** | **<1.0e-5** | **<1.0e-5** | **7.3e-4** | **<1.0e-5** | **1.1e-3** | **<1.0e-5** | **9.4e-4** | **<1.0e-5** | **<1.0e-5** | **<1.0e-5** | **<1.0e-5** |
| **TFPI** | 0.04 | **0.14** | **-0.21** | **0.17** | **0.27** | 0.08 | **-0.32** | **0.31** | **-0.33** | -0.01 | **-0.17** | **-0.21** | **-0.27** | **-0.25** |
|  | 0.43 | **0.01** | **1.2e-4** | **1.5e-3** | **<1.0e-5** | 0.16 | **<1.0e-5** | **<1.0e-5** | **<1.0e-5** | 0.91 | **1.9e-3** | **1.5e-4** | **<1.0e-5** | **<1.0e-5** |
| **PLAT** | 0.01 | **-0.16** | **0.69** | **-0.19** | **-0.41** | 0.03 | **0.64** | **-0.37** | **0.52** | **0.20** | **0.44** | **0.44** | **0.54** | **0.50** |
|  | 0.83 | **4.1e-3** | **<1.0e-5** | **3.7e-4** | **<1.0e-5** | 0.63 | **<1.0e-5** | **<1.0e-5** | **<1.0e-5** | **1.9e-4** | **<1.0e-5** | **<1.0e-5** | **<1.0e-5** | **<1.0e-5** |
| **F3** | 0.07 | 0.02 | 0.04 | 0.01 | 0 | 0.01 | 0.09 | 0 | 0.03 | 0.05 | 0.10 | 0.10 | 0.08 | **0.15** |
|  | 0.23 | 0.72 | 0.42 | 0.92 | 0.94 | 0.82 | 0.09 | 0.98 | 0.65 | 0.36 | 0.08 | 0.05 | 0.16 | **7.7e-3** |
| **PROC** | **0.27** | **0.23** | **0.29** | **0.16** | 0 | **0.31** | **0.32** | -0.05 | **0.72** | **0.39** | **0.40** | **0.33** | **0.40** | **-0.45** |
|  | **<1.0e-5** | **2.9e-5** | **<1.0e-5** | **4.0e-3** | 1.00 | **<1.0e-5** | **<1.0e-5** | 0.35 | **<1.0e-5** | **<1.0e-5** | **<1.0e-5** | **<1.0e-5** | **<1.0e-5** | **<1.0e-5** |
| **SERPINC1** | **0.39** | **0.34** | **0.39** | **0.29** | 0.11 | **0.35** | **0.31** | 0.04 | **0.63** | **0.48** | **0.51** | **0.43** | **0.50** | **0.55** |
|  | **<1.0e-5** | **<1.0e-5** | **<1.0e-5** | **<1.0e-5** | 0.05 | **<1.0e-5** | **<1.0e-5** | 0.41 | **<1.0e-5** | **<1.0e-5** | **<1.0e-5** | **<1.0e-5** | **<1.0e-5** | **<1.0e-5** |
| **VWF** | 0.11 | 0.04 | **-0.18** | 0.01 | 0.03 | 0.07 | **-0.17** | **0.14** | **-0.14** | -0.11 | **0.21** | **-0.23** | **-0.26** | **-0.26** |
|  | 0.04 | 0.44 | **7.8e-4** | 0.89 | 0.63 | 0.19 | **2.1e-3** | **0.01** | **0.01** | 0.05 | **8.4e-5** | **2.3e-5** | **<1.0e-5** | **<1.0e-5** |
| **SELP** | **0.12** | **0.33** | **-0.24** | **0.28** | **0.35** | **0.15** | **-0.26** | **0.30** | -0.04 | 0.06 | 0.02 | -0.02 | -0.08 | **-0.12** |
|  | **0.03** | **<1.0e-5** | **<1.0e-5** | **<1.0e-5** | **<1.0e-5** | **7.9e-3** | **<1.0e-5** | **<1.0e-5** | 0.42 | 0.27 | 0.72 | 0.76 | 0.13 | **0.03** |
| **SELPLG** | **0.61** | **0.68** | 0.09 | **0.68** | **0.50** | **0.63** | 0.04 | **0.59** | **0.47** | **0.46** | **0.51** | **0.41** | **0.41** | **0.41** |
|  | **<1.0e-5** | **<1.0e-5** | 0.11 | **<1.0e-5** | **<1.0e-5** | **<1.0e-5** | 0.49 | **<1.0e-5** | **<1.0e-5** | **<1.0e-5** | **<1.0e-5** | **<1.0e-5** | **<1.0e-5** | **<1.0e-5** |

Correlations were assessed by Spearman R (top value) and statistical significance (p<0.03, bottom value): TLR: Toll-like receptor, RIG-I: DDX58-RNA sensor RIG-I, MDA5: Melanoma differentiation-associated protein 5, LGP2: DHX58-DExH-box helicase 58, cGAS: Cyclic GMP-AMP synthase, ITGA2B: Integrin alphaIIb/beta3 (αIIbβ3) receptor complex, GP1BA: Glycoprotein 1b subunit alpha, GP1BB: Glycoprotein 1b subunit beta, GP9: Glycoprotein IX, GP5: Glycoprotein V, GP6: Glycoprotein VI, PLAU: Plasminogen activator urokinase, PLAUR: Plasminogen activator urokinase receptor, F13A1: Coagulation Factor XIII A Chain, SERPINE1: Serpin family E member 1, SERPINE2: Serpin family E member 2, SERPING1: Serpin family G member 1, TFPI: Tissue factor pathway inhibitor, PLAT: Plasminogen Activator Tissue Type, F3: Coagulation Factor III (Thromboplastin), PROC: Protein C, SERPINC1: Serpin Family C Member 1, vWF: Von Willebrand factor, SELP: P-selectin, SELPLG: P-selectin ligand.
