# Supplementary material for: Pattern recognition receptor-associated immuno-thrombotic transcript changes in platelets and leukocytes with COVID19
Source: PLoS Pathog. 2025 Aug 18;21(8):e1013413. doi: 10.1371/journal.ppat.1013413 (PMC12373281; doi:10.1371/journal.ppat.1013413)
Supplement: S19 Table — (n = 118). (DOCX) [file ppat.1013413.s021.docx]

**Table S18**: Whole blood correlations in expression between Toll-like receptor or retinoic acid-inducible gene I receptor and prothrombotic or coagulation-related gene transcripts among influenza patients. (n=17)

|  | **TLR1** | **TLR2** | **TLR3** | **TLR4** | **TLR5** | **TLR6** | **TLR7** | **TLR8** | **TLR9** | **TLR10** | **RIG-I** | **MDA5** | **LGP2** | **cGAS** |
| --- | --- | --- | --- | --- | --- | --- | --- | --- | --- | --- | --- | --- | --- | --- |
|  |  |  |  |  |  |  |  |  |  |  |  |  |  |  |
| **Infected** |  |  |  |  |  |  |  |  |  |  |  |  |  |  |
|  |  |  |  |  |  |  |  |  |  |  |  |  |  |  |
| **ITGA2B** | -0.30 | -0.19 | -0.21 | **-0.66** | -0.38 | -0.19 | -0.09 | **-0.63** | 0.39 | 0.20 | -0.47 | -0.31 | -0.15 | -0.20 |
|  | 0.24 | 0.46 | 0.42 | **4.6e-3** | 0.14 | 0.46 | 0.72 | **7.5e-3** | 0.12 | 0.45 | 0.06 | 0.22 | 0.57 | 0.43 |
| **GP1BA** | -0.36 | -0.05 | -0.19 | -0.49 | -0.34 | -0.05 | -0.22 | -0.48 | 0.39 | 0.16 | -0.50 | -0.37 | -0.26 | -0.17 |
|  | 0.16 | 0.84 | 0.46 | 0.05 | 0.18 | 0.85 | 0.40 | 0.05 | 0.12 | 0.53 | 0.05 | 0.15 | 0.30 | 0.52 |
| **GP1BB** | -0.12 | 0.02 | -0.27 | -0.48 | -0.13 | -0.04 | -0.01 | -0.41 | **0.52** | -0.04 | -0.14 | -0.08 | 0.21 | -0.02 |
|  | 0.65 | 0.94 | 0.30 | 0.05 | 0.62 | 0.89 | 0.98 | 0.10 | **0.03** | 0.88 | 0.59 | 0.77 | 0.42 | 0.94 |
| **GP9** | 0.28 | 0.30 | -0.15 | -0.12 | 0.21 | 0.25 | 0.17 | -0.07 | 0.48 | 0 | 0.15 | 0.21 | 0.48 | 0.19 |
|  | 0.27 | 0.25 | 0.56 | 0.65 | 0.42 | 0.34 | 0.52 | 0.79 | 0.05 | 1.00 | 0.57 | 0.42 | 0.05 | 0.46 |
| **GP5** | -0.42 | -0.26 | -0.17 | **-0.59** | **-0.63** | -0.33 | -0.09 | -0.45 | 0.41 | 0.03 | **-0.70** | -0.47 | -0.34 | -0.36 |
|  | 0.10 | 0.30 | 0.52 | **0.01** | **8.4e-3** | 0.19 | 0.72 | 0.07 | 0.11 | 0.91 | **2.5e-3** | 0.06 | 0.19 | 0.16 |
| **GP6** | -0.04 | 0 | -0.06 | -0.19 | -0.13 | 0.08 | -0.16 | -0.23 | 0.30 | 0.19 | -0.17 | -0.11 | 0 | -0.19 |
|  | 0.88 | 1.00 | 0.82 | 0.46 | 0.61 | 0.76 | 0.55 | 0.37 | 0.24 | 0.47 | 0.51 | 0.67 | 0.99 | 0.46 |
| **PLAU** | -0.01 | -0.16 | 0.08 | 0.08 | -0.10 | 0.14 | -0.33 | -0.17 | 0.19 | 0.32 | -0.32 | -0.32 | -0.43 | -0.34 |
|  | 0.97 | 0.55 | 0.77 | 0.76 | 0.69 | 0.59 | 0.20 | 0.52 | 0.46 | 0.20 | 0.21 | 0.21 | 0.09 | 0.18 |
| **PLAUR** | 0.43 | **0.55** | -0.25 | 0.19 | 0.52 | 0.45 | 0.23 | 0.29 | 0.34 | -0.36 | **0.57** | 0.41 | **0.85** | 0.34 |
|  | 0.08 | **0.02** | 0.33 | 0.46 | 0.04 | 0.07 | 0.37 | 0.26 | 0.19 | 0.15 | **0.02** | 0.10 | **<1.0e-5** | 0.19 |
| **F13A1** | -0.18 | -0.05 | 0.19 | -0.25 | -0.19 | -0.12 | 0.17 | -0.15 | 0.43 | 0.06 | -0.27 | -0.05 | -0.14 | 0.09 |
|  | 0.48 | 0.86 | 0.48 | 0.33 | 0.46 | 0.65 | 0.52 | 0.56 | 0.09 | 0.82 | 0.29 | 0.84 | 0.59 | 0.74 |
| **SERPINE1** | 0.14 | 0.14 | 0.08 | 0.10 | 0 | 0.36 | 0.08 | 0.09 | 0.37 | 0.28 | -0.11 | 0.03 | -0.05 | 0.13 |
|  | 0.60 | 0.59 | 0.78 | 0.71 | 1.00 | 0.15 | 0.77 | 0.72 | 0.14 | 0.28 | 0.67 | 0.92 | 0.85 | 0.63 |
| **SERPINE2** | -0.46 | -0.27 | -0.12 | **-0.62** | **-0.57** | -0.47 | -0.22 | **-0.53** | 0.42 | 0.24 | **-0.62** | -0.43 | -0.43 | -0.34 |
|  | 0.06 | 0.29 | 0.65 | **7.9e-3** | **0.02** | 0.06 | 0.40 | **0.03** | 0.10 | 0.36 | **7.4e-3** | 0.08 | 0.08 | 0.18 |
| **SERPING1** | 0.50 | **0.58** | -0.06 | 0.30 | **0.59** | 0.45 | 0.44 | 0.41 | 0.13 | -0.42 | **0.70** | **0.60** | **0.90** | 0.51 |
|  | 0.05 | **0.02** | 0.82 | 0.24 | **0.01** | 0.07 | 0.08 | 0.10 | 0.61 | 0.09 | **2.2e-3** | **0.01** | **<1.0e-5** | 0.04 |
| **TFPI** | 0.31 | 0.13 | **0.64** | 0.12 | 0.10 | 0.19 | **0.65** | 0.26 | -0.38 | -0.31 | 0.41 | **0.61** | 0.28 | 0.23 |
|  | 0.22 | 0.62 | **5.4e-3** | 0.65 | 0.71 | 0.47 | **5.0e-3** | 0.31 | 0.13 | 0.23 | 0.10 | **9.1e-3** | 0.28 | 0.37 |
| **PLAT** | -0.50 | -0.43 | 0.06 | **-0.61** | **-0.62** | -0.40 | 0.13 | -0.37 | -0.09 | -0.03 | -0.27 | -0.18 | -0.08 | -0.37 |
|  | 0.04 | 0.09 | 0.83 | **9.8e-3** | **8.0e-3** | 0.11 | 0.63 | 0.15 | 0.74 | 0.92 | 0.30 | 0.48 | 0.75 | 0.14 |
| **F3** | -0.25 | -0.38 | 0.13 | -0.10 | -0.36 | -0.33 | 0.10 | -0.07 | -0.29 | -0.14 | 0.16 | 0.02 | -0.08 | -0.01 |
|  | 0.33 | 0.14 | 0.63 | 0.69 | 0.15 | 0.20 | 0.71 | 0.78 | 0.26 | 0.58 | 0.55 | 0.95 | 0.76 | 0.97 |
| **PROC** | 0.14 | 0.40 | -0.19 | 0 | 0.16 | 0.38 | 0.14 | -0.02 | 0.20 | -0.02 | -0.03 | -0.06 | 0.12 | 0.40 |
|  | 0.59 | 0.11 | 0.46 | 0.99 | 0.53 | 0.13 | 0.60 | 0.94 | 0.45 | 0.94 | 0.92 | 0.83 | 0.65 | 0.11 |
| **SERPINC1** | -0.06 | 0.12 | -0.01 | -0.17 | 0.26 | 0.04 | 0.06 | -0.08 | -0.32 | -0.03 | 0.37 | 0.27 | 0.22 | 0.08 |
|  | 0.81 | 0.64 | 0.97 | 0.51 | 0.32 | 0.89 | 0.81 | 0.77 | 0.21 | 0.91 | 0.15 | 0.29 | 0.39 | 0.77 |
| **VWF** | **-0.58** | -0.35 | -0.17 | **-0.52** | **-0.64** | -0.30 | -0.02 | -0.41 | 0.42 | -0.47 | -0.45 | -0.42 | -0.13 | -0.15 |
|  | **0.02** | 0.16 | 0.52 | **0.03** | **6.4e-3** | 0.24 | 0.95 | 0.10 | 0.10 | 0.06 | 0.07 | 0.10 | 0.62 | 0.55 |
| **SELP** | -0.07 | 0.01 | -0.30 | -0.39 | -0.12 | 0.08 | -0.10 | -0.40 | 0.40 | -0.01 | -0.23 | -0.13 | 0.20 | -0.18 |
|  | 0.78 | 0.96 | 0.24 | 0.13 | 0.65 | 0.77 | 0.70 | 0.11 | 0.11 | 0.97 | 0.38 | 0.62 | 0.43 | 0.48 |
| **SELPLG** | 0.07 | 0.50 | -0.49 | -0.14 | 0.06 | 0.20 | -0.12 | -0.02 | **0.82** | -0.39 | -0.23 | -0.21 | 0.34 | 0.10 |
|  | 0.80 | 0.04 | 0.04 | 0.59 | 0.82 | 0.44 | 0.66 | 0.94 | **8.0e-5** | 0.13 | 0.37 | 0.42 | 0.18 | 0.70 |

Correlations were assessed by Spearman R (top value) and statistical significance (p<0.03, bottom value): TLR: Toll-like receptor, RIG-I: DDX58-RNA sensor RIG-I, MDA5: Melanoma differentiation-associated protein 5, LGP2: DHX58-DExH-box helicase 58, cGAS: Cyclic GMP-AMP synthase, ITGA2B: Integrin alphaIIb/beta3 (αIIbβ3) receptor complex, GP1BA: Glycoprotein 1b subunit alpha, GP1BB: Glycoprotein 1b subunit beta, GP9: Glycoprotein IX, GP5: Glycoprotein V, GP6: Glycoprotein VI, PLAU: Plasminogen activator urokinase, PLAUR: Plasminogen activator urokinase receptor, F13A1: Coagulation Factor XIII A Chain, SERPINE1: Serpin family E member 1, SERPINE2: Serpin family E member 2, SERPING1: Serpin family G member 1, TFPI: Tissue factor pathway inhibitor, PLAT: Plasminogen Activator Tissue Type, F3: Coagulation Factor III (Thromboplastin), PROC: Protein C, SERPINC1: Serpin Family C Member 1, vWF: Von Willebrand factor, SELP: P-selectin, SELPLG: P-selectin ligand.
